# Search for the Active Ingredients from a 2‐Aminothiazole DMSO Stock Solution with Antimalarial Activity

**DOI:** 10.1002/cmdc.202100067

**Published:** 2021-05-07

**Authors:** Henni‐Karoliina Ropponen, Chantal D. Bader, Eleonora Diamanti, Boris Illarionov, Matthias Rottmann, Markus Fischer, Matthias Witschel, Rolf Müller, Anna K. H. Hirsch

**Affiliations:** ^1^ Helmholtz-Institute for Pharmaceutical Research Saarland (HIPS) Helmholtz-Centre for Infection Research (HZI) Campus E8.1 66123 Saarbrücken Germany; ^2^ Department of Pharmacy Saarland University Campus E8.1 66123 Saarbrücken Germany; ^3^ Helmholtz International Lab for Anti-Infectives Campus E8.1 66123 Saarbrücken Germany; ^4^ Hamburg School of Food Science University of Hamburg Grindelallee 117 20146 Hamburg Germany; ^5^ Swiss Tropical and Public Health Institute Socinstrasse 57 4002 Basel Switzerland; ^6^ Universität Basel Petersplatz 1 4003 Basel Switzerland; ^7^ BASF-SE Carl-Bosch-Strasse 38 67056 Ludwigshafen Germany

**Keywords:** Antiprotozoal Agents, Decomposition, Drug Discovery, IspE, SFC

## Abstract

Chemical decomposition of DMSO stock solutions is a common incident that can mislead biological screening campaigns. Here, we share our case study of 2‐aminothiazole **1**, originating from an antimalarial class that undergoes chemical decomposition in DMSO at room temperature. As previously measured biological activities observed against *Plasmodium falciparum* NF54 and for the target enzyme *Pf*IspE were not reproducible for a fresh batch, we tackled the challenge to understand where the activity originated from. Solvent‐ and temperature‐dependent studies using HRMS and NMR spectroscopy to monitor the decomposition led to the isolation and in vitro evaluation of several fractions against *Pf*IspE. After four days of decomposition, we successfully isolated the oxygenated and dimerised compounds using SFC purification and correlated the observed activities to them. Due to the unstable nature of the two isolates, it is likely that they undergo further decomposition contributing to the overall instability of the compound.

## Graphical abstract

**False positive results** are unfortunately common in medicinal‐chemistry workflows. The unsuccessful parts of the stories are only rarely shared. In our decomposition study on an antimalarial 2‐aminothiazole, we observed degradation in DMSO. We successfully isolated by SFC and elucidated the structure of decomposition products contributing to the activities against the *Plasmodium falciparum* NF54 cell line and the target enzyme IspE.

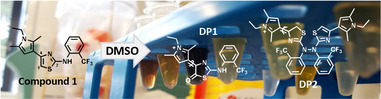

In the search for novel antimalarial compounds targeting the kinase IspE of the 2‐*C*‐methyl‐D‐erythritol 4‐phosphate (MEP) pathway, we identified a new 2‐aminothiazole class via an enzymatic high‐throughput screening (HTS) campaign (unpublished results from the consortium). The MEP pathway is a key biosynthetic route for the production of universal isoprenoid precursors.^[1]^ The HTS yielded compound **1** as a hit that was followed up due to its promising screening profile (Table 1), which was supported by some previously reported 2,4‐substituted thiazoles with antiplasmodial activity.[^2^, ^3^]

**Table 1:** Starting point for the non‐reproducible results of compound **1**.

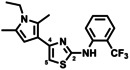		
	*Pf*IspE IC_50_ [μm]	*Pf*NF54 IC_50_ [μm]
Old batch^[a]^ (decomposed)	8.0±2.8	0.43±0.01
Fresh batch	>500	7.3±0.8

[a] Cytotoxicity profile of the old sample before becoming aware of the decomposition; %‐inh. A549=−0.3±4, HEK293=28±13 and HepG2=44±7, all @100 μM. *Pf*: *Plasmodium falciparum*

For a newly synthesised batch and the corresponding freshly prepared stock solution of compound **1**, no activity against *Plasmodium falciparum* (*Pf*) IspE was detected and cell‐based activity against the plasmodial strain *Pf*NF54 resulted in an 17‐fold reduction in activity (Table 1). Previous samples originated from older plates stored in DMSO, which had undergone several thawing cycles from −20 °C storage. First evidence of the decomposition was visually observed due to a change in colour of the compound's stock solution from clear to dark. However, this colour change would only be obvious to someone familiar with the original colour of the parent compound. If such plates are sent to collaboration partners responsible for biological assays, as is often the case in medicinal chemistry projects, such alterations may not necessarily be observed or questioned. DMSO is a widely used solvent due to its amphiphilic nature, enabling higher solubility of many compounds and high viscosity improving the reproducibility in pipetting. Nevertheless, stability issues of chemical compounds kept as stock solutions in DMSO are also acknowledged and spontaneous reactions, such as oxidation, cyclisation and hydrolysis, in stock solutions may affect the biological activity.[^4^, ^5^, ^6^] Although reactivity of 2‐aminothiazoles has not been directly ascribed to DMSO, we were concerned about it. 2‐Aminothiazoles are frequently‐hitting fragments in biophysical binding assays, as the so‐called promiscuous 2‐aminothiazoles (PrATs), and some sub‐categories are classified as Pan‐Assay Interference Compounds (PAINS).[^7^, ^8^, ^9^] The observed activity on target in vitro and in whole‐cell systems prompted us to elucidate the structural changes of compound **1** in DMSO that accounted for the increased inhibitory activity.

Firstly, we performed a temperature‐dependent decomposition study of compound **1** at 10 mM concentration in DMSO at room temperature (RT), +4 °C and −20 °C (SI, Section 2.3). After seven days, 64 % of the sample stored at RT had decomposed (SI, Table S1). Besides visual inspection of the colour changes of the test sample (see Table 2), the decomposition was monitored by HRMS and NMR spectroscopy. The sample that was incubated at −20 °C underwent only minimal decomposition, whereas, the sample kept at RT was fully decomposed after two weeks (SI, Figure S10). Furthermore, all three samples were tested for their inhibitory activity against *Pf*IspE after the incubation period. Only the samples incubated at RT and +4 °C showed measurable inhibitory activity in the *Pf*IspE assay, which was not observable for the freshly prepared compound **1** (Table 2). This fact correlated with the degree of chemical transformation of compound **1** (Table S1). In the IspE assay, the activity of the target enzyme is coupled to the oxidation of NADH (which can be followed spectrophotometrically at 340 nm) via a cascade of the auxiliary enzymes pyruvate kinase and lactate dehydrogenase (PK/LDH).^[10]^ Therefore, we next confirmed, whether the effects observed in the IspE assay are due to inhibition of the target enzyme or of the auxiliary enzymes. Three samples of compound **1** were tested in PK/LDH assay (Table 2). The resulting IC_50_ values of 34 μM and 45 μM for compound **1** incubated at RT and +4 °C, respectively, are only about three times higher than the IC_50_ values determined in the *Pf*IspE assay (12 μM and 16 μM, respectively). From these results, it was impossible to evaluate the influence of inhibition of auxiliary enzymes on the IC_50_ values determined in the *Pf*IspE assay.

**Table 2:** Temperature‐dependent decomposition of compound **1** in DMSO.

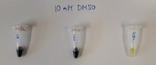				
*Activity measured* *after 3 months*	RT^[b]^	+4 °C^[b]^	−20 °C^[b]^	**1** ‐ Old sample^[c]^
*Pf*IspE IC_50_ [μm]^[a]^	12±4	16±7	>500	10±3
PK/LDH IC_50_ [μm]^[a]^	34±4	45±6	n.d.	40±4
*Ec*IspE IC_50_ [μm]^[a]^	101±14	71±10	>500	32±6
*Degradation after 2 months*	100%	79%	18%	N/A

[a] Errors given as formal standard error. [b] HRMS chromatograms measured shortly before the assay are given in SI, Figure S7. [c] The control values for the old decomposed sample run at the same time. n.d.: not determined, N/A: not applicable, PK/LDH: pyruvate kinase and lactate dehydrogenase, *Pf*: *Plasmodium falciparum*, *Ec*: *Escherichia coli*, RT: room temperature.

Another approach to address this issue is to perform the IspE assay with one or several orthologues of *Pf*IspE. The inhibitory potency of samples incubated at different temperatures of compound **1** against *Escherichia coli* (*Ec*)IspE are shown in the Table 2. The IspE assay setup was identical for both *Pf* and *Ec*IspE orthologs except for the target enzyme used. One of the setup requirements was that the enzymatic activity of the auxiliary enzymes PK/LDH exceeded the activity of the target enzyme IspE not less than a factor of ten.^[10]^ We investigated, if the inhibition of the auxiliary enzymes may substantially influence the IC_50_ values obtained from the coupled IspE assay. The IC_50_ values for the active batches (“RT” and “+4 °C”) of compound **1** observed in the *Ec*IspE assay are eight‐ and four‐fold less active in comparison to those observed in the *Pf*IspE assay (Table 2). The fact that the inhibition of auxiliary enzymes in the *Ec*IspE assay did not give rise to equally low IC_50_ values in comparison to those from *Pf*IspE assay means that the inhibition of auxiliary enzymes influenced only marginally the IC_50_ values measured in the *Pf*IspE assay.

Secondly, we analysed if spontaneous chemical transformation of compound **1** may depend on the solvent used for the preparation of its stock solutions. We incubated 10 mM stock preparations of compound **1** in DMSO, acetonitrile (ACN) and methanol (MeOH) at RT. Aliquots were taken during 16 days of incubation and tested against the enzyme *Pf*IspE (Table 3). Interestingly, no *Pf*IspE inhibitory activity for the ACN or MeOH aliquots was observed, meaning that no chemical transformation of compound **1** took place in ACN or MeOH, as supported by the HRMS and NMR data (SI, Section 2.4). For further decomposition incubation of compound **1**, only DMSO was used as solvent.

**Table 3:** Time‐dependent decomposition of compound **1** at room temperature.

	*Pf*IspE IC_50_ [μm] ^[a]^			PK/LDH IC_50_ [μm]^[a]^
Days of incubation	DMSO	ACN	MeOH	DMSO
0	>500	>500	>500	n.d.
5	19±7	>500	>500	n.d.
7	24±9	>500	>500	48±16
9	10±4	>500	>500	57±22
12	1±1	>500	>500	2±1
14	10±4	>500	>500	50±11
16	11±4	>500	>500	86±17
Solvent Blank	>500	>500	>500	n.d.

[a] Errors given as formal standard error. PK/LDH: pyruvate kinase and lactate dehydrogenase, *Pf*: *Plasmodium falciparum*. n.d.: not determined

The HRMS chromatographic profile of the analytical sample of compound **1** after 16 days of incubation at RT showed decomposition into several peaks, as seen in the chromatographic profiles of the samples previously used for the biological testing (SI, Figure S13). For further analysis, we repeated the incubation in DMSO on a larger scale for two weeks and fractionated the mixture by semipreparative HPLC into 29 fractions. The inhibitory activities of the fractions were determined against *Pf*IspE (SI, Table S2). In the preparative sample, a peak (*m/z*: 426.12607) was detected and interestingly, the sample incubated in DMSO‐*d_6_* included a peak at 4.86 min with the likely addition of the deuterated methyl groups to the mass (*m/z*: 432.16534), which initially attracted our attention due to a possible reaction with DMSO itself. However, this peak was isolated from the DMSO sample as fraction 4 in the semipreparative HPLC and it did not show any inhibitory activity against *Pf*IspE (SI, Table S2). Out of the isolated fractions, most of the active degradation products are very non‐polar and poorly separable by rp‐HPLC, showing dimerised masses as well as a mass of 380.10302//380.2 Da. The previous HRMS studies revealed that these signals appeared already after a couple of days of incubation at RT. In particular, we could observe that two peaks (*m/z*: 380.10257 and *m/z*: 365.11542) appeared after 15 hours and the latter becomes more prominent after four and a half days (SI, Figure S22).

Thus, we performed another round of large‐scale decomposition in DMSO at RT for five days and optimised the semipreparative HPLC conditions (SI, Table S3). A second purification step with supercritical fluid chromatography (SFC) (SI, Section 2.6) finally yielded two main active degradation products in sufficient amount for NMR analysis: decomposition product (**DP1**) with the *m/z* value of 380.10376 [*M*+H]^+^ or [*M*]^+^, corresponding to the sum formula of C_18_H_16_F_3_N_3_OS or respectively C_18_H_17_F_3_N_3_OS^+^ (*Pf*IspE IC_50_=199±26 μm) and **DP2** with the *m/z* of 365.11542 [*M*+2H]^2+^, corresponding to the sum formula of C_36_H_35_F_6_N_6_S_2_^+^ (*Pf*IspE IC_50_=59±4 μm) (Table 4, Figure 1).

**Table 4:** The biological data of the SFC‐separated compounds and analytical samples over four days.

	IC_50_ [μm] ^[a]^			
Days of incubation	*Pf*IspE^[b]^	*Ec*IspE	PK/LDH	*Pf*NF54
0	>500	>500	n.d.	n.d.
1	>500	>500	n.d.	n.d.
2	>500	>500	n.d.	3.5±0.3
3	486±20	397^[c]^	n.d.	n.d.
4	99±23	75±16	95±21	2.7±0.3
DP1 “380”	199±26	n.d.	>500	n.i. (>20)
DP2 “365”	59±4	n.d.	37±4	2.1±0.2

[a] All compounds were dissolved in methanol shortly before the assay. [b] Freshly dissolved compound **1** (*Pf*IspE IC_50_=>500 μm). [c] Error given as formal standard error. n.d.: not determined, n.i.: no inhibition at the highest concentration tested, PK/LDH: pyruvate kinase and lactate dehydrogenase, *Pf*: *Plasmodium falciparum*, *Ec*: *Escherichia coli*, DP: decomposition product.

**Figure 1:**
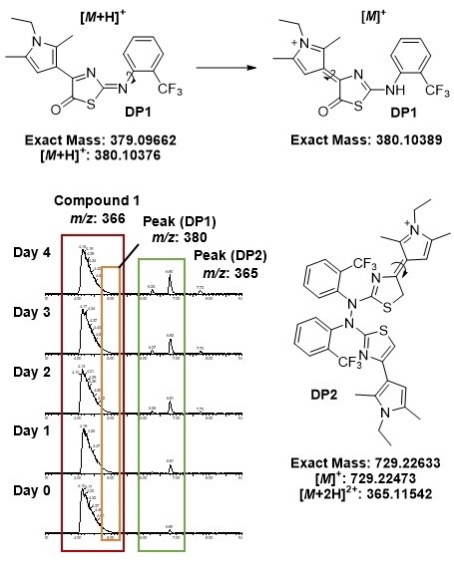
Characterised compounds isolated from the decomposition mixture. The shown chromatographic traces are base peak chromatograms generated with supercritical fluid chromatography.

**DP1** has a very distinct orange colour, supporting the formation of the formed oxygenated thiazolone core (UV_max_=196, 223 and 457 nm), (SI, Figure S32). The addition of the oxygen atom was evident based on the MS data and the exact position was confirmed by the disappearance of the characteristic −CH proton signal of the thiazole 5‐position at 6.54 ppm in the ^1^H NMR spectrum (SI, Table S4, Figure S24–S25) supported by a change in chemical shift of the neighbouring carbon atom in 4‐position from 141.3 ppm to 172.5 ppm. In the parent compound **1**, HMBC correlation of the thiazole carbon in 4‐position (141.3 ppm) with the proton 5‐position (6.52 ppm) is clear, whereas in the **DP1**, the thiazole carbon in 4‐position (172.5 ppm) correlates with the nearest methyl of the pyrrole (2.83 ppm) (SI, Figure S28–S29). Detection by UV afforded a single peak, which, however, underwent isomerisation into a more polar derivative over time. In fact, the long‐term stability of **DP1** became questionable as different stabilities were observed in acetone, MeOH, ACN, DMSO and CDCl_3_, (SI, Figure S33–37). Chloroform induces addition of chlorine as evidenced by the isotopic pattern, (*m/z*: 462.0 and 464.0 for ^35^Cl and ^37^Cl, respectively, Figure S35). The compound was most stable in acetone, in which we recorded NMR spectra and identified a mixture of compounds, as predicted based on the noticeable shift of the retention time in LCMS. The bond between the imine of the thiazolone core and the phenyl ring can rotate, as indicated in Figure 1, and thus, it is likely that **DP1** exists as either *E* or *Z* isomer. We calculated the energies of the lowest‐energy conformations, pointing towards an *E* configuration of the isolated **DP1** (+29.4 for *E* vs +33.2 kcal/mol for *Z*). Additionally, the appearance of a broad singlet at 5.84 ppm for ‐NH and the changes in the pyrrole shifts supports the formation of a charged pyrrolium species that can also exist either as *E* or *Z* isomer (+66.1 vs +62.8 kcal/mol). Considering the overall reactivity of the 2,4‐thiazole substitution, tautomerisation into a more stable conjugated form can occur due to the slightly acidic and dipolar nature of DMSO. This would mean that we measure the charged form in the HRMS as 380.10376 [*M*]^+^. Due to the overall stability of **DP1**, further experiments to confirm the exact isomeric mixtures are cumbersome and were not pursued. Nevertheless, the oxygenated **DP1** in its isomeric mixture from the SFC separation showed moderate, yet selective, inhibitory activity against *Pf*IspE (IC_50_=199±26 μm) without PK/LDH inhibition (IC_50_=>500 μm), but showed no inhibition in the *Pf* cell‐based assay at the highest tested concentration (IC_50_ >20 μm). Importantly, the cellular assay occurs over a longer time (72 hours) than the enzymatic assay (30 min), which may influence the overall stability of the compound. In addition, we monitored the HRMS chromatographic profile of freshly dissolved compound **1** in the enzyme assay buffer (Tris‐HCl, pH 7.6) with and without 5 % DMSO for 30 min (SI, Figure S16–17). The presence of DMSO seems to influence the formation of **DP1** (*m/z*: 380.10260 and 380.10238) and **DP2** (*m/z*: 365.11554), even within the short time window of 30 min.

Structure elucidation by NMR spectroscopy showed that the mass of **DP2** fits in fact to a dimer of **1** (Figure 1). We observed most often the doubly protonated species with *m/z* of 365.11542 [*M*+2H]^2+^ more intensely than the charged mass [*M*]^+^: 729.22473. With aid of the SFC, we found out that an isomer of **DP2** starts to form over the four days of incubation, as seen by the appearance of the minor peak, as highlighted in the green box in Figure 1. NMR measurements revealed its non‐symmetry as one of the characteristic −CH peaks corresponding to the thiazole position 5 disappeared and a new peak appeared at 3.96 ppm, integrating for −CH_2_ (SI, Table S5). This led us to question how the dimerisation would occur. Based on the NMR and MS data, we propose that an N−N bond formation occurs between the linker amines, as represented in the proposed reaction (SI, Scheme S1). N−N bonds are generally unstable, however, rather commonly occur in biological complexes.^[11]^ Due to the slightly acidic nature of DMSO, it is likely that the conjugated pyrrolium species is present and the imine N, as in tautomer of compound **1**, is attacked by the nucleophilic linker amine forming the N−N bond. The charged pyrrolium may also undergo isomerisation between *E* and *Z* isomers as observed in the SFC conditions, (SI, Figure S38). *Z* being slightly more stable than *E* (+144.7 vs +148.5 kcal/mol), hampering the exact assignment of the pyrrolium NMR peaks. For **DP2**, we could reach a similar enzymatic activity profile as for the initial decomposed starting points (*Pf*IspE IC_50_=59±4 μm), although also inhibiting the auxiliary enzymes (PK/LDH IC_50_=37±4 μm). The antimalarial activity (*Pf*NF54 IC_50_=2.1±0.2 μm) is also corresponding to an overall increase in activity as the decomposition occurs (Table 4). The dimerisation via N−N bond formation could also occur at other nitrogen atoms, supporting the other dimerised masses measured after the first rp‐HPLC (SI, Table S2).

Lastly, we investigated, whether the decomposition of compound **1** would also occur in Cyrene^TM^, similarly dipolar and aprotic as DMSO, yet a green solvent.^[12]^ Disappointingly, clear decomposition occurred even after one day incubation at RT, although showing different masses to the DMSO samples (SI, Figure S18). Interestingly, dominating peak (*m/z*: 476.16010, potentially with the addition of Cyrene^TM^ and loss of water) occurred at 4.68 min, where we previously observed the peak with DMSO (*m/z*: 426.12607) and DMSO‐*d*_6_ (*m/z*: 432.16534). This may be ascribed to the general reactivity of compound **1** and Cyrene^TM^, which may, nevertheless, still be an interesting choice for other compounds. Additionally, we screened whether decomposition would also occur in DMSO at RT for structurally closely derivatives **2**–**4**. After one day, compounds **2** and **3** followed similar pattern as compound **1** by dimerising mainly into the predicted N−N dimers (Figure 2), *m/z*: 653.21700 and *m/z*: 701.34344, respectively (SI, Figures S49–50), yet cleaner than compound **1**. In comparison, compound **4** showed no dimerisation, (SI, Figure S51). The substituents seem to define the overall reactivity of this class of 2‐aminothiazoles. For instance, compound **2** showed in the first screening moderate activity against *Pf*IspE (IC_50_=191 μm, from a single measurement) that was not reproducible later for a freshly dissolved batch (*Pf*IspE IC_50_>500 μm), reinforcing the need for careful handling of any related compounds.

**Figure 2:**
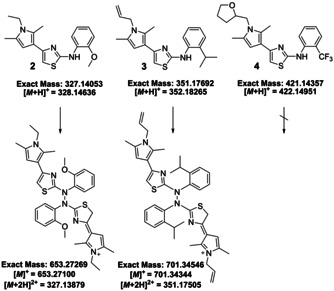
Summary of derivatives **2**–**4** with their proposed dimers of **2** and **3** based on the HRMS measurements (SI, Section 2.9).

In conclusion, we analysed the collected data to understand what causes the antimalarial activity that evolved from compound **1** stored in DMSO. The *Pf*IspE activity can be partly ascribed to dimerised **DP2**, although it also inhibits PK/LDH. On the other hand, the oxygenated **DP1** may also play a role when in its active isomeric form. The isolated degradation products themselves are unstable. They undergo further degradation, leading to a mixture that may also account for the observed activities. Additionally, our study underlines the importance of appropriate storage conditions of DMSO stock solutions of 2‐aminothiazoles. Based on the temperature‐dependent studies, we confirmed that decomposition hardly occurs at −20 °C over two months in DMSO. For the future, blocking the 5 position of the thiazole ring with a fluorine atom could be a feasible strategy to reduce the reactivity.^[13]^ However, to avoid decomposition, working with such a class requires preparation of fresh stock solutions prior to biological assays. Furthermore, multiple freeze‐thaw cycles should be avoided and alternative solvents should be considered. With this communication, we wish to remind the medicinal‐chemistry community again that what is in the test well, might not be the compound one thought.

## Experimental Section

Details for chemical syntheses, analytical and biological methods together with characterisation data are described in the Supporting Information.

## Conflict of interest

The authors declare no conflict of interest.

## Supporting information

As a service to our authors and readers, this journal provides supporting information supplied by the authors. Such materials are peer reviewed and may be re‐organized for online delivery, but are not copy‐edited or typeset. Technical support issues arising from supporting information (other than missing files) should be addressed to the authors.

Supplementary
