# Supplementary material for: Search for the Active Ingredients from a 2‐Aminothiazole DMSO Stock Solution with Antimalarial Activity
Source: ChemMedChem. 2021 May 7;16(13):2089–93. doi: 10.1002/cmdc.202100067 (PMC8360061; doi:10.1002/cmdc.202100067)
Supplement: Supplementary file 1 — Supplementary [file CMDC-16-2089-s001.pdf]

ChemMedChem

Supporting Information

Search for the Active Ingredients from a 2-Aminothiazole DMSO Stock Solution with Antimalarial Activity

Henni-Karoliina Ropponen, Chantal D. Bader, Eleonora Diamanti, Boris Illarionov, Matthias Rottmann, Markus Fischer, Matthias Witschel, Rolf Müller, and Anna K. H. Hirsch*

Table of Contents

1.) Biological Assays S2

1.1 General Procedure for Enzymatic Assay S2
1.2 General Procedure for Antimalarial Assay S2
1.3 General Procedure for Cytotoxicity Assay S2

2.) Decomposition Study Procedures S2

2.1 General Conditions S2
2.2 Synthesis of Compound **1** S4
2.3 Temperature-Dependent Study S8
2.4 Solvent-Dependent Study S10
2.5 Preparative HPLC Purification S15
2.6 SFC Purification S17
2.7 Characterisation of Decomposition Product **1** S19
2.8 Characterisation of Decomposition Product **2** S28
2.9 Screen of the Close Derivatives **2**, **3** and **4** S36

3.) References S38

4.) Appendix S39

4.1 Appendix I – Calibration of the HRMS Data S39
4.2 Appendix II – Extra NMR Spectra of the Other Isolated Fractions S40

1.) Biological Assays

1.1 General Procedure for Enzymatic Assay

The cloning, expression and purification of *Plasmodium falciparum*[1] and *Escherichia coli*[2,3] and their subsequent assays followed the previously reported procedures.[1] In some specified cases, the tested compound was dissolved in methanol or acetonitrile for the assay. Due to the nature of the study, some decomposition samples were only tested once and thus, for some formal standard error, determined from one IC_{50} curve with 5 to 10 data points, is given. For the final purified compounds, replicates were measured and standard deviation was calculated at least from two replicates.

1.2 General Procedure for Antimalarial Assay

Plasmodium falciparum drug-sensitive NF54 (airport strain from The Netherlands, provided by F. Hoffmann-La Roche Ltd) was cultivated in a variation of the medium consisting of RPMI 1640 supplemented with 0.5% ALBUMAX® II, 25 mM Hepes, 25 mM $NaHCO_3$ (pH 7.3), 0.36 mM hypoxanthine, and 100 µg/mL neomycin, as previously described.[4,5] Human erythrocytes served as host cells. Cultures were maintained in an atmosphere of 3% O_2, 4% CO_2, and 93% N_2 in humidified modular chambers at 37 °C. Compounds were dissolved in MeOH (10 mM), diluted in hypoxanthine-free culture medium and titrated in duplicates over a 64-fold range in 96 well plates. Infected erythrocytes (1.25% final hematocrit and 0.3% final parasitemia) were added into the wells. After 48 h incubation, 0.25 µCi of [^{3}H]hypoxanthine was added per well and plates were incubated for an additional 24 h. Parasites were harvested onto glass-fiber filters and radioactivity was counted using a Betaplate liquid scintillation counter (Wallac, Zurich). Chloroquine (IC_{50} = 3.1 ± 0.8 ng/mL) and artesunate (IC_{50} = 4.0 ± 1.7 ng/mL) were used as controls. The results were recorded and expressed as a percentage of the untreated controls. Fifty percent inhibitory concentrations (IC_{50}) were estimated by linear interpolation.[6]

1.3 General Procedure for Cytotoxicity Assay

Cytotoxicity assays based on the human lung adenocarcinoma (A549), human embryonic kidney (Hek293) and human hepatocellular carcinoma (HepG2) cell lines were performed as described previously.[7]

2.) Decomposition Study Procedure

2.1 General Conditions

All reagents and solvents were of commercial quality and used without further purification. Chemical yields were not optimised and the yields for the compounds isolated from the decomposition mixtures were not calculated. Low resolution mass analytics and purity controls were carried out using an Ultimate 3000-ISQ liquid-chromatography mass spectrometry (LCMS) system (Thermo Fisher Scientific, Dreieich, Germany) consisting of a Dionex UltiMate pump, an autosampler, DAD detector and an ESI quadrupole mass spectrometer. NMR spectra were recorded on a Bruker AV 500 or Ascend 700 (^{1}H, 500 MHz or 700 MHz; ^{13}C, 126 MHz or 175 MHz; ^{19}F, 470 MHz) spectrometer. All spectra were measured in DMSO-*d*$_6$, methanol-*d*$_4$, acetone-*d*$_6$ or acetonitrile-*d*$_3$ to which reported chemical shifts in parts per million (ppm), where adjusted based on the residual protons as the internal standards, (DMSO-*d*$_6$, δ = 2.50, 39.51, methanol-*d*$_4$, δ = 4.87, 49.1, acetone-*d*$_6$, δ = 2.05, 29.32 or acetonitrile-*d*$_3$, δ = 1.94, 1.39, ^{1}H and ^{13}C respectively). Coupling constants (J) are given in Hertz (Hz) and following abbreviations were used for multiplicity (s = singlet, d =doublet, t = triplet, m = multiplet, br = broad and combinations of these). High-resolution mass spectra (HRMS) were obtained using a Thermo Scientific Q Exactive Focus Orbitrap system or a maXis 4G UHR-TOF (Bruker Daltonics) both coupled to a Dionex Ultimate 3000 RSLC and equipped with a standard electrospray ion (ESI) source. An Acquity UPLC® BEH C8, 150 x 2.1 mm, 1.7 µm column equipped with a VanGuard Pre-Column BEH C8, 5 x 2.1 mm, 1.7 µm (Waters, Germany) was used for measurements with the Orbitrap system, using a flow rate of 250 µL/min. The gradient of [A] H_2O + 0.1% FA and [B] ACN + 0.1% FA was kept at 10% B for 1 min and then increased

to 95% B over 4 min and kept at 95% B for 1.2 min, before returning to 10% B over 0.3 min and equilibration for 1 min. For measurements with the TOF system, an Acquity UPLC® BEH C18, 100 x 2.1 mm, 1.7 µm column equipped with a VanGuard Pre-Column BEH C18, 5 x 2.1 mm, 1.7 µm (Waters, Germany) was used. The flow rate was set to 600 µL/min using the same solvents as for the Orbitrap system and the column thermostated at 45 °C. The gradient started at 5% B for one minute, before increasing to 100% B in 9 minutes. The amount of B was kept at 100% B for one minute before returning to initial conditions and equilibration. The flow was split to 75 µL min^{-1} before entering the mass spectrometer, which was externally calibrated to a mass accuracy below 1 ppm. Mass spectra were acquired in centroid mode ranging from 150–2500 *m/z* at a 2 Hz scan rate. All mass spectra were measured in positive ionisation mode in a range from 120–500 *m/z* for the Orbitrap and 150-2500 *m/z* for the TOF. UV spectra were recorded by a DAD in the range from 200 to 600 nm.

Preparative reverse phase-high performance liquid chromatography (rp-HPLC) was performed using an UltiMate 3000 Semi-Preparative System (Thermo Fisher Scientific, Dreieich, Germany) equipped with a Phenomonex Luna® 5 µm C18(2) 100 Å LC Column (250 x 10 mm) thermostated at 45 °C. Separation was achieved using a linear gradient from 60% (A) ddH2O to 100% (B) acetonitrile over 22.5 minutes. Before ramping the gradient, an equilibration step at 40% B was performed for 1 min. The B content was kept for 1 min at 100% after the gradient, before returning to 40% in 0.5 min and a final equilibration for 5 min. The compounds were detected by UV absorption at 210 and 280 nm.

The compounds were separated on a Waters Prep 15 SFC System coupled to an Acquity QDa mass spectrometer equipped with a 5 µm Torus Diol 130 Å OBD Prep Column 250 x 19 mm thermostated at 40°C. Separation was achieved using a linear gradient of 25–45% ACN as a cosolvent over 10 minutes, after a 1 min equilibration step at 25% in the beginning. The percentage of cosolvent was kept at 45 % for 1 minute before returning to 25 % in 1 minute and reequilibration for 3 minutes. Flow rate was set to 15 mL/min and backpressure to 120 bar. Analytical measurements were conduceted on a 5 µm Torus Diol 130 Å OBD Prep Column 150 x 2.1 mm with a gradient from 5–55% ACN and a flow rate of 3 mL/min. All other parameters resemble the preparative measurements.

2.2 Synthesis of Compound 1

***N*-((2-(trifluoromethyl)phenyl)carbamothioyl)benzamide**

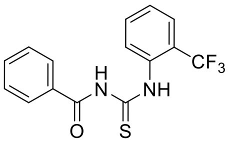

To a stirred solution of 2-(trifluoromethyl)aniline (1 eq., 0.77 g, 4,77 mmol) in acetone (16 mL), benzoyl isothiocyanate (1 eq., 0.64 mL, 4.77 mmol) was added and the reaction mixture was stirred to reflux for 1.5 h. The mixture was then poured into ice to obtain a yellowish precipitate (1.5 g, 99%). The compound was used without any further purification in the next step.

^{1}H NMR (500 MHz, DMSO-*d*$_6$): ***δ*** = 12.65 (s, 1H), 11.91 (s, 1H), 8.01 (dd, *J* = 8.4, 1.2 Hz, 2H), 7.79-7.83 (m, 1H), 7.81 (s, 1H), 7.74-7.79 (m, 1H), 7.73-7.78 (m, 1H), 7.66-7.71 (m, 1H), 7.52-7.59 ppm (m, 3H). *m/z* (ESI+) 325.06 [*M*+H]$^+$

1-(2-(Trifluoromethyl)phenyl)thiourea

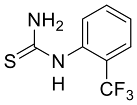

A solution of *N*-((2-(trifluoromethyl)phenyl)carbamothioyl)benzamide (1 eq., 1.5 g, 4.6 mmol) is refluxed in an aq. 10% NaOH solution (15 mL) for 1 hour. The reaction mixture was cooled to RT, acidified with aq. HCl 0.1 M and extracted with EtOAc (3 x 20 mL). The combined organic layers were dried over Na_2SO_4, filtered, concentrated in vacuo to afford as yellowish powder (0.57 g, 56%).

^{1}H NMR (500 MHz, DMSO-*d*$_6$): ***δ*** = 9.26 (s, 1H), 7.95 (m, 1H), 7.70 (m, 1H), 7.66 (t, *J* = 7.7 Hz, 1H), 7.51 (m, 2H), 7.49- 7.43 (m, 1H). *m/z* (ESI+) 221.04 [*M*+H]$^+$

4-(1-Ethyl-2,5-dimethyl-1*H*-pyrrol-3-yl)-*N*-(2-(trifluoromethyl)phenyl)thiazol-2-amine

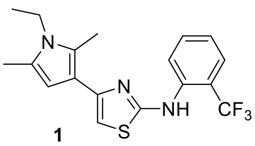

1

2-Chloro-1-(1-ethyl-2,5-dimethyl-1*H*-pyrrol-3-yl)ethan-1-one (1 eq., 0.100 g, 0.501 mmol) and 1-(2-(trifluoromethyl)phenyl)thiourea (1 eq., 0.110 g, 0.501 mmol) were dissolved in ethanol and heated to reflux for 6 h. The reaction mixture was cooled and the solvent removed under reduced pressure. The crude product was recrystallised from propan-2-ol to yield the pure compound **1** as light beige powder, (0.114 g, 0.312 mmol, 31%).

^{1}H NMR (500 MHz, DMSO-*d*$_6$): ***δ*** = 9.52 (br s, 1H), 7.98-8.13 (m, 1H), 7.73 (d, *J* = 7.8 Hz, 1H), 7.67 (br t, *J* = 7.5 Hz, 1H), 7.33 (br t, *J* = 7.5 Hz, 1H), 6.51 (br s, 1H), 5.96-5.99 (m, 1H), 3.80 (q, *J* = 7.2 Hz, 2H), 2.37 (s, 3H), 2.17 (s, 3H), 1.14 ppm (t, *J* = 7.2 Hz, 3H). ^{13}C NMR (126 MHz, DMSO-*d*$_6$): ***δ*** = 166.2, 138.0, 133.9, 126.9, 126.5, 125.9, 125.3, 124.8, 122.6, 111.5, 105.2, 99.2, 37.7, 15.9, 11.8, 10.9 ppm. ^{19}F NMR (470 MHz, DMSO-*d*$_6$): ***δ*** = -59.41 (s, 3F). HRMS (ESI+) calcd. for $C_{18}H_{18}F_3N_3S$ [*M*+H]$^+$: 366.12463, found: 366.12292.

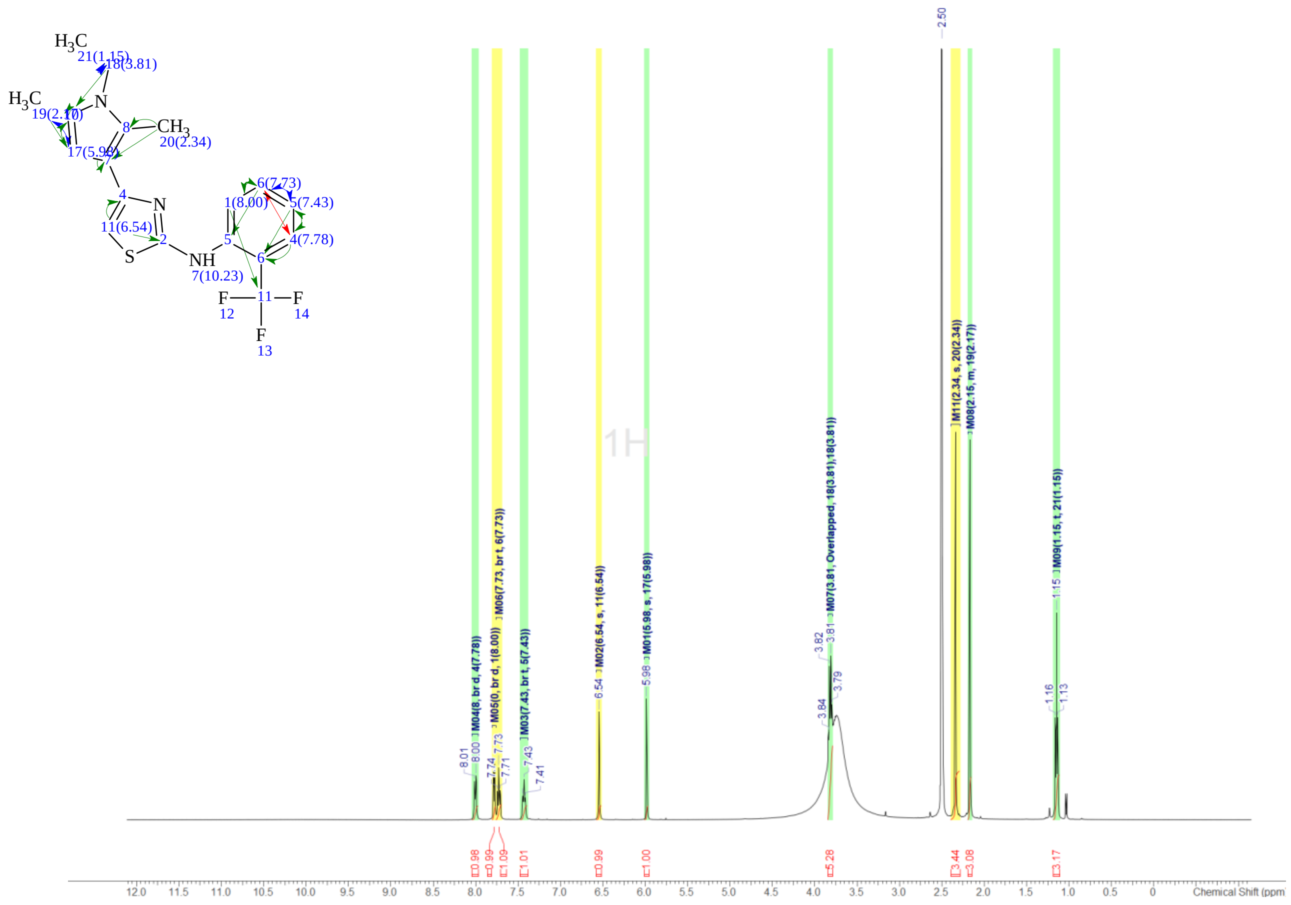

Figure S1. ^{1}H-NMR spectrum of compound **1** in DMSO-d_6.

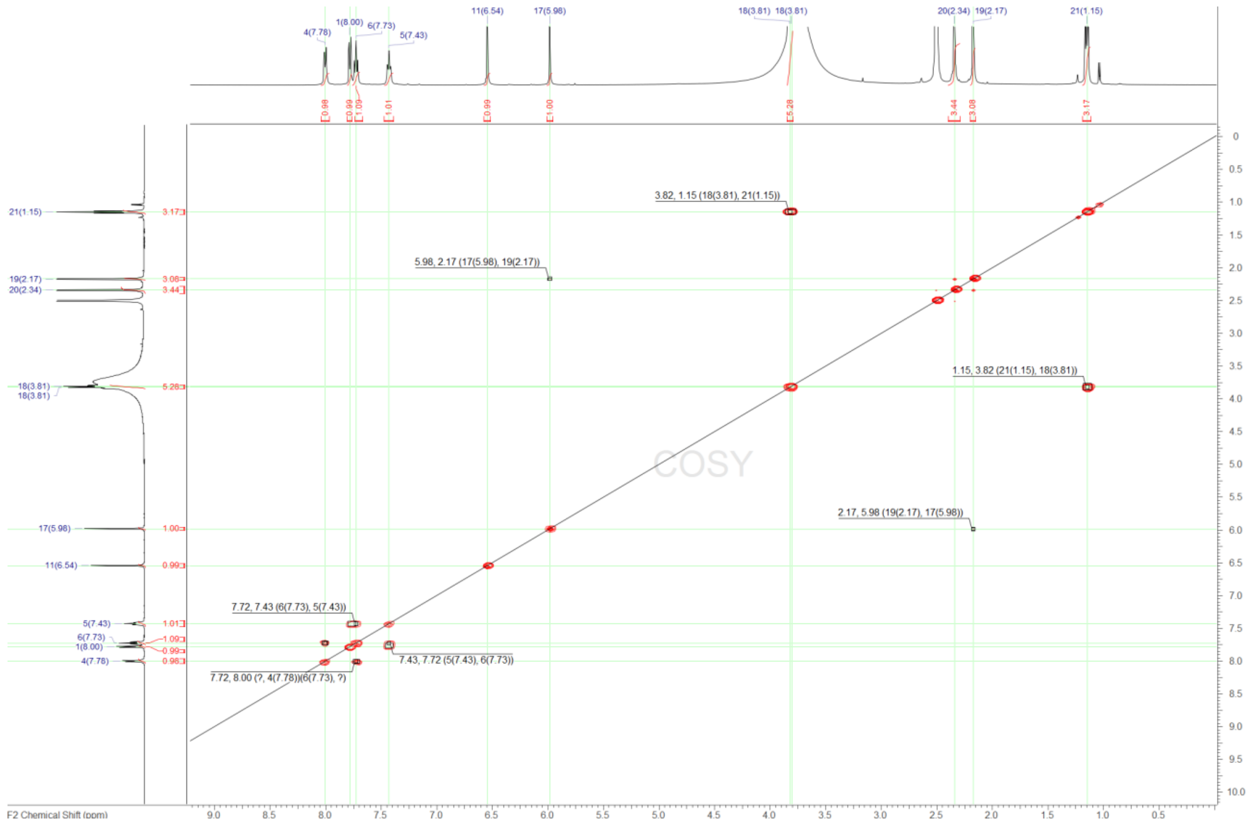

Figure S2. [^{1}H,^{1}H]-COSY NMR spectrum of compound **1** in DMSO-d_6.

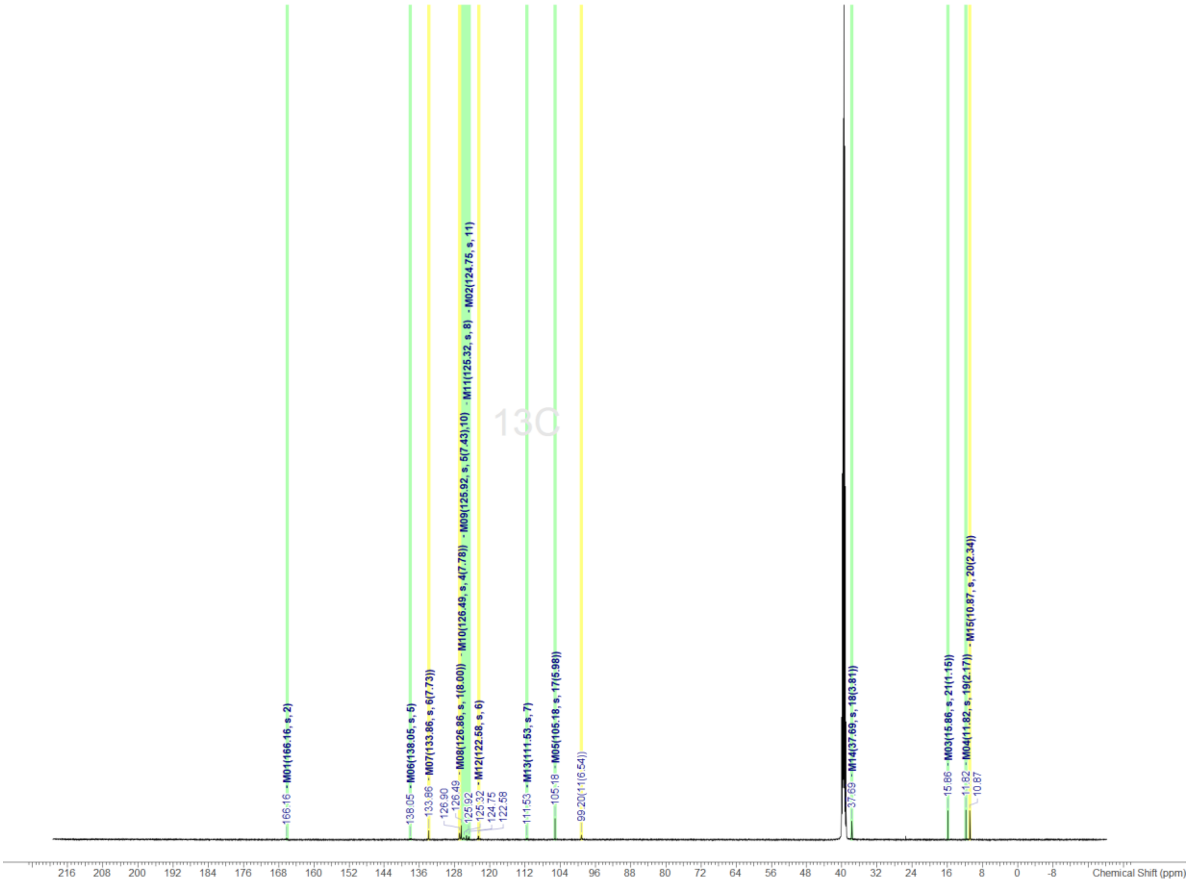

Figure S3. ^{13}C-NMR spectrum of compound **1** in DMSO-d_6.

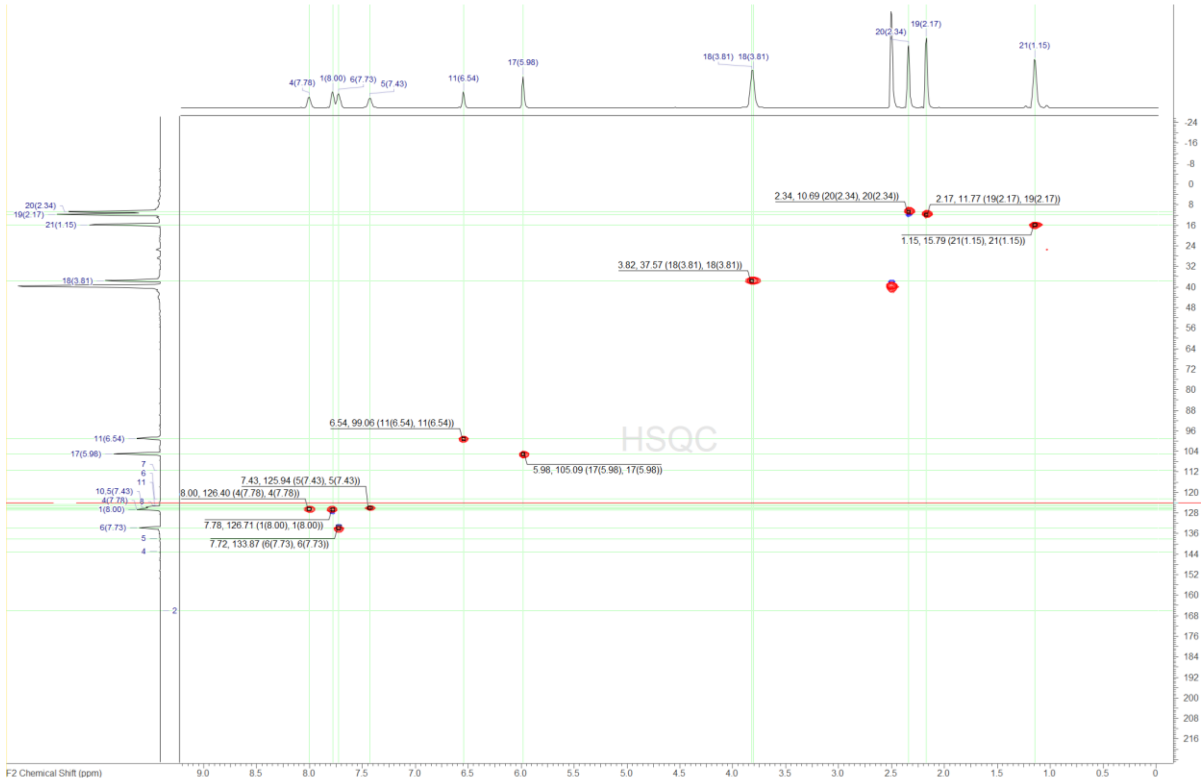

Figure S4. [^{1}H,^{13}C]-HSQC spectrum of compound **1** in DMSO-d_6.

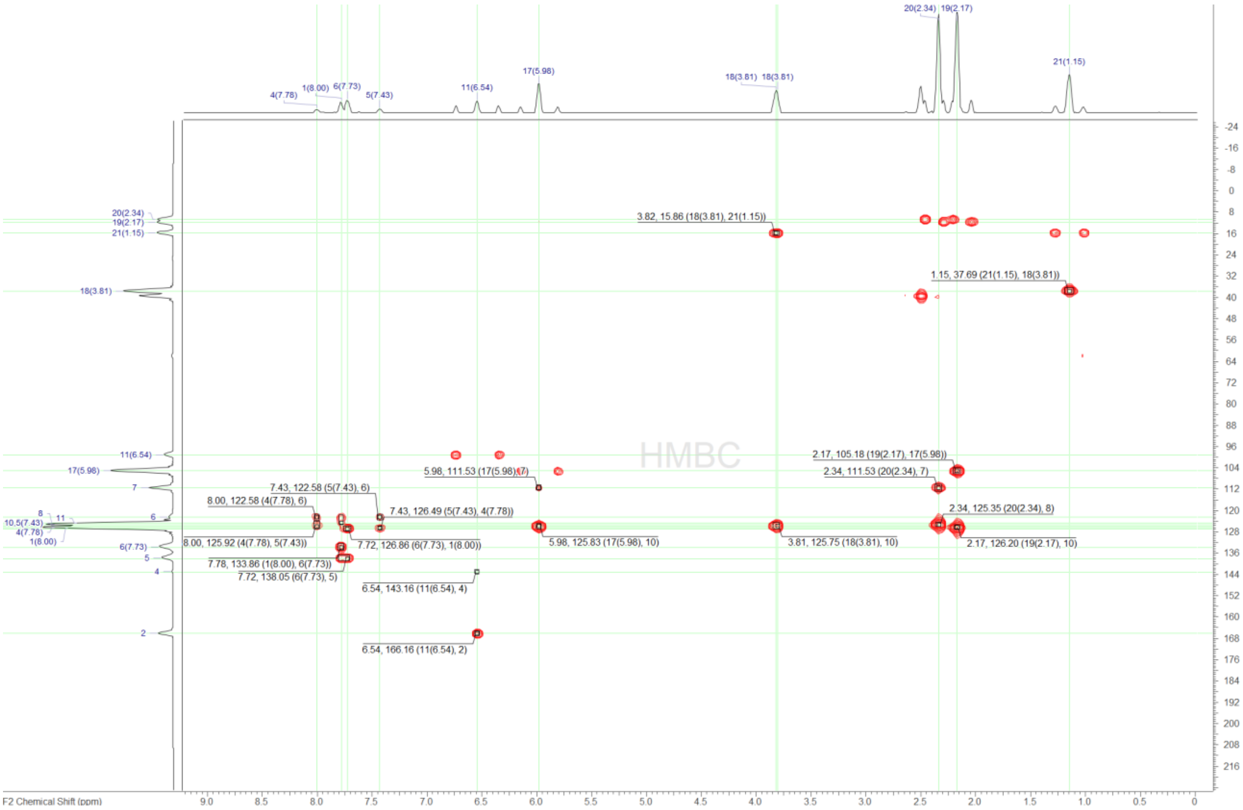

Figure S5. [^{1}H,^{13}C]-HMBC spectrum of compound **1** in DMSO-d_6.

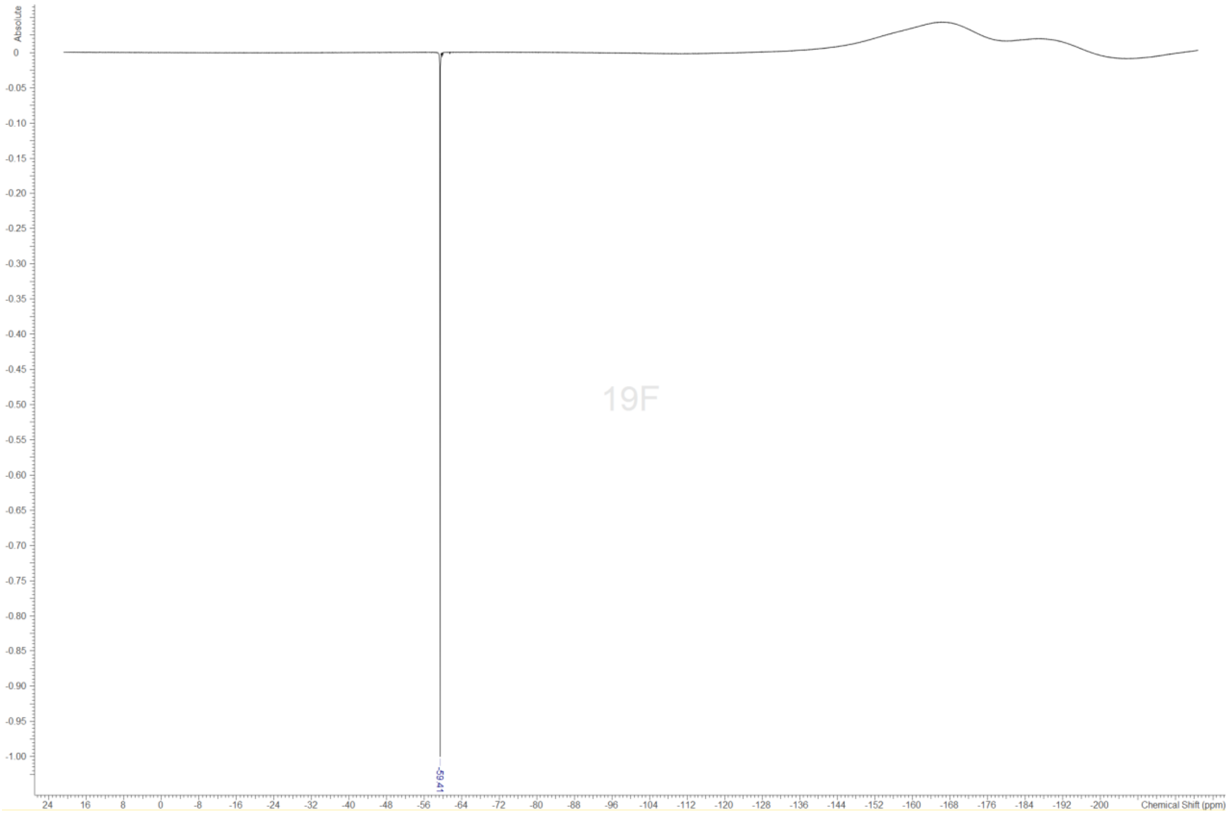

Figure S6. ^{19}F-NMR spectrum of compound **1** in DMSO-d_6.

2.3 Temperature-Dependent Study

Compound **1** was dissolved in DMSO to afford 10 mM stock solution, in the scale of 3.65 mg in 1 mL. Three aliquots were prepared and one each was stored inside a light-protecting box at different temperatures: room temperature (RT), +4 °C and –20 °C. Samples were only taken to RT for the minimum time required for sampling. Samples were further diluted with acetonitrile to 10 µM (1:1000) for HRMS analysis. For calibration, a fresh stock solution of the compound **1** was prepared shortly before the analysis. The concentration range used for the calibration was from 10 µM to 20 nM, including 10 samples with 1:1 dilutions starting from the highest concentration (Appendix I). In all of the calibration points and samples, diphenhydramine (*m/z*: 256.16907 [*M*+H]$^+$) was included as an internal standard at the concentration of 500 nM. The results were analysed using Thermo Xcalibur Quan Browser. Similarly, three NMR samples of compound **1** were prepared in DMSO-d_6 and each stored at different temperatures; RT, +4 °C and –20 °C, (Figure S8).

Table S1. Summary of the decomposition of compound **1** at different temperatures with the corresponding enzymatic activities measured after three months.

	The Percentage of Compound 1 Left After Degradation[a]			Enzymatic activities after 3 months of decomposition[b]		
**Temperature**	**Day 7**	**1 month**	**2 months**	***Pf*IspE IC$_{50}$ (µM)**	***Ec*IspE IC$_{50}$ (µM)**	**PK/LDH IC$_{50}$ (µM)**
**RT**	36%	1%	0%	12 ± 4	101 ± 14	34 ± 4
**+4 °C**	96%	48%	21%	16 ± 7	71 ± 10	45 ± 6
**–20 °C**	96%	91%	82%	>500	>500	n.d.

[a] The decomposition percentages are reported as the average of two values given in Tables S6. [b] Errors given as formal standard error. n.d.: not determined, PK/LDH: pyruvate kinase and lactate dehydrogenase, *Pf*: *Plasmodium falciparum, Ec: Escherichia coli.*

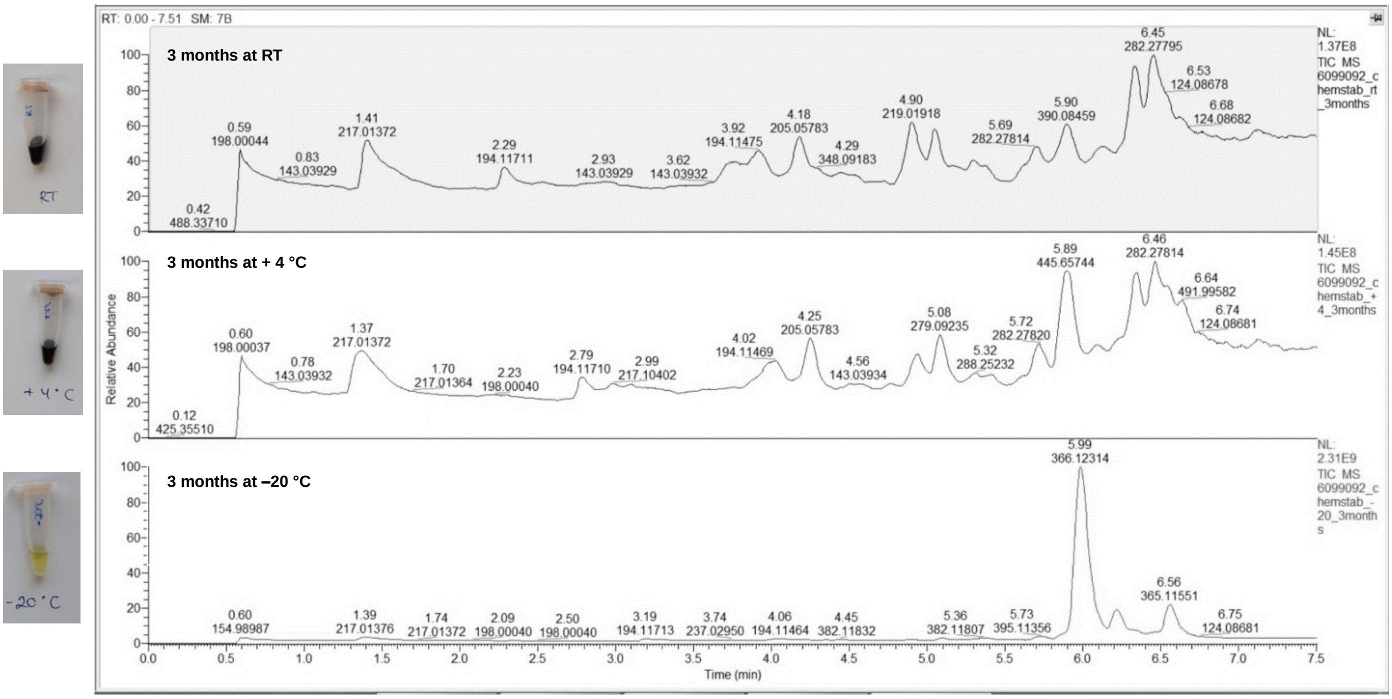

Figure S7. The HRMS recorded for the samples submitted to the enzyme assay after 3 months of decomposition with the corresponding colour differences.

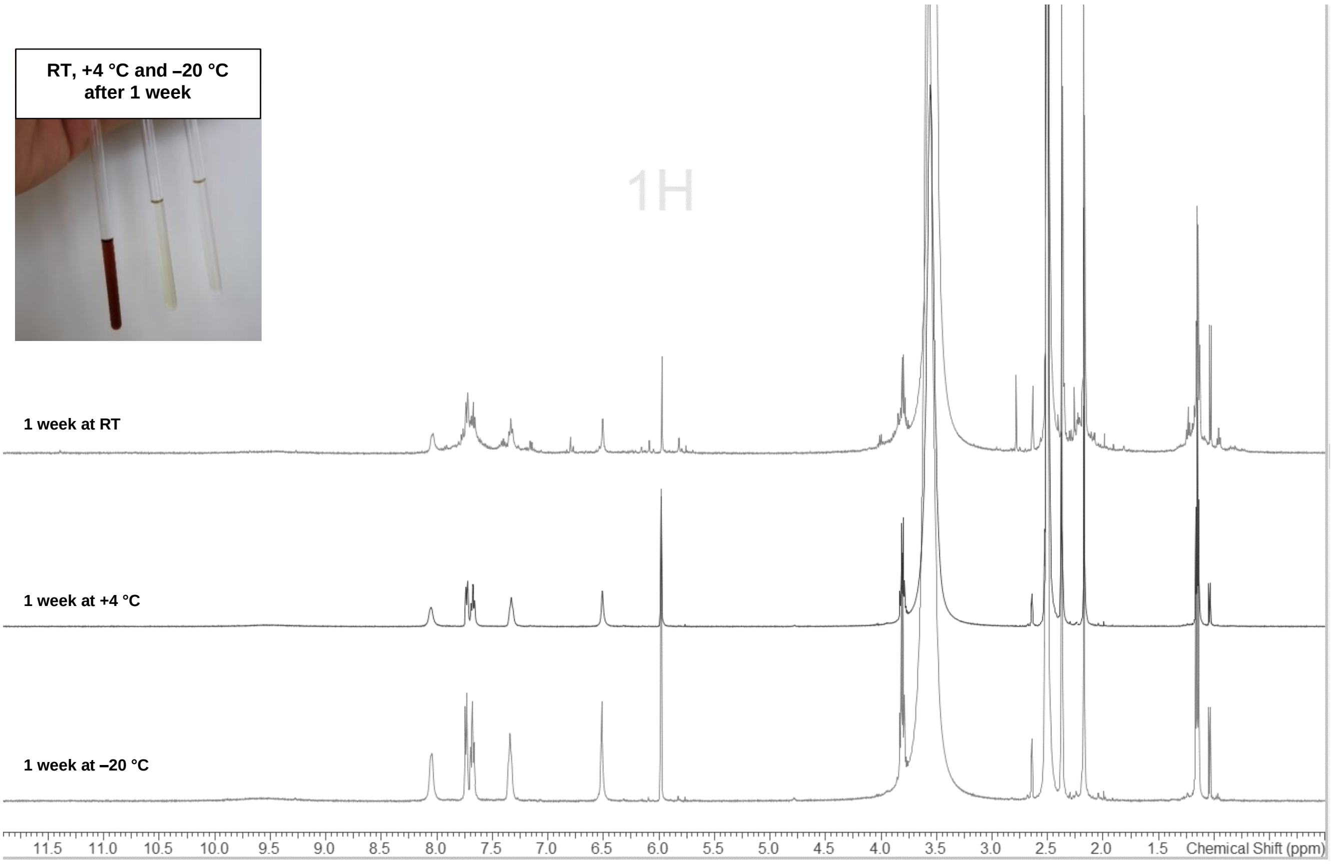

Figure S8. Decomposition of compound **1** in DMSO-d_6 samples at RT, +4 °C and –20 °C recorded with NMR after one week.

2.4 Solvent-Dependent Study

As in the Section 2.3, stock solutions of compound **1** were prepared in DMSO, ACN and MeOH and stored at RT for 16 days in a box protected from light. Samples were collected during this time and the enzymatic activity was analysed for them as in main text, Table 3. The samples were also diluted in ACN (1:1000) affording 10 µM solution and measured with HRMS, where no decomposition of compound **1** for ACN or MeOH was detectible (Figures S11–12). Similarly, the old samples of compound **1** used for enzyme and cell assays were analysed with HRMS (Figure S13). Additionally, NMR sample in MeOH was prepared and measured over time while stored at RT. As a follow-up experiment, the stability of compound **1** was determined for its 10 mM stock solution in Tris-HCl (pH 7.6) with and without 5% DMSO and Cyrene™ with HRMS (Figure S16–18).

Initial starting point **After 16 days**

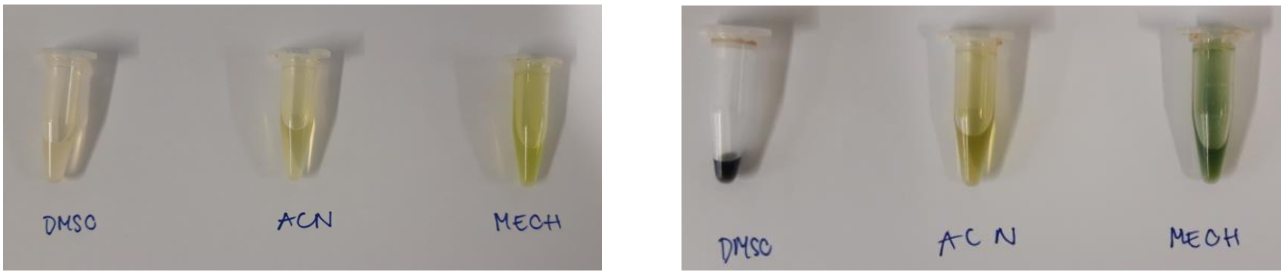

Figure S9. Colour differences of the decomposition samples in different solvent stored at RT.

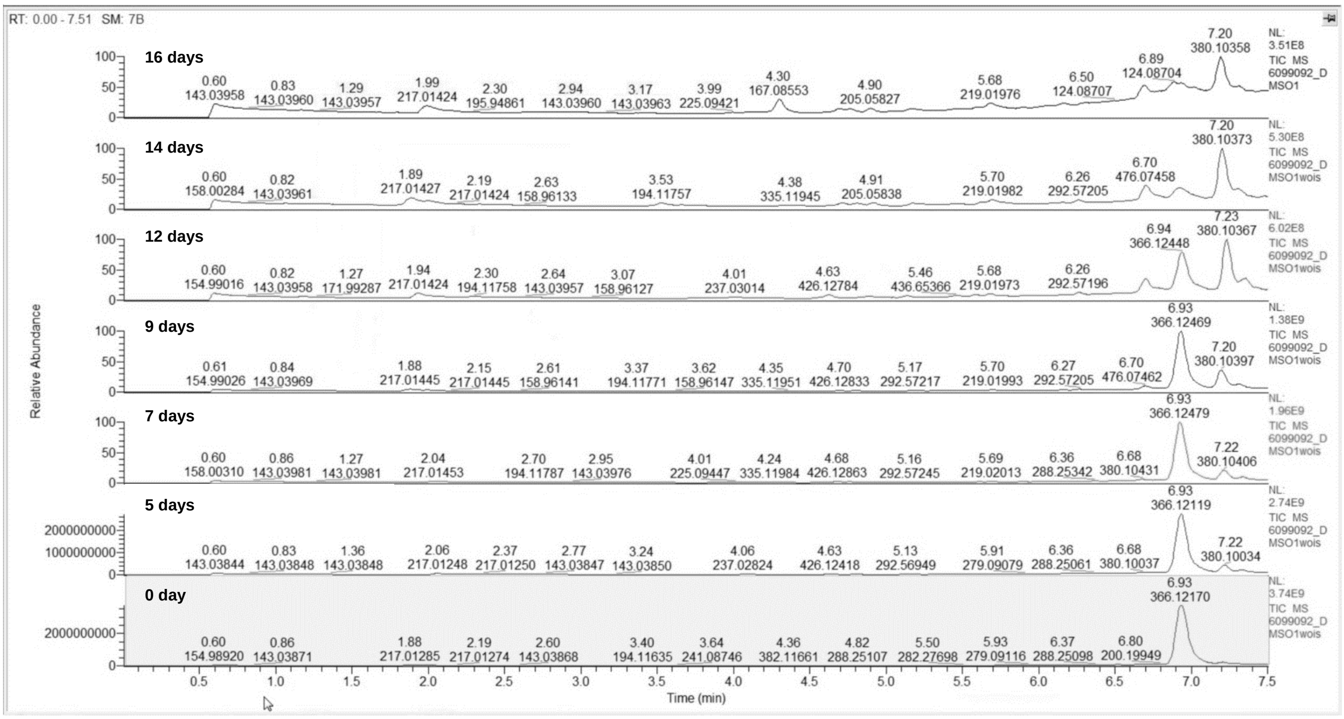

Figure S10. Decomposition of compound **1** studied in DMSO.

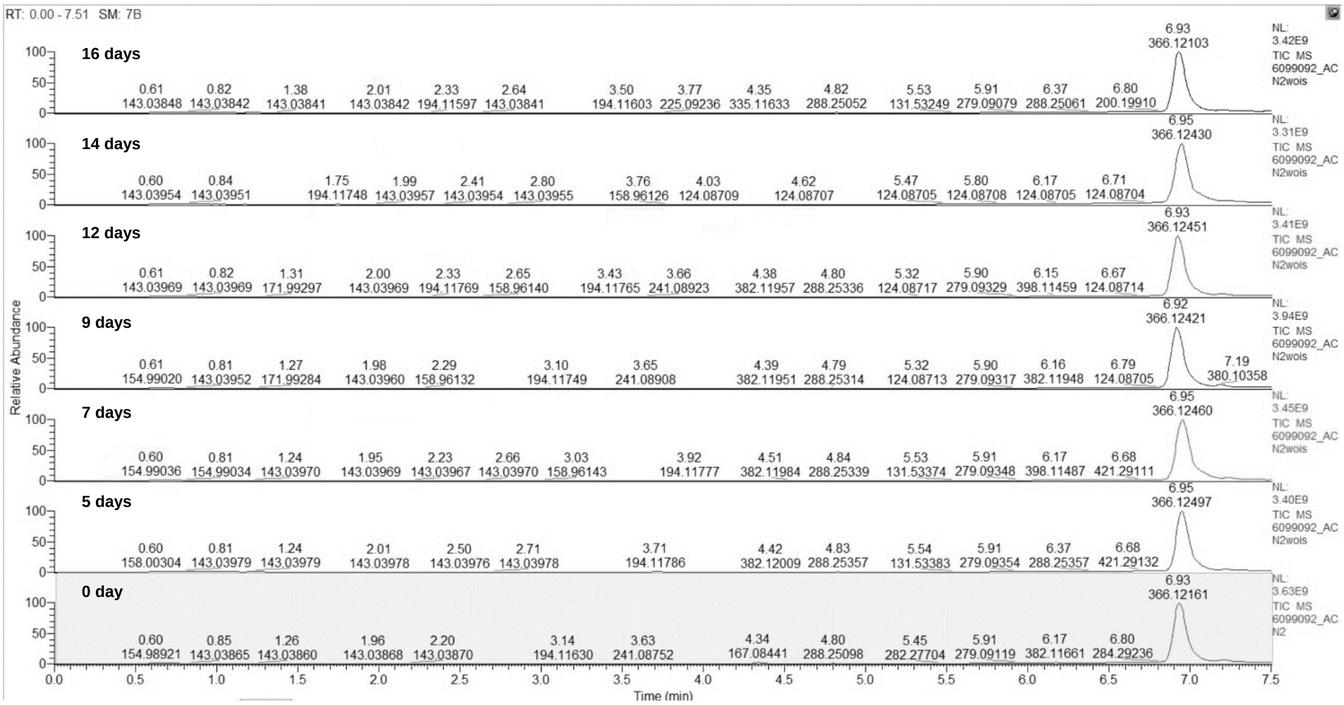

Figure S11. Decomposition of compound **1** studied in ACN.

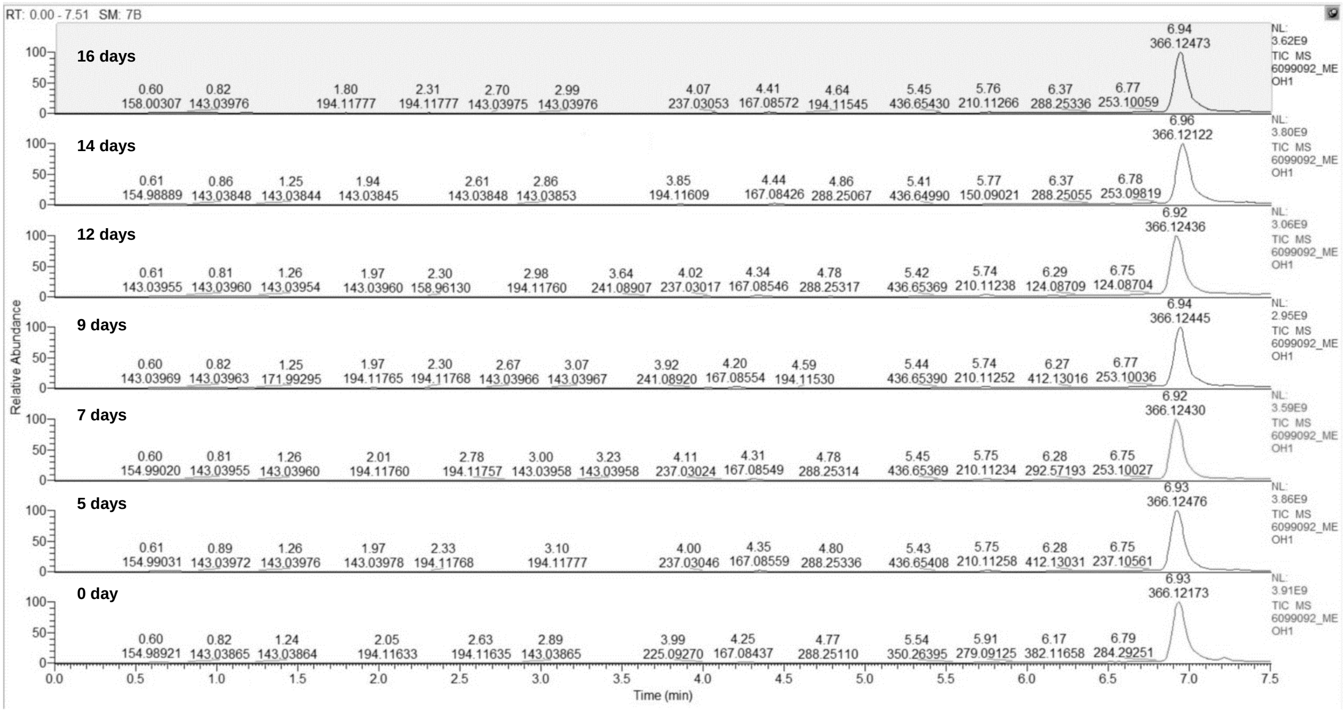

Figure S12. Decomposition of compound **1** studied in MeOH

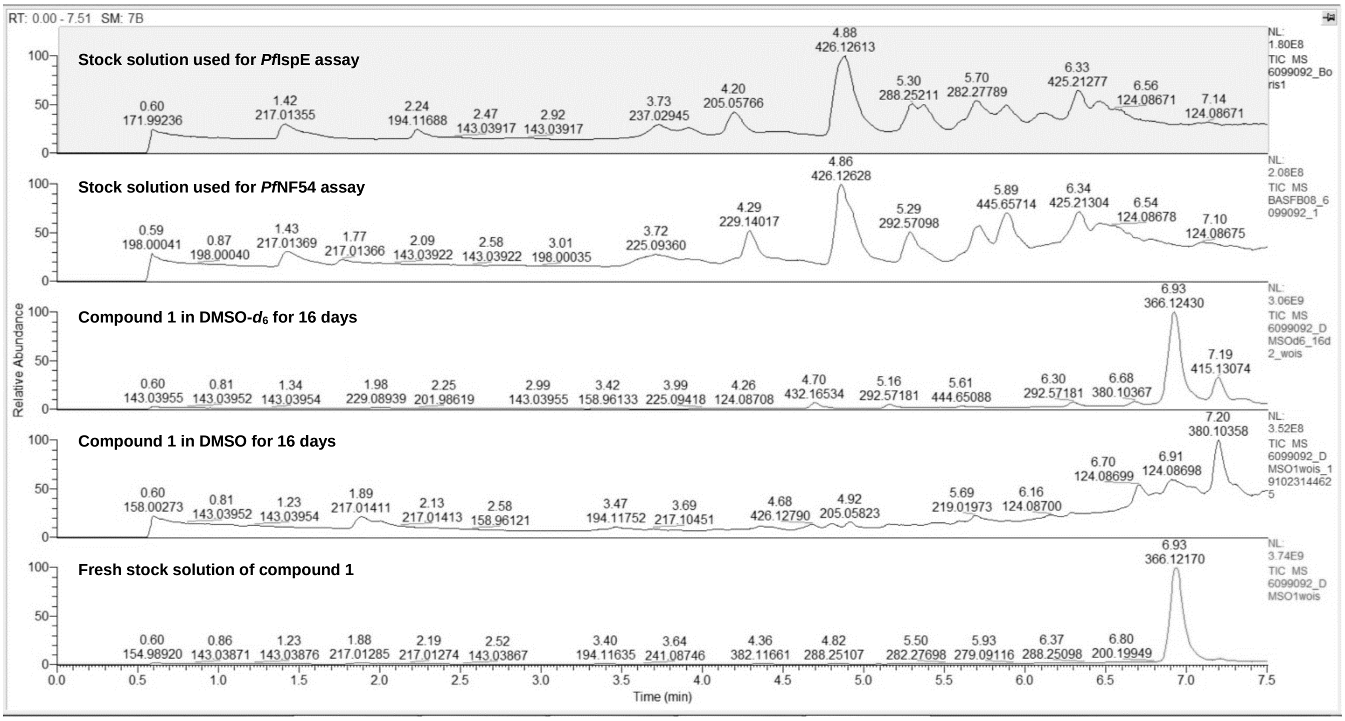

Figure S13. Comparison of the chromatographic profile of different samples. Note that the column conditions had changed in between the measurements.

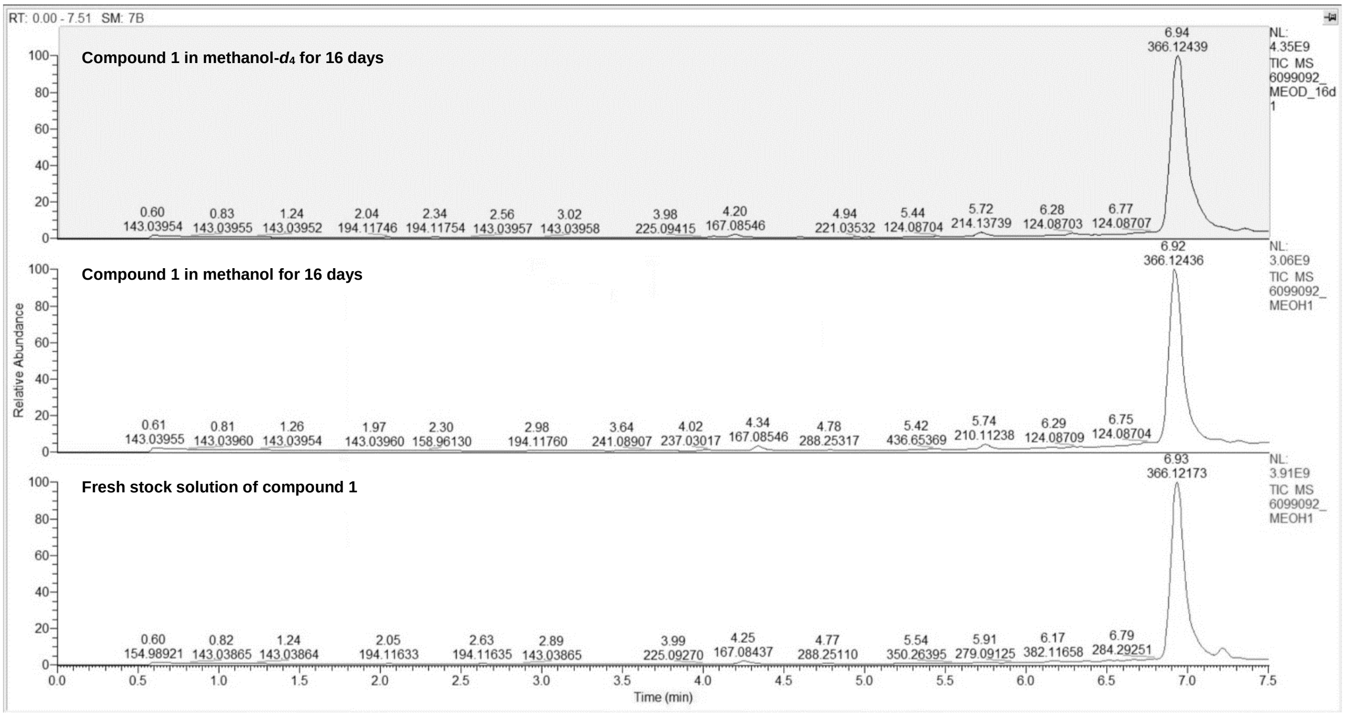

Figure S14. Decomposition of compound **1** studied in methanol-d_4.

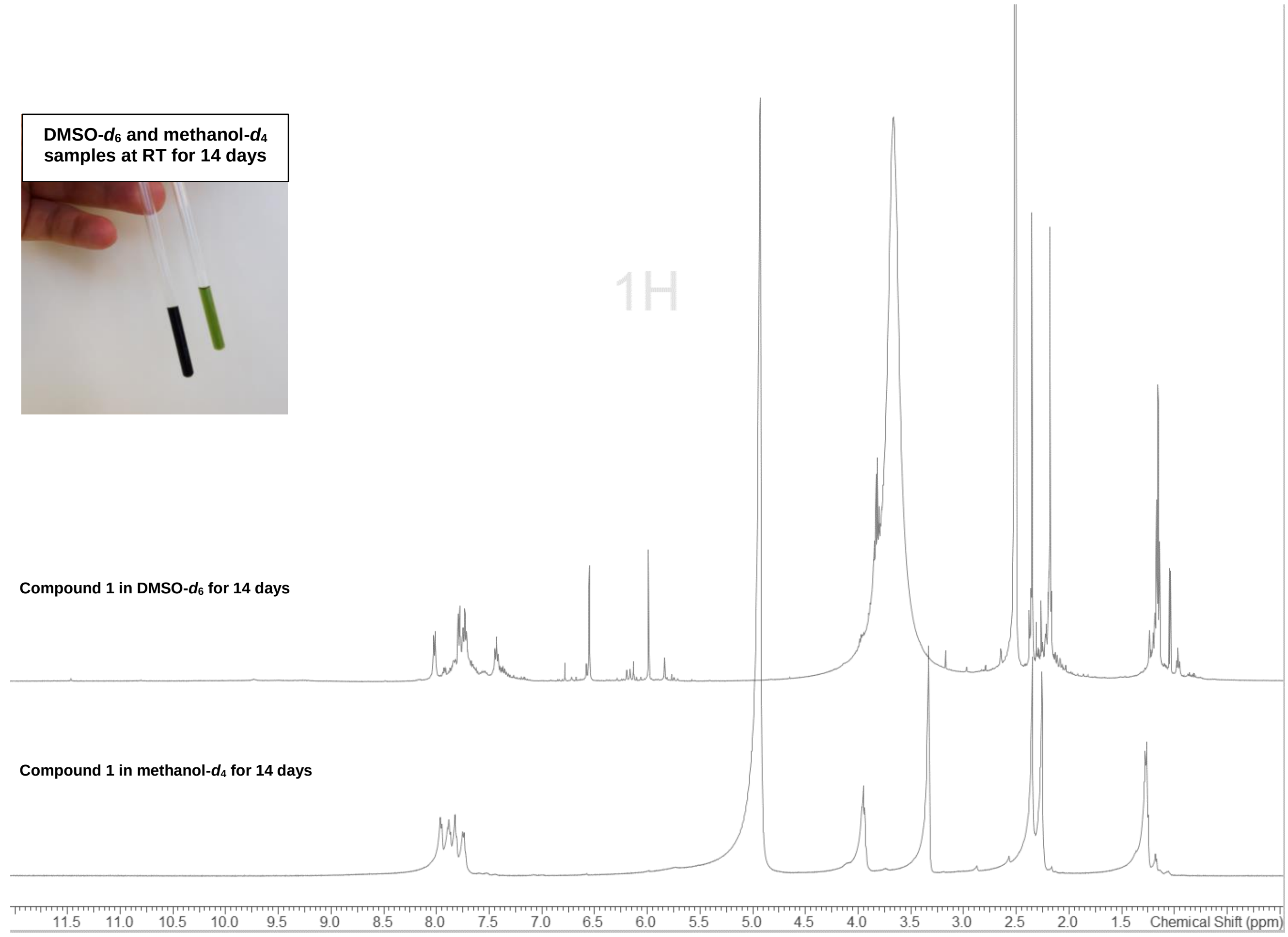

Figure S15. Decomposition of compound **1** studied in methanol-d_4 and DMSO-d_6.

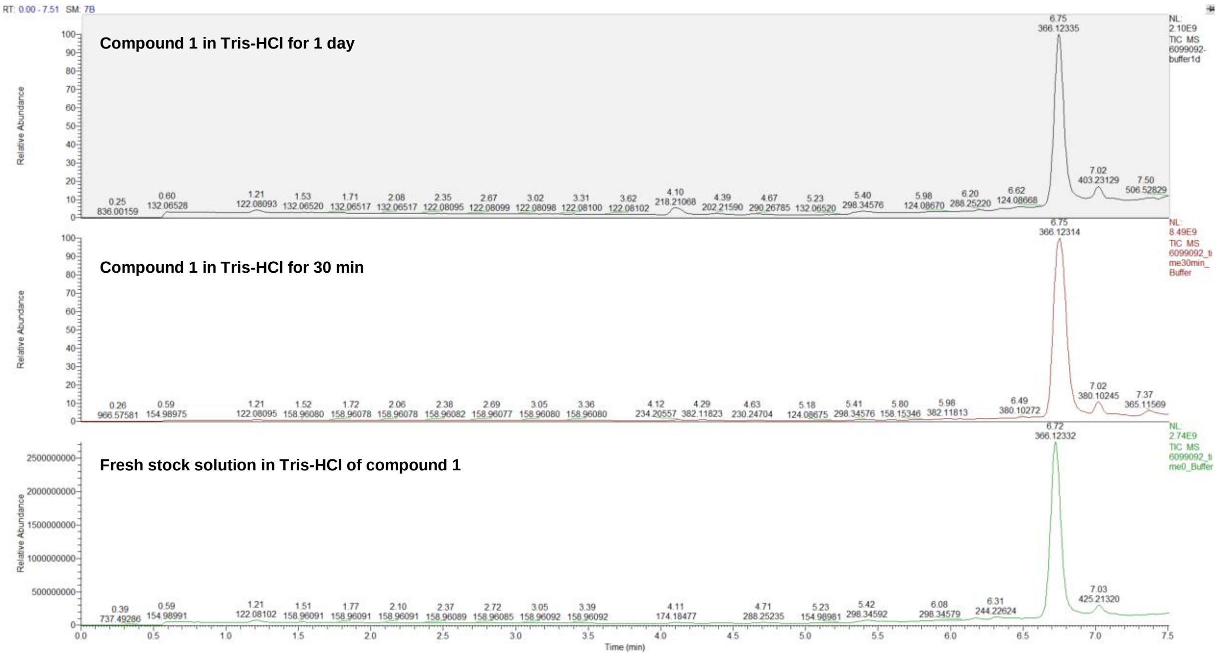

Figure S16. Decomposition of compound **1** studied in the enzyme assay buffer, Tris-HCl (pH 7.6).

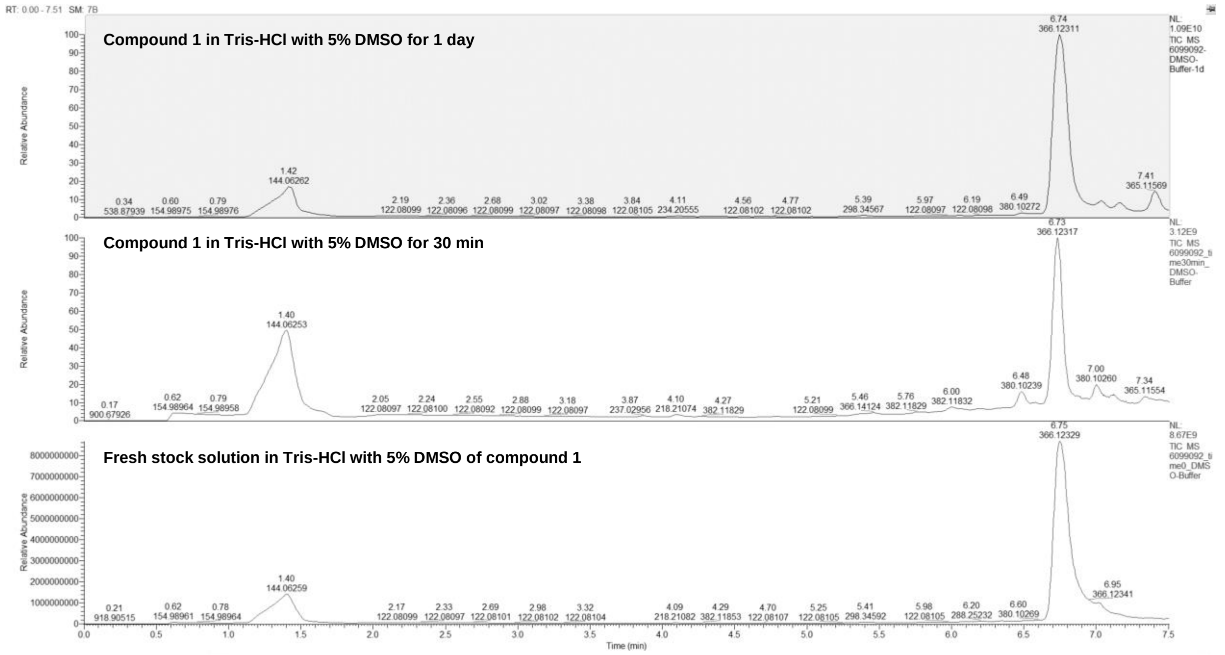

Figure S17. Decomposition of compound **1** studied in the enzyme assay buffer, Tris-HCl (pH 7.6) with 5% DMSO of the initial stock.

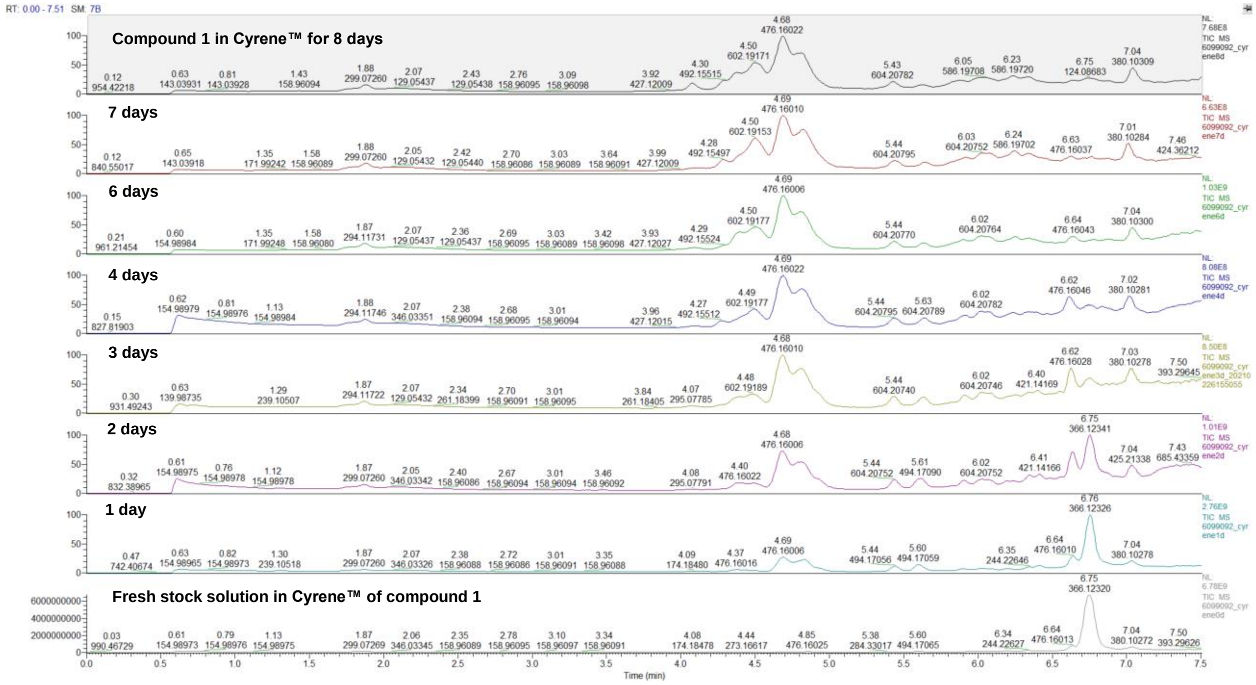

Figure S18. Decomposition of compound **1** studied in Cyrene™. The compound was incubated at RT in 10 mM stock solution.

2.5 Preparative HPLC Purification

Stock solution of 10 mM DMSO (in the scale of 3.65 mg in 1 mL, x3) was decomposed at RT for 2.5 weeks after which it was purified with prep. HPLC. The same was repeated for another set of samples after five days of decomposition of compound **1** in DMSO at RT.

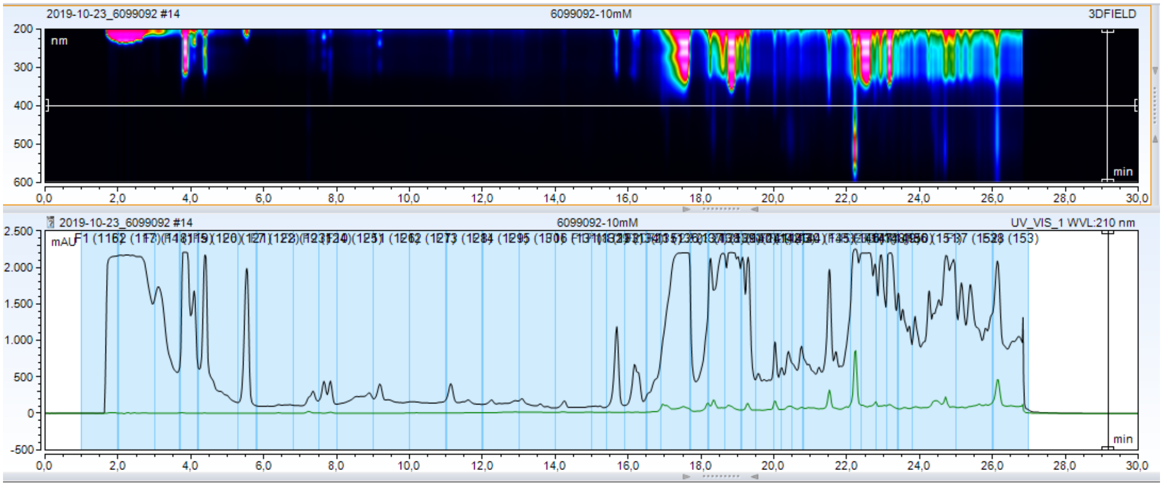

Figure S19. Chromatogram of the separation with the corresponding 3D-field.

Table S2. Separated fractions from the first prep. HPLC purification with the corresponding enzymatic activities.

Fraction	Weight (mg)	MS $[M+H]^+$	HRMS $[M+H]^+$	***Pf*IspE IC_{50} (µg/mL)[a,c]**	**PK/LDH IC_{50} (µg/mL)[a,c]**
**1_3**	0.6	-	n.d.	>500	n.d.
**4[d]**	0.4	426.1 // 205.1	426.12723 // 205.05789	>500	n.d.
**5**	0.2	426.1	426.12754	>500	n.d.
**6**	0.2	-	n.d.	>500	n.d.
**7**	0.4	-	n.d.	>500	n.d.
**8**	0.2	-	n.d.	>500	n.d.
**9_10**	0.3	426.1 // 210.0	426.12753 // 219.01955	>500	n.d.
**11_12**	0.5	426.1 // 472.1	426.12733 // 472.11520	>500	n.d.
**13_17**	3.9	472.1	472.11464	>500	n.d.
**18**	0.5	476.1	476.07387	>500	n.d.
**19**	0.6	-	n.d.	>500	n.d.
**20_21**	0.8	366.1 // 380.1 // 729.1 (364.1 $[M+2H]^{2+}$)	366.12414 //380.10388 // 727.20965 // 729.22360	22 ± 7	n.d.
**22**	0.3	584.1 // 757.1	584.13629 // 757.18403	>500	n.d.
**23**	0.5	584.1 // 380.1	584.13619 // 380.10333	>500	n.d.
**24**	1.1	584.1 // 412.1	584.13688 // 412.11331	>500	n.d.
**25**	0.5	380.1 // 428.1 // 743.2	380.10338 // 428.10676 // 743.20462	>500	n.d.
**26**	0.3	725.1	725.19351	>500	n.d.
**27**	0.4	725.1	725.19397	>500	n.d.
**28**	0.4	-	n.d.	>500	n.d.
**29**	0.3	-	n.d.	264 ± 250	n.d.
**30**	1.2	359.1 // 380.1 // 375.2	359.20286 // 380.10354 // 375.19847	66 ± 26	n.d.
**31[d]**	0.4	741.2	741.22560	14 ± 2	>500
**32**	1.6	373.2 // 586.4 // 380.1	373.23288 // 586.36900 // 380.10347	53 ± 16	n.d.
**33**	1.3	-	n.d.	>500	n.d.
**34**	0.4	775.2 // (388.1 $[M+2H]^{2+}$)	775.21228	41 ± 9	
**35**	0.4	-	380.10329	18 ± 3[b]	>500
**36**	0.6	-	380.10327 // 725.19059	7 ± 2[b]	>500
**37**	0.6	-	380.10302 //725.18974	7 ± 2	26 ± 3
**38**	0.5	-	380.10338 // 725.18974	20 ± 3	240 ± 114

[a] All fractions were dissolved in DMSO as 1 mg/mL. [b] *Ec*IspE IC_{50} (µg/mL) = >500. [c] Errors given as formal standard error. [d] NMR recorded for these two fractions (Appendix II). n.d.: not determined, PK/LDH: pyruvate kinase and lactate dehydrogenase, *Pf*: *Plasmodium falciparum. Ec: Escherichia coli.*

Table S3. Separated fractions from the second purification with the corresponding enzymatic activities.

Fraction	Weight (mg)	MS $[M+H]^+$	***Pf*IspE IC_{50} (µg/mL)[a]**	***Ec*IspE IC_{50} (µg/mL)[a]**	**PK/LDH IC_{50} (µg/mL)**
**1**	0.05	745.3	>500 (ACN)<br>>500 (MeOH)	>500 (ACN)<br>>500 (MeOH)	n.d.
**2**	0.05	380.1	>500 (ACN)<br>>500 (MeOH)	>500 (ACN)<br>>500 (MeOH)	n.d.
**3**	0.03	363.3	>500 (ACN)<br>>500 (MeOH)	>500 (ACN)<br>>500 (MeOH)	n.d.
**4**	0.10	365.2	>500 (ACN)<br>47 ± 17 (MeOH)	>500 (ACN)<br>123 ± 35 (MeOH) [b]	10 ± 2 (MeOH)[b]
**5**	0.05	381.3	>500 (ACN)[c]	>500 (ACN)[c]	n.d.

[a] All fractions were dissolved either in methanol (MeOH) or acetonitrile (ACN) to avoid further decomposition. [b] Errors given as formal standard error. [c] Only enough material for acetonitrile samples. n.d.: not determined, PK/LDH: pyruvate kinase and lactate dehydrogenase, *Pf*: *Plasmodium falciparum. Ec: Escherichia coli.*

2.6 SFC Purification

A stock solution of compound **1** in 10 mM DMSO (in the scale of 3.65 mg in 1 mL, x4) was decomposed at RT for four days after which it was purified with SFC.

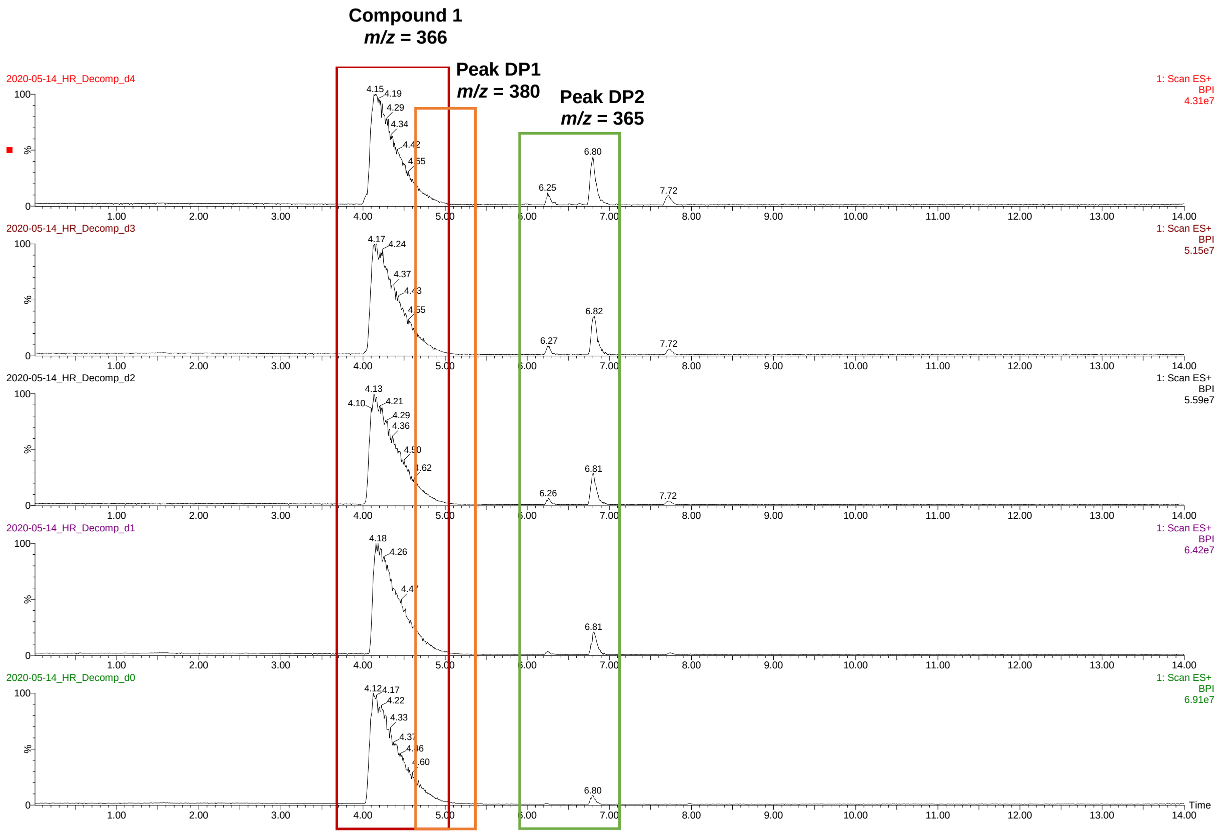

Figure S20. SFC chromatograms of the decomposition samples in DMSO over four days. The peak **DP1** 380 is co-eluting with the parent compound.

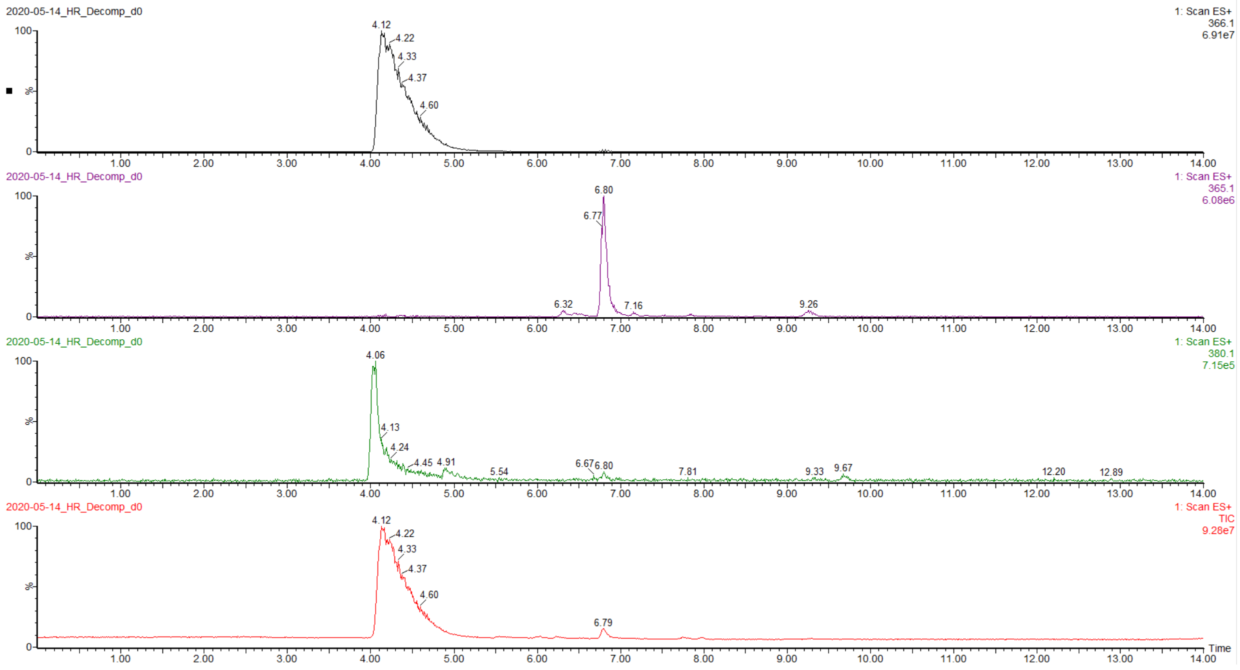

Figure S21a. SFC chromatograms of the decomposition samples in DMSO at d0. Black = EIC 366.1, purple = EIC 365.1, green = 380.1, red = TIC.

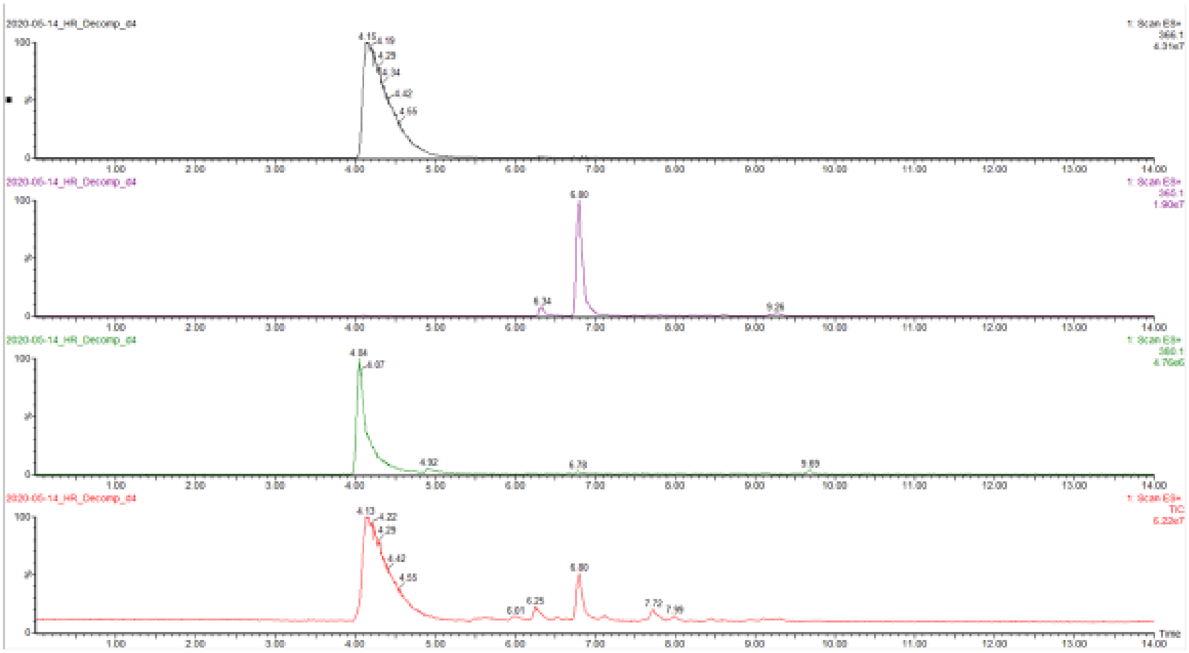

Figure S21b. SFC chromatograms of the decomposition samples in DMSO at d4. Black = EIC 366.1, purple = EIC 365.1, green = 380.1, red = TIC.

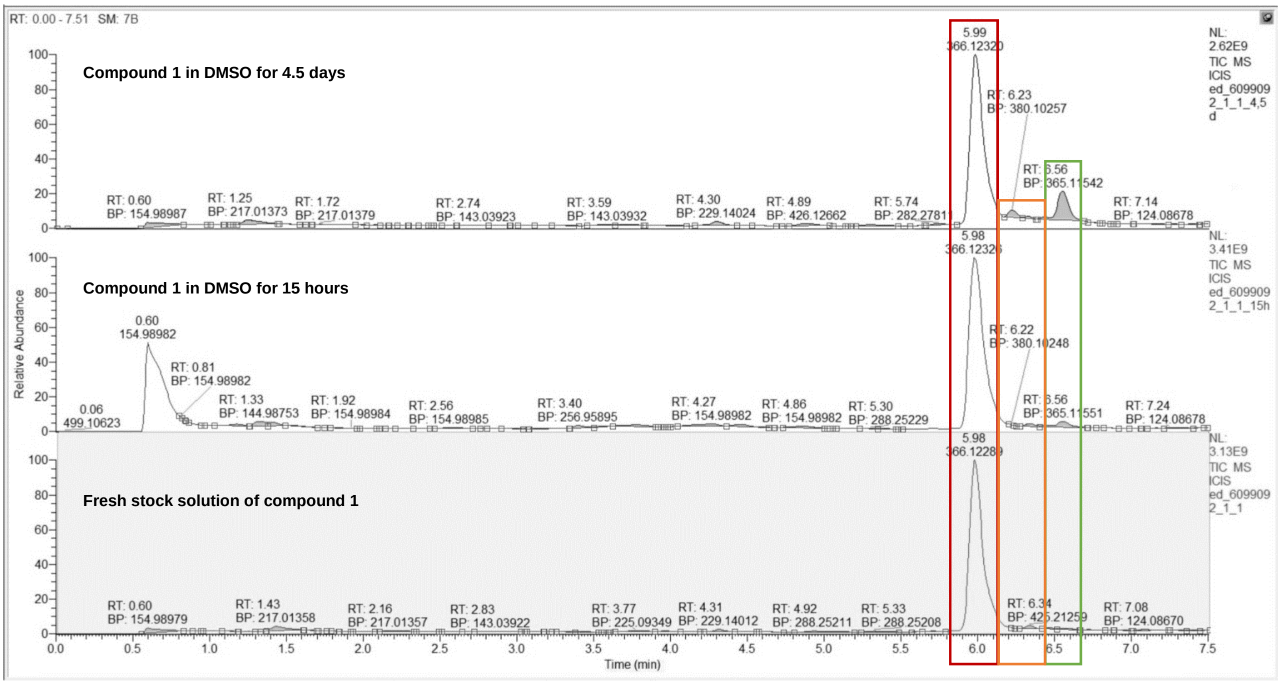

Figure S22. HRMS chromatograms of the decomposition samples in DMSO over to compare the visibility of the separation.

2.7 Characterisation of Decomposition Product 1

(*E*)-4-(1-ethyl-2,5-dimethyl-1*H*-pyrrol-3-yl)-2-((2-(trifluoromethyl)phenyl)imino)thiazol-5(2*H*)-one

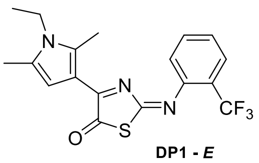

DP1 - *E*

Chemical Formula: $C_{18}H_{16}F_3N_3OS$
Exact Mass: 379.09662

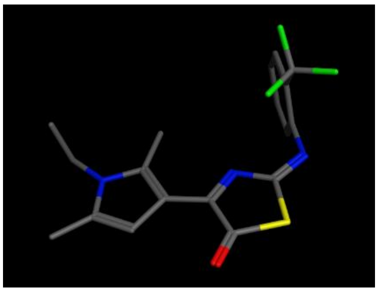

E = +29.4 kcal/mol of the lowest–energy conformation

(*Z*)-4-(1-ethyl-2,5-dimethyl-1*H*-pyrrol-3-yl)-2-((2-(trifluoromethyl)phenyl)imino)thiazol-5(2*H*)-one

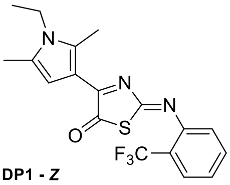

DP1 - *Z*

Chemical Formula: $C_{18}H_{16}F_3N_3OS$
Exact Mass: 379.09662

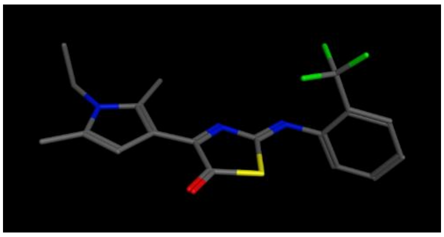

E = +33.2 kcal/mol of the lowest–energy conformation

(*E*)-1-ethyl-2,5-dimethyl-3-(5-oxo-2-((2-(trifluoromethyl)phenyl)amino)thiazol-4(5*H*)-ylidene)-3*H*-pyrrol-1-ium

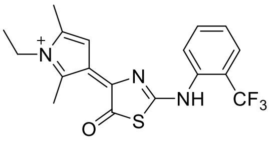

DP1 - *E*-pyrrolium

Chemical Formula: $C_{18}H_{17}F_3N_3OS^+$
Exact Mass: 380.10389

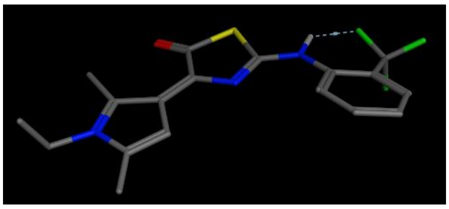

E = +66.1 kcal/mol of the lowest–energy conformation

(*Z*)-1-ethyl-2,5-dimethyl-3-(5-oxo-2-((2-(trifluoromethyl)phenyl)amino)thiazol-4(5*H*)-ylidene)-3*H*-pyrrol-1-ium

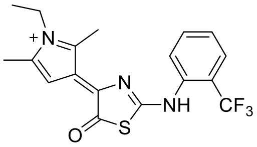

DP1 - *Z*-pyrrolium

Chemical Formula: $C_{18}H_{17}F_3N_3OS^+$
Exact Mass: 380.10389

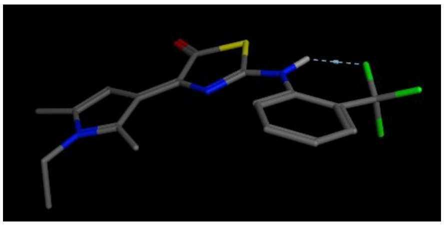

E = +62.8 kcal/mol of the lowest–energy conformation

(*MOE 2018.01 was used to calculate the lowest energy conformation in MMFF94X force field)*

DP1

Colour: bright red-orange

Isolated amount after SFC: 0.3 mg

HRMS (ESI+) calcd. for $C_{18}H_{16}F_3N_3OS$ [*M*+H]$^+$: 379.09662 or $C_{18}H_{17}F_3N_3OS^+$ [*M*]$^+$: 380.10389, found: 380.10376 for the mixture of compounds.

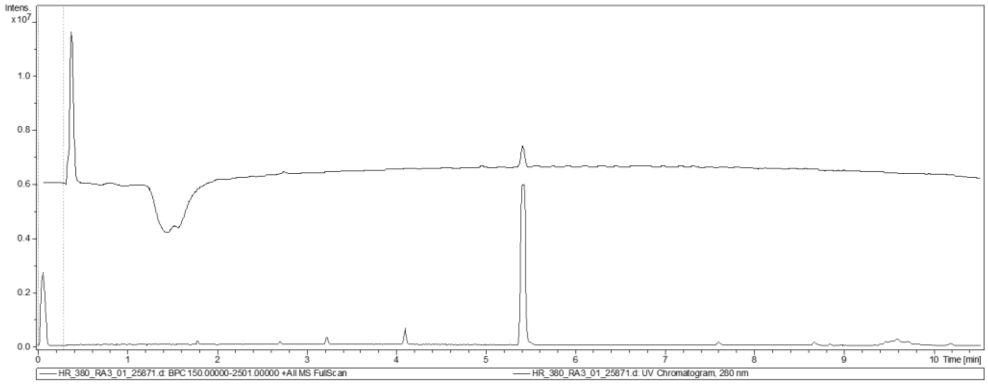

Figure S23. Chromatogram of the isolated **DP1**.

Table S4. Summary of the NMR shifts for **DP1** mixture.

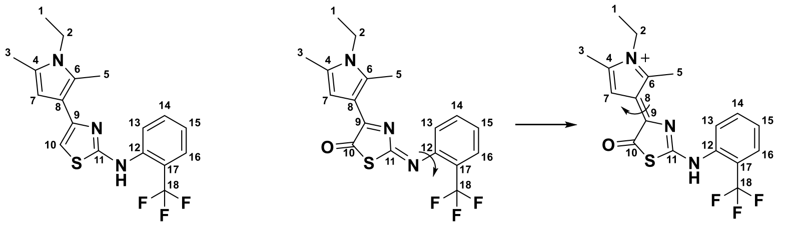

Exact Mass: 365.11735 | Exact Mass: 379.09662 | Exact Mass: 380.10389

#	Parent compound		Non-charged pyrrole		Charged pyrrolium	
	^{1}H 366	^{13}C 366	^{1}H 380	^{13}C 380	^{1}H 380	^{13}C 380
**1 – CH_3**	1.24	15.3	1.34	14.3	1.36	n.d.
**2 – CH_2**	3.93	37.9	4.09	38.7	4.09	38.7
**3 – CH_3**	2.24	11.3	2.31	11.1	2.32	n.d.
**4 – C**	x	127.4	x	133.4	x	n.d.
**5 – CH_3**	2.44	10.6	2.83	12.6	2.46	n.d.
**6 – C**	x	126.2	x	144.8	x	n.d.
**7 – CH**	6.23	105.1	6.87	108.8	6.99	108.9
**8 – C**	x	110.1	x	109.5	x	n.d.
**9 – C**	x	141.3	x	172.5	x	n.d.
**10 – C**	6.54	96.4	x	n.d.	x	n.d.
**11 – C**	x	168.7	x	n.d.	x	n.d.
**12 – C**	x	136.5	x	132.7	x	n.d.
**13 – CH**	7.88	127.2	7.78	130.0	7.13	121.5
**14 – CH**	7.58	127.4	7.61	132.2	7.70	133.4
**15 – CH**	7.83	134.2	7.86	133.4	7.41	125.1
**16 – CH**	8.13	126.2	7.93	127.0	7.73	126.2
**17 – C**	x	124.1	x	n.d.	x	n.d.
**18 – CF_3**	x	122.2	x	n.d.	x	n.d.

- Measured in acetone-d_6 n.d.: not determined

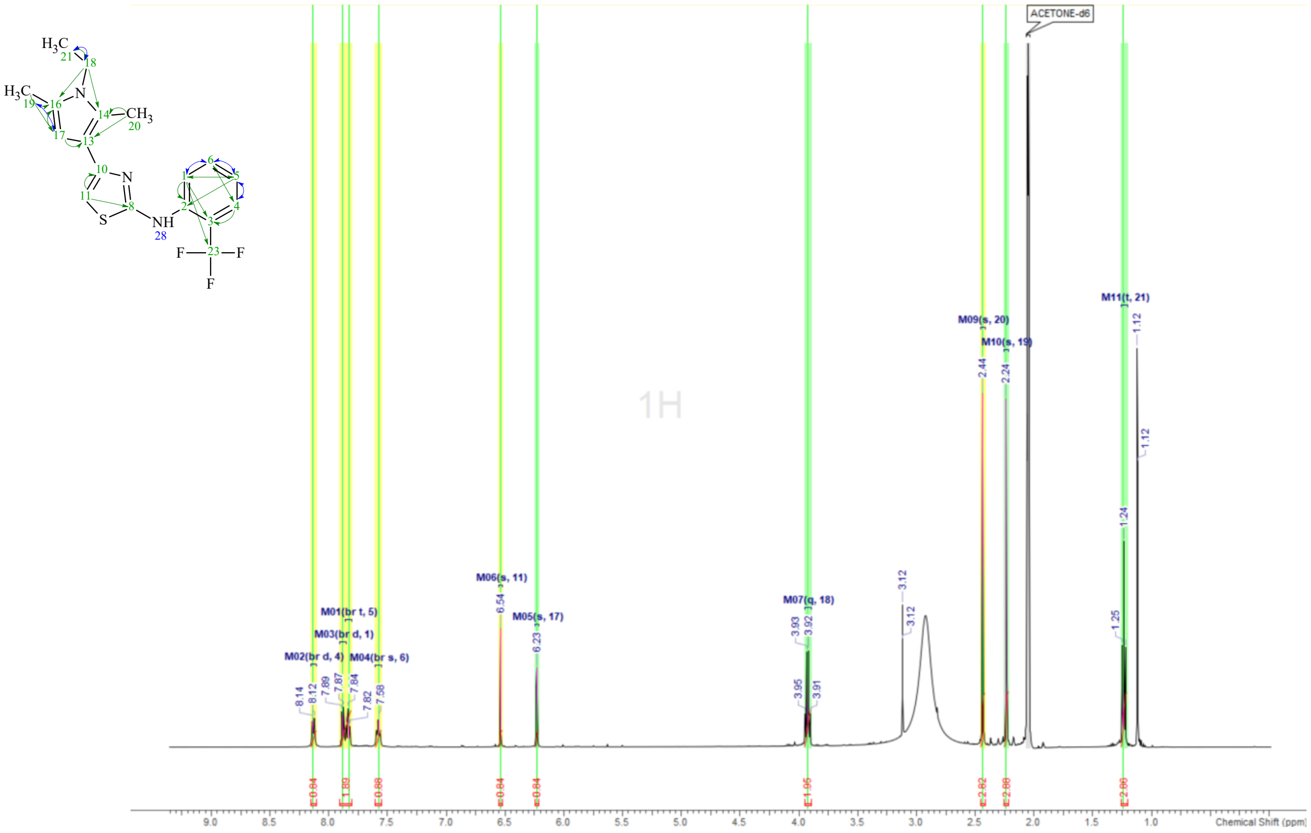

Figure S24. ^{1}H-NMR spectrum of compound **1** in acetone-d_6.

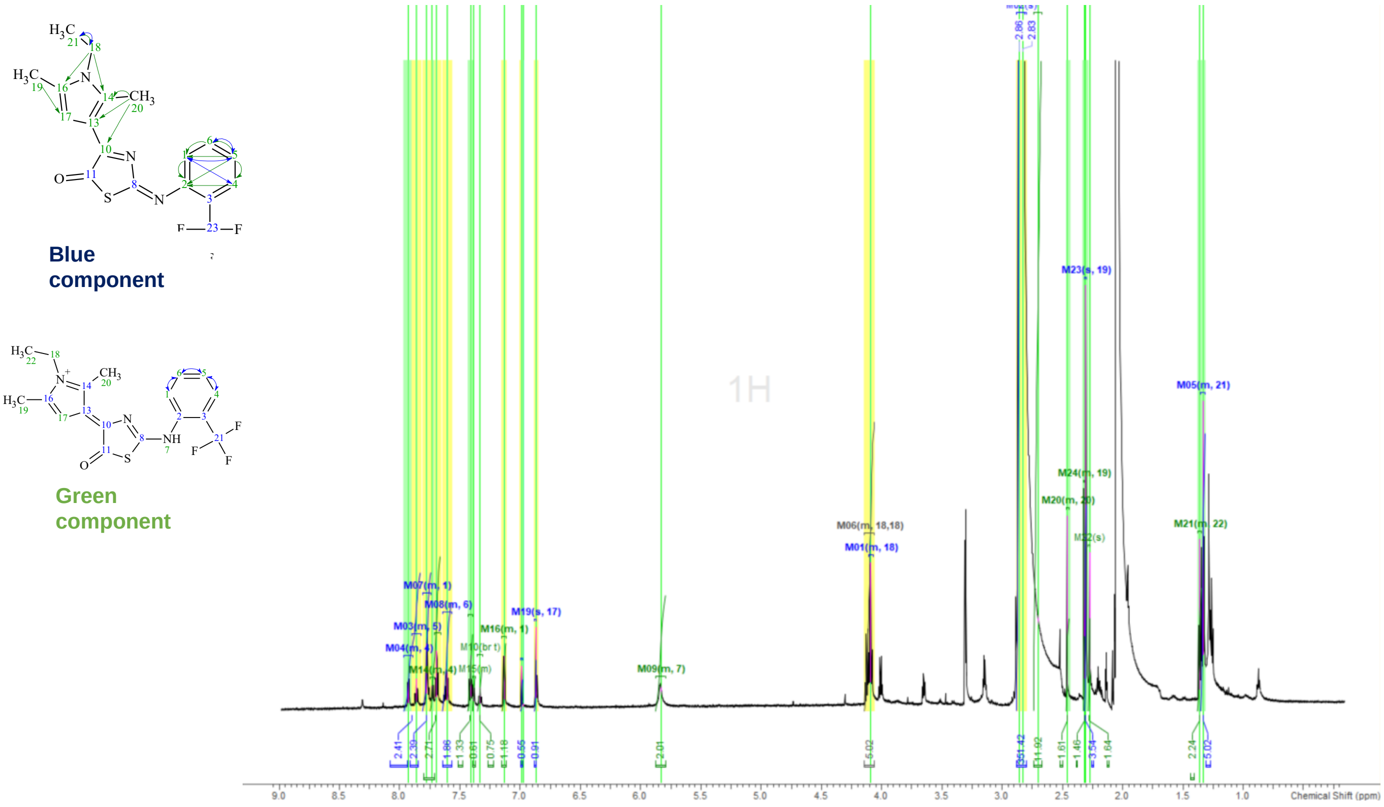

Figure S25. ^{1}H-NMR spectrum of **DP1** mixture in acetone-d_6.

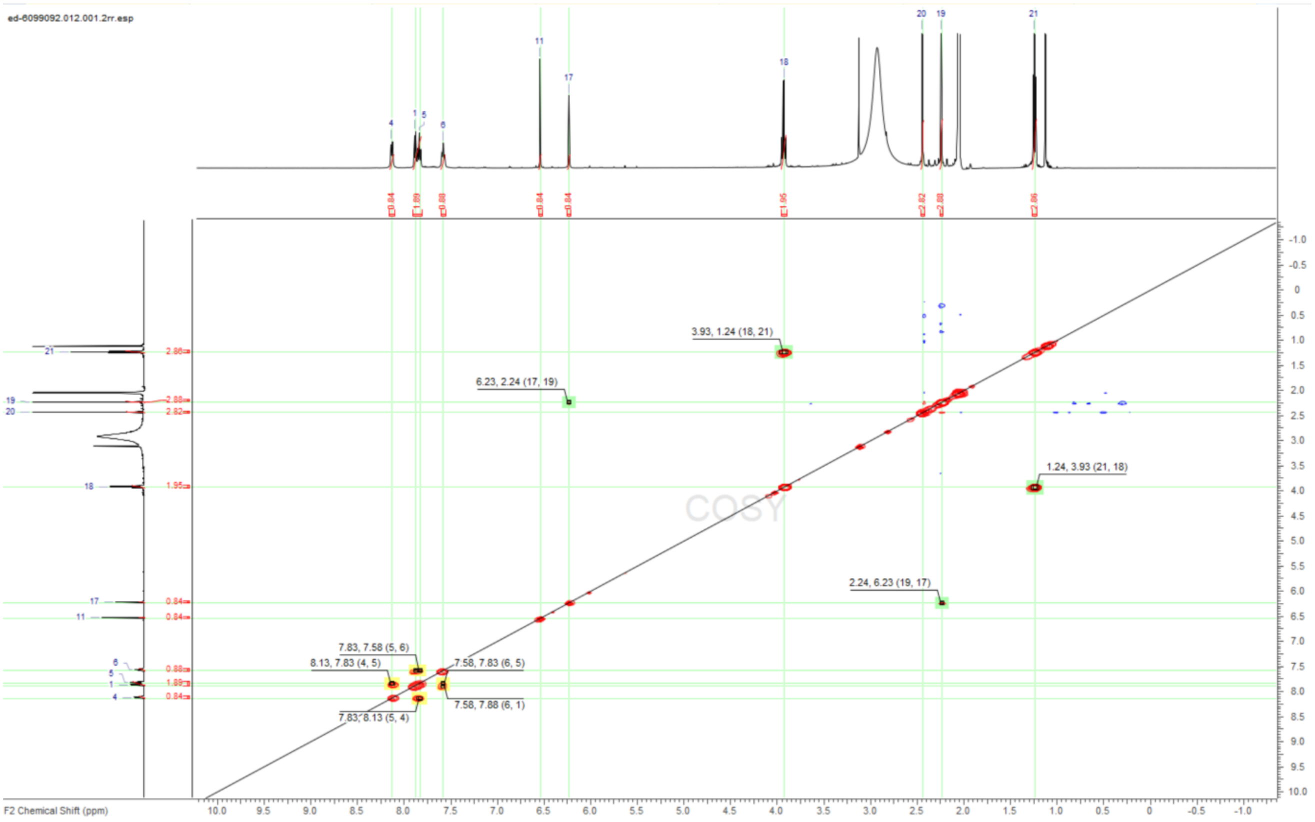

Figure S26. [^{1}H,^{1}H]-COSY NMR spectrum of compound **1** in acetone-d_6.

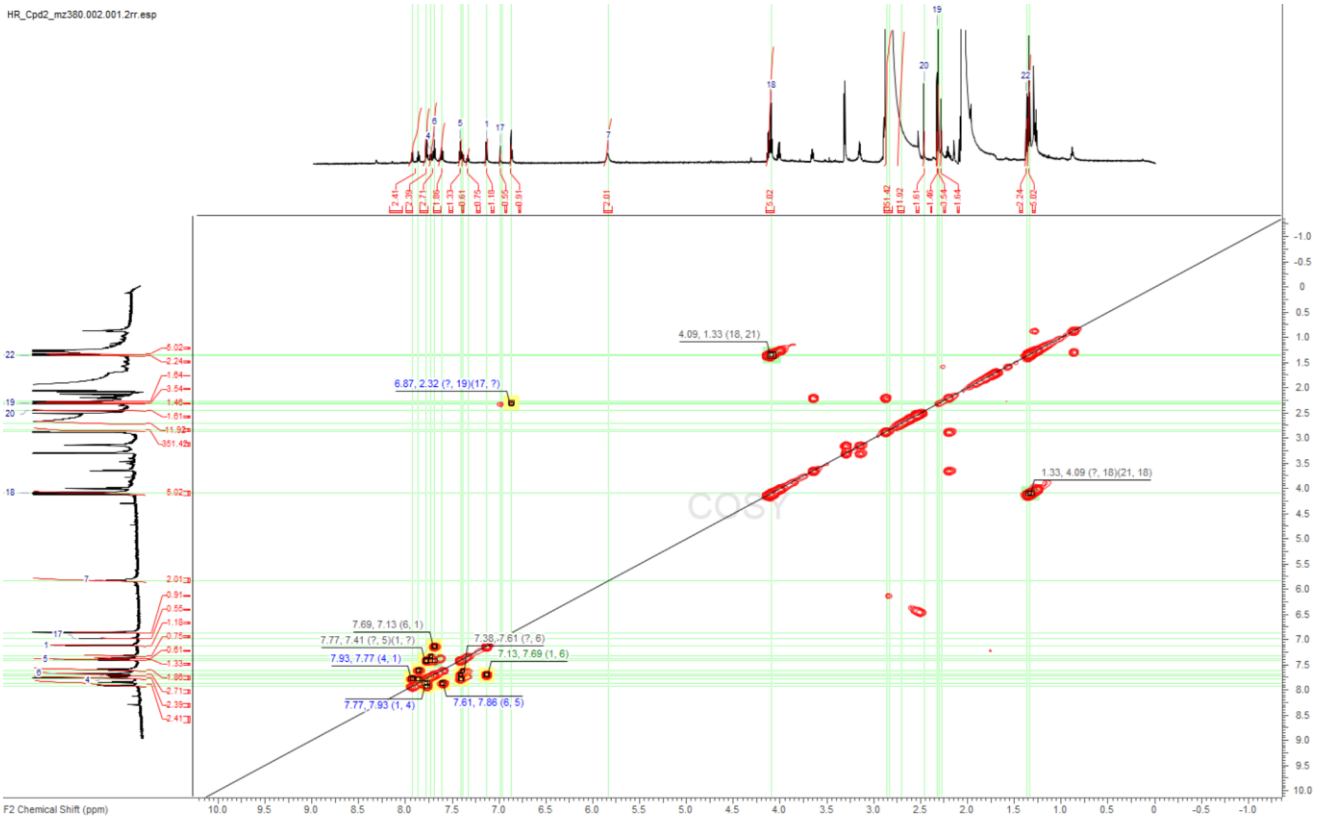

Figure S27. [^{1}H,^{1}H]-COSY NMR spectrum of **DP1** mixture in acetone-d_6.

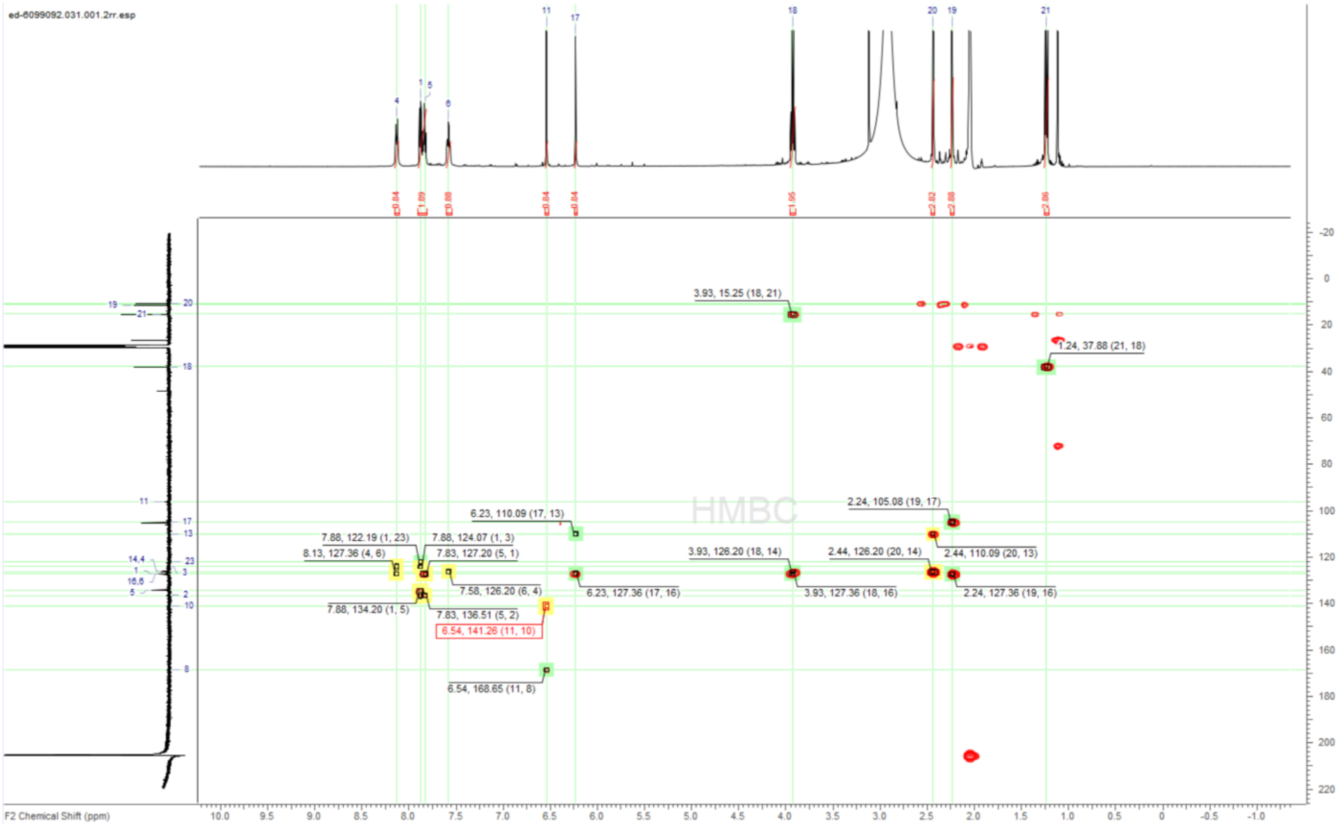

Figure S28. [^{1}H,^{13}C]-HMBC spectrum of compound **1** in acetone-*d*$_6$.

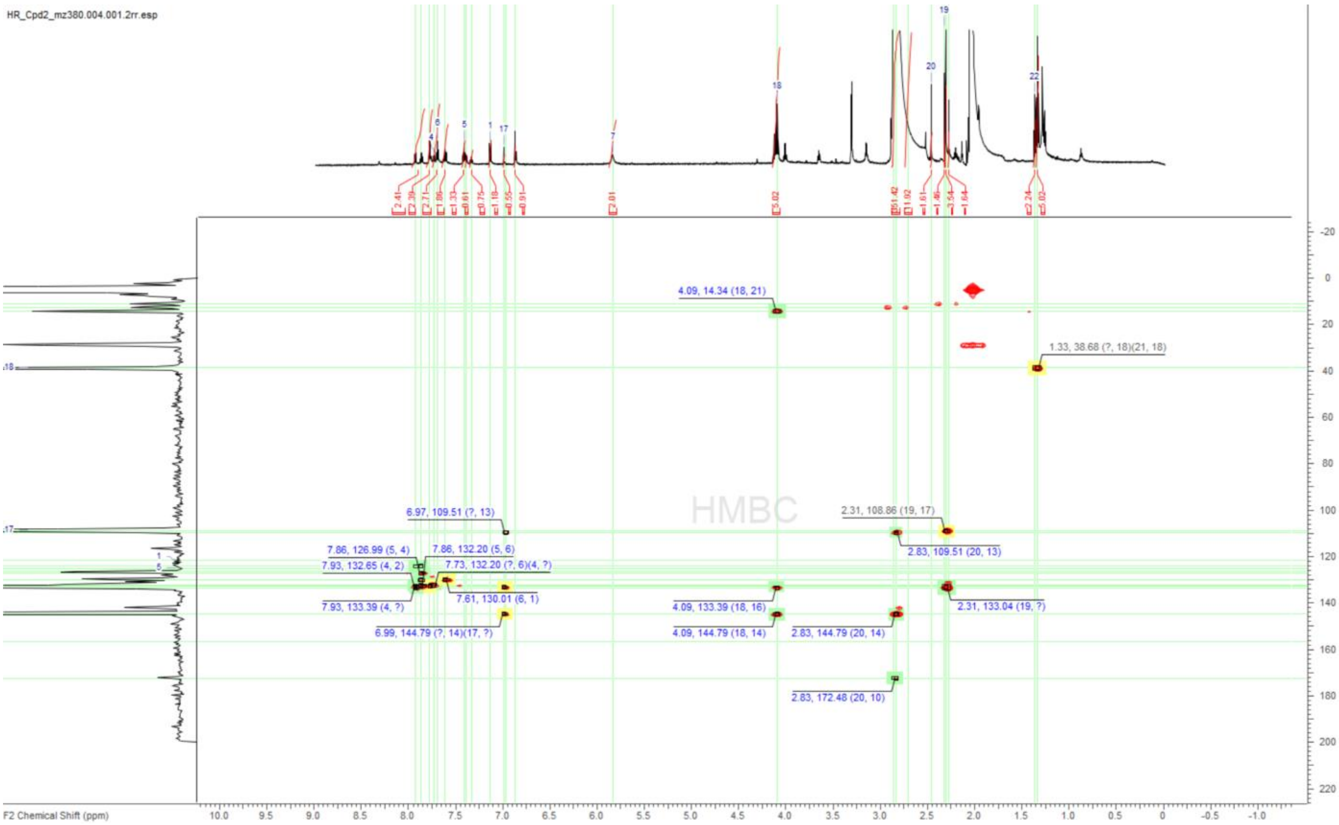

Figure S29. [^{1}H,^{13}C]-HMBC spectrum of **DP1** mixture in acetone-*d*$_6$.

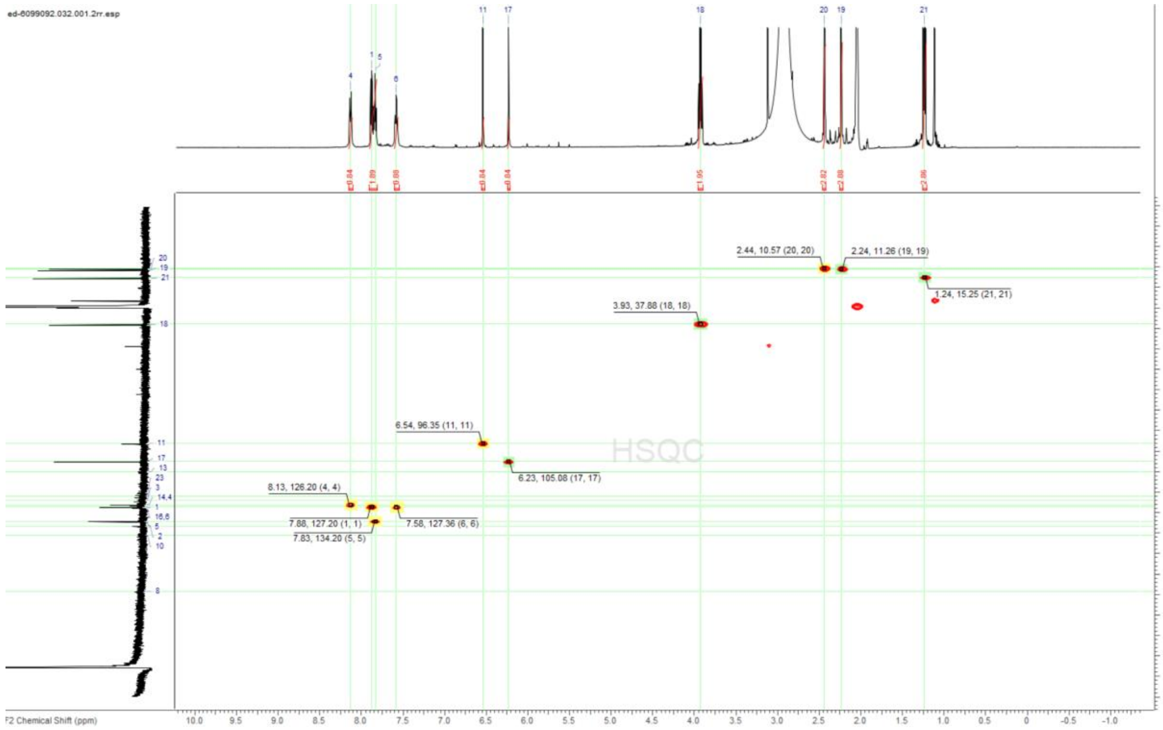

Figure S30. [^{1}H,^{13}C]-HSQC spectrum of compound **1** in acetone-d_6.

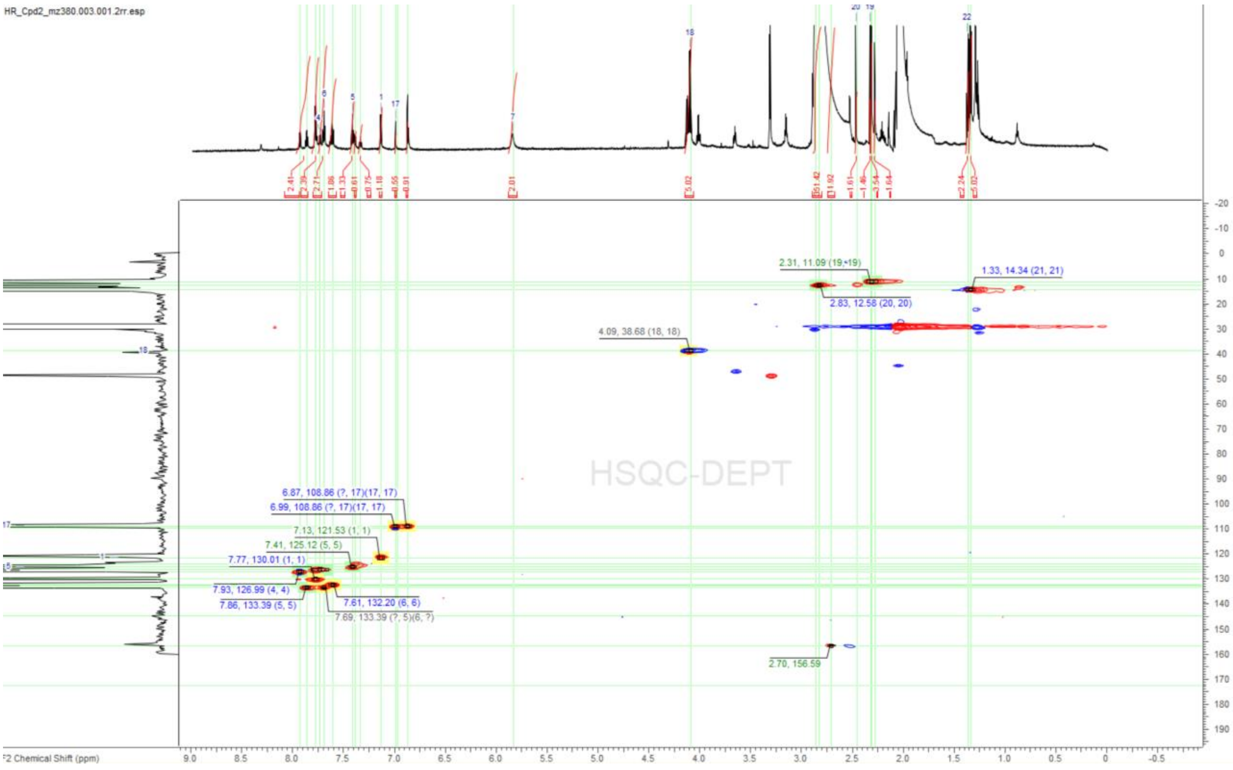

Figure S31. [^{1}H,^{13}C]-HSQC-DEPT spectrum of **DP1** mixture in acetone-d_6.

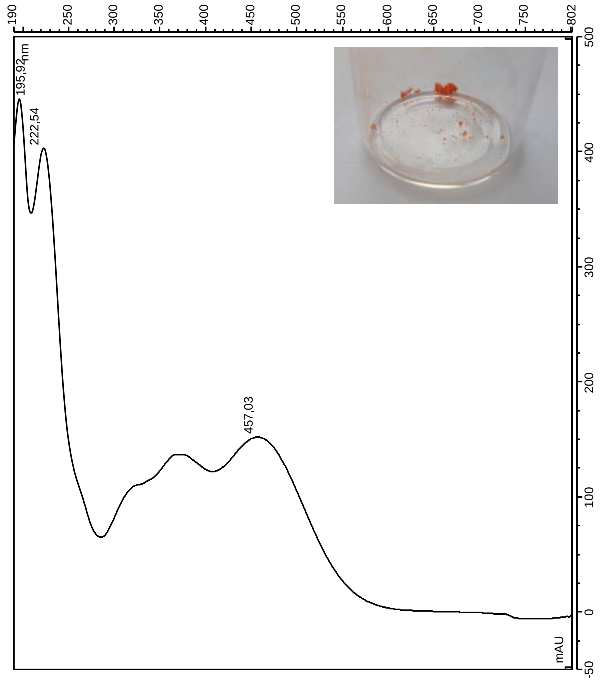

Figure S32. UV-spectra of **DP1** mixture with the distinct colour.

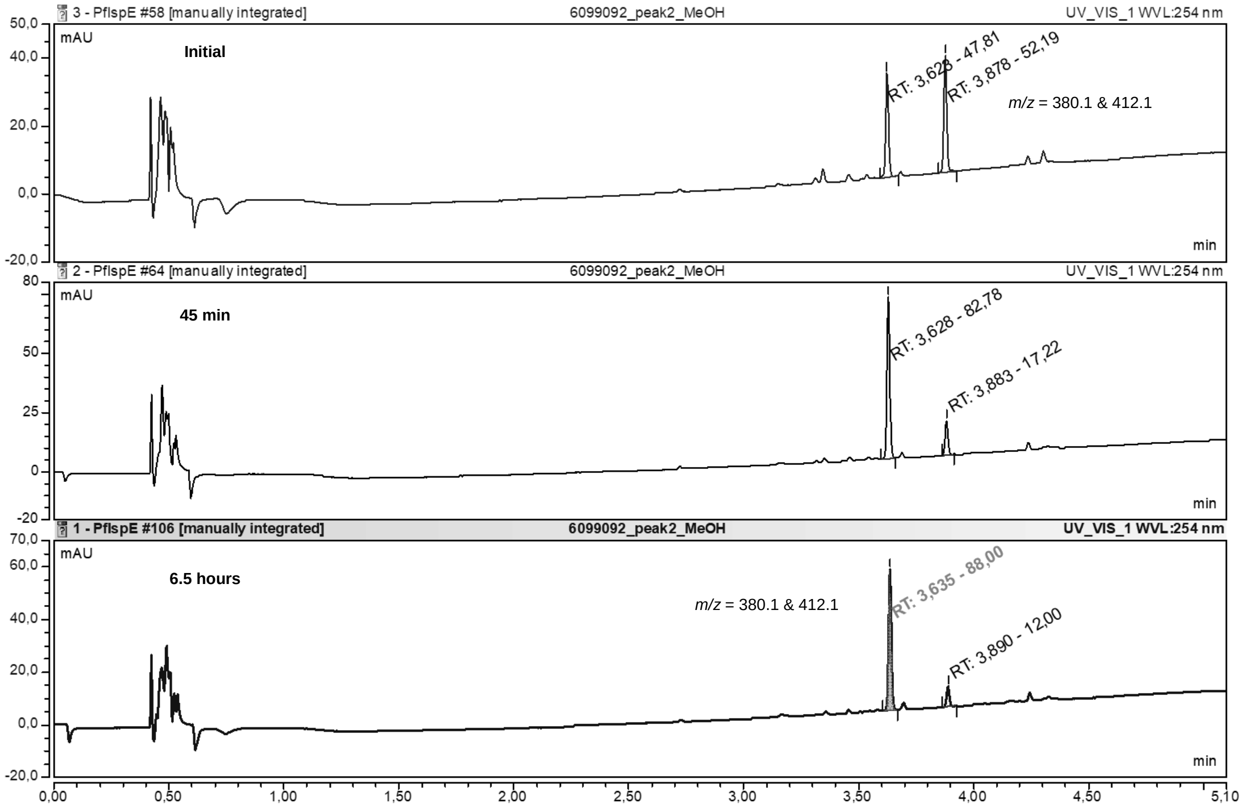

Figure S33. Stability of **DP1** in MeOH and the observed shift in retention time.

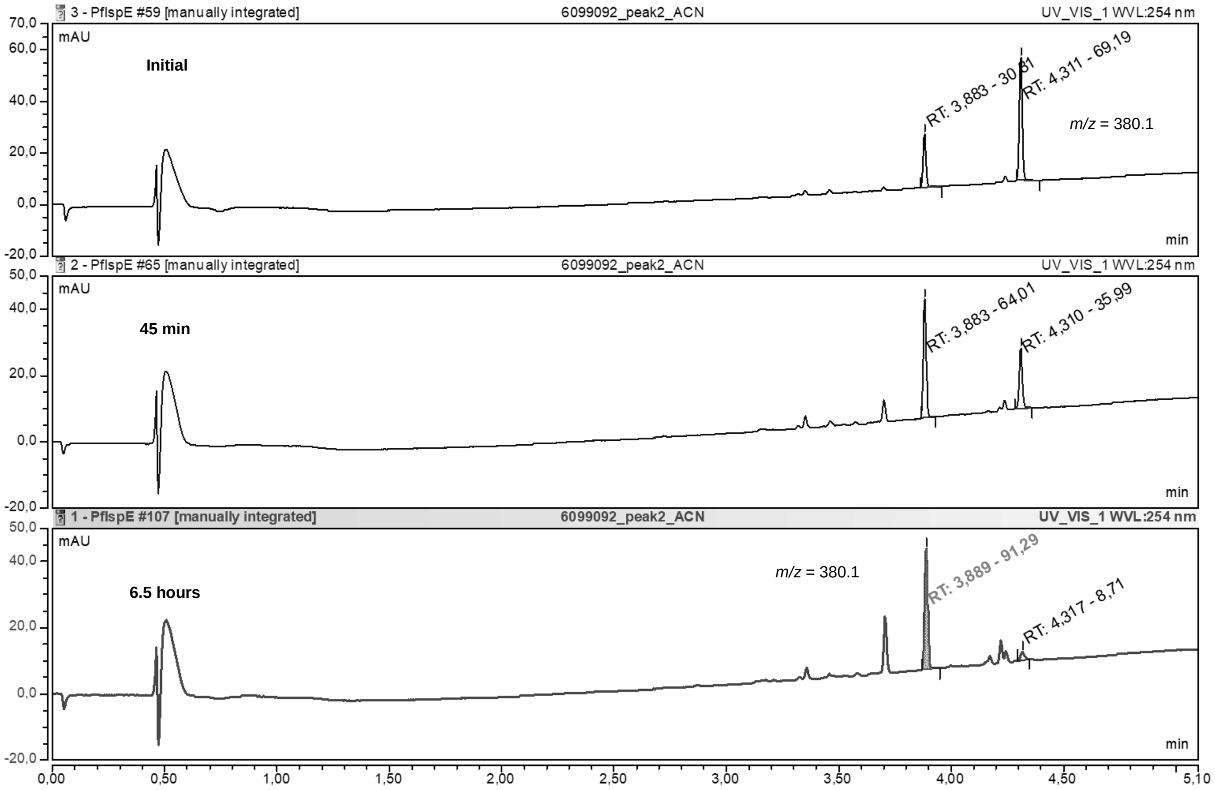

Figure S34. Stability of **DP1** in ACN and the observed shift in retention time.

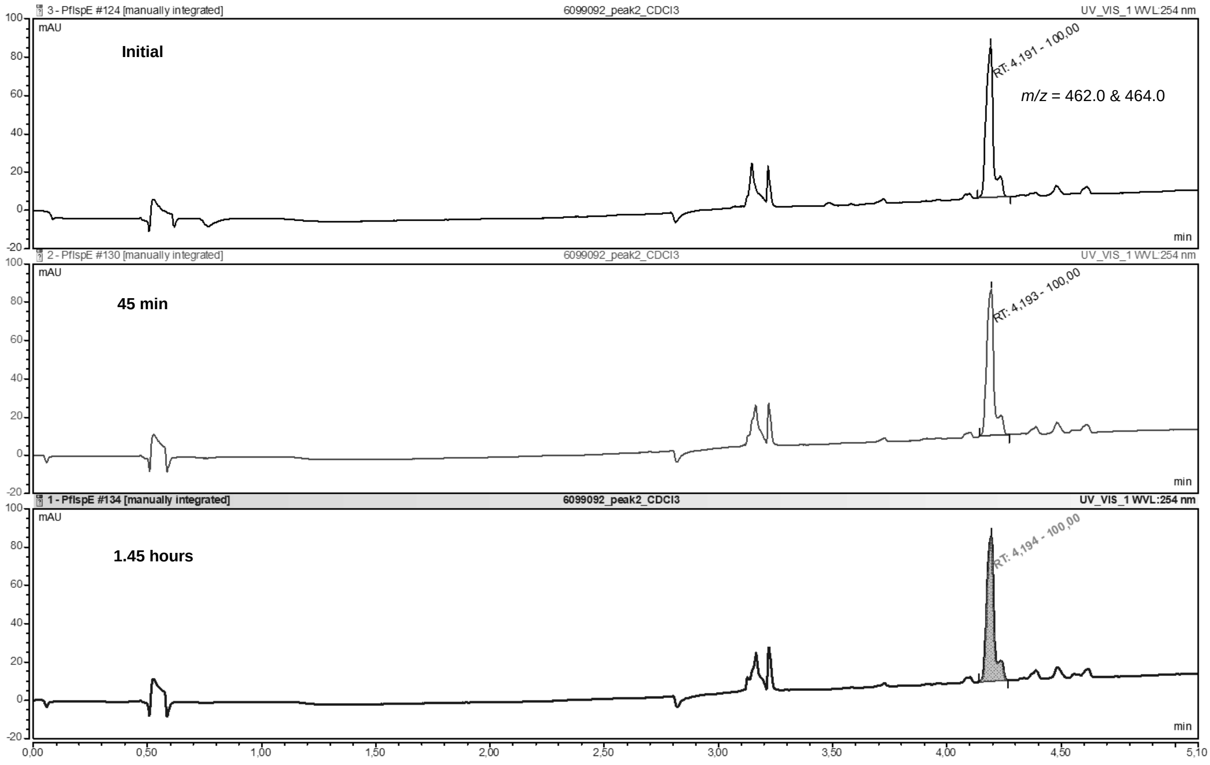

Figure S35. Stability of **DP1** in $CDCl_3$.

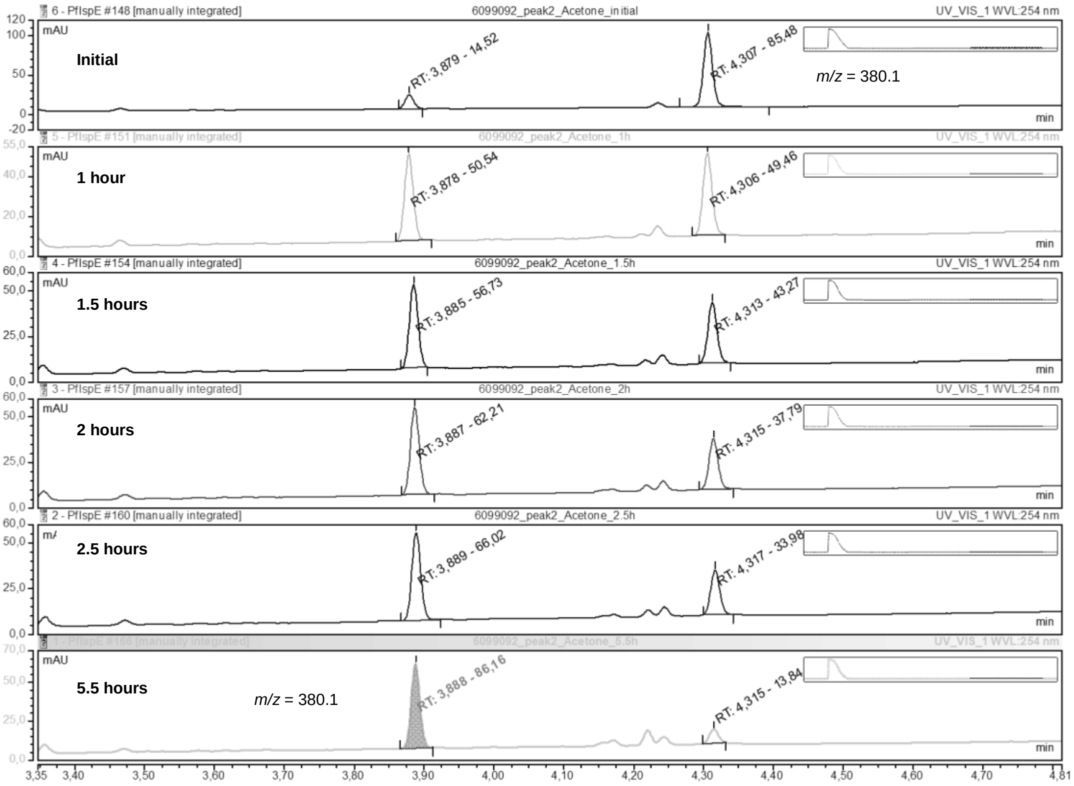

Figure S36. Stability of **DP1** in acetone and the observed shift in retention time.

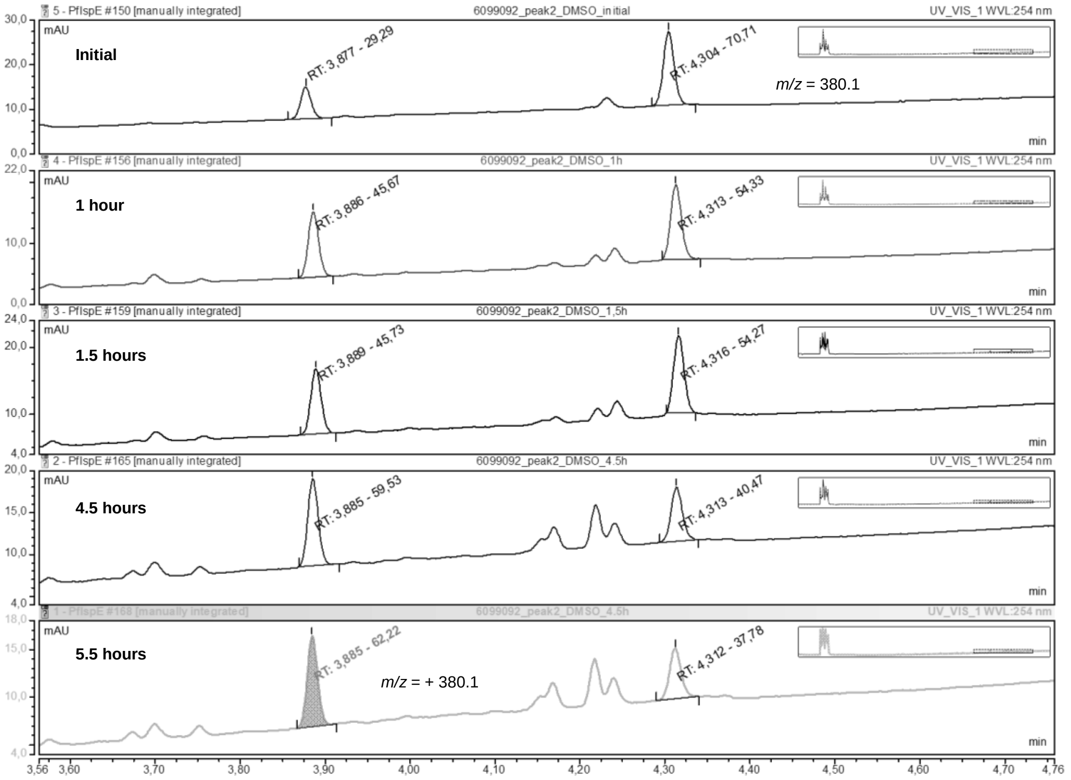

Figure S37. Stability of **DP1** in DMSO and the observed shift in retention time.

2.8 Characterisation of Decomposition Product 2

(*Z*)-1-ethyl-3-(2-(2-(4-(1-ethyl-2,5-dimethyl-1*H*-pyrrol-3-yl)thiazol-2-yl)-1,2-bis(2-(trifluoromethyl)phenyl)hydrazineyl)thiazol-4(5*H*)-ylidene)-2,5-dimethyl-3*H*-pyrrol-1-ium

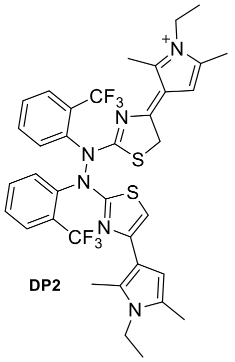

DP2

Exact Mass: 729.22633

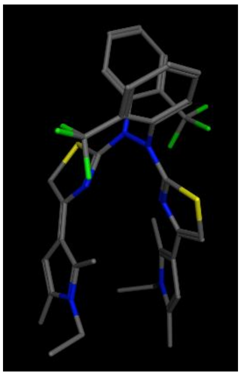

E = +144.7 kcal/mol of the lowest–energy conformation

(*E*)-1-ethyl-3-(2-(2-(4-(1-ethyl-2,5-dimethyl-1*H*-pyrrol-3-yl)thiazol-2-yl)-1,2-bis(2-(trifluoromethyl)phenyl)hydrazineyl)thiazol-4(5*H*)-ylidene)-2,5-dimethyl-3*H*-pyrrol-1-ium

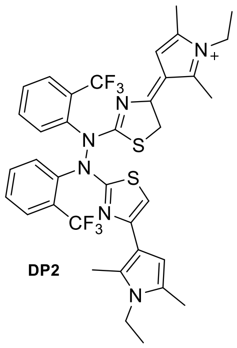

DP2

Exact Mass: 729.22633

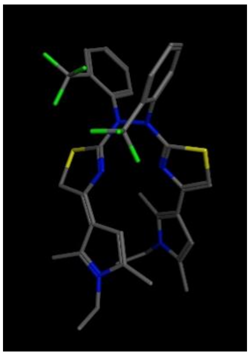

E = +148.5 kcal/mol of the lowest–energy conformation

(*MOE 2018.01 was used to calculate the lowest energy conformation in MMFF94X force field)*

DP2

Colour: beige brown powder

Isolated amount after SFC: 0.9 mg

HRMS (ESI+) calcd. for $C_{36}H_{35}F_6N_6S_2^+$ [*M*]$^+$: 729.22633, found: 729.22473; and [*M*+2H]$^{2+}$: 365.11681, found: 365.11642.

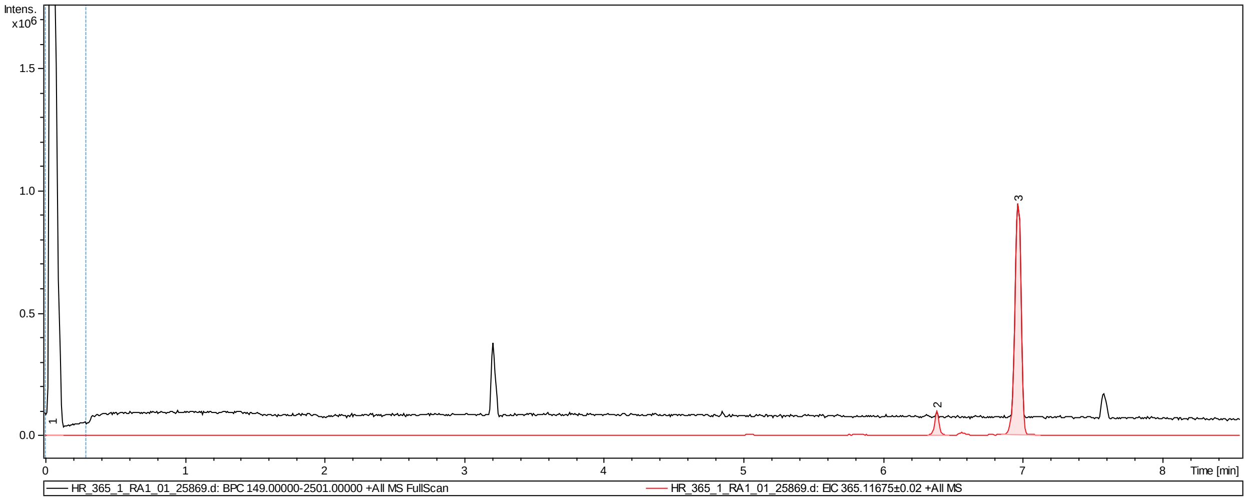

Figure S38. Chromatogram of the isolated **DP2**. Red = EIC at 365.11 ± 0.02, Black = BPC.

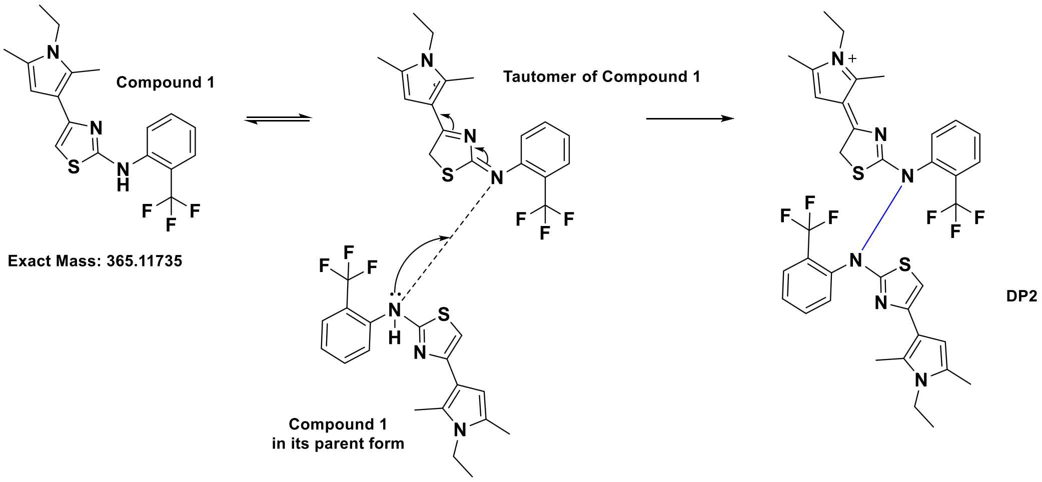

Scheme S1. Proposed reaction mechanism for the formation of **DP2.**

Table S5. Summary of the **DP2** NMR shifts.

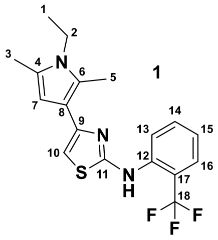

1

Exact Mass: 365.11735

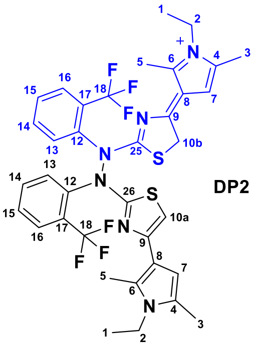

DP2

Exact Mass: 729.22633

	Parent compound		Dimer	
#	^{1}H 366	^{13}C 366	^{1}H DP2[a]	^{13}C DP2
**1 – CH_3**	1.22	16.5	1.23	16.1
	-	-	b - 1.01	b - 16.1
**2 – CH_2**	3.88	39.4	3.87	38.9
	-	-	b - 3.68	b - 38.9
**3 – CH_3**	2.23	12.5	2.22	11.9
	-	-	b - 2.15	b – 11.4
**4 – C**	x	128.7	x	127.4
	-	-	b - x	b – 129.1
**5 – CH_3**	2.38	11.9	2.27	11.4
	-	-	b - 2.42	b – 11.4
**6 – C**	x	127.4	x	126.6
	-	-	b - x	b – 175.6
**7 – CH**	6.14	106.3	5.87	107.2
	-	-	b - 6.09	b - 106.5
**8 – C**	x	110.6	x	114.1
	-	-	b - x	b – 131.2
**9 – C**	x	141.5	x	149.5
	-	-	b - x	b - 121.1
**10a – C**	6.36	98.3	6.48	100.98
**10b – CH_2**	-	-	b - 3.96	b - 25.4
**11/26 – C**	x	169.5	x	164.2
**(11)25 – C**	-	-	b - x	b - 143.8
**12 – C**	x	137.6	x	140.1
	-	-	b - x	b - 140.8
**13 – CH**	7.83	128.7	7.68	127.4
	-	-	b - 7.64	b - 127.3
**14 – CH**	7.51	128.9	7.22	123.9
	-	-	b - 7.17	b - 123.8
**15 – CH**	7.74	135.6	7.60	134.2
	-	-	b - 7.56	b - 134.1
**16 – CH**	7.91	127.4	8.20	123.9
	-	-	b - 8.09	b - 123.6
**17 – C**	x	125.9	x	x[b]
	-	-	b - n.d.[b]	b - 126.01
**18 – CF_3**	x	123.7	x	x
	-	-	b - n.d.[b]	b - 124.5 [b]

- Measured in acetonitrile-d_3, n.d.: not determined [a]: Peaks “b” corresponding to the blue part of the dimer [b]: Overlapping with the other dimer part.

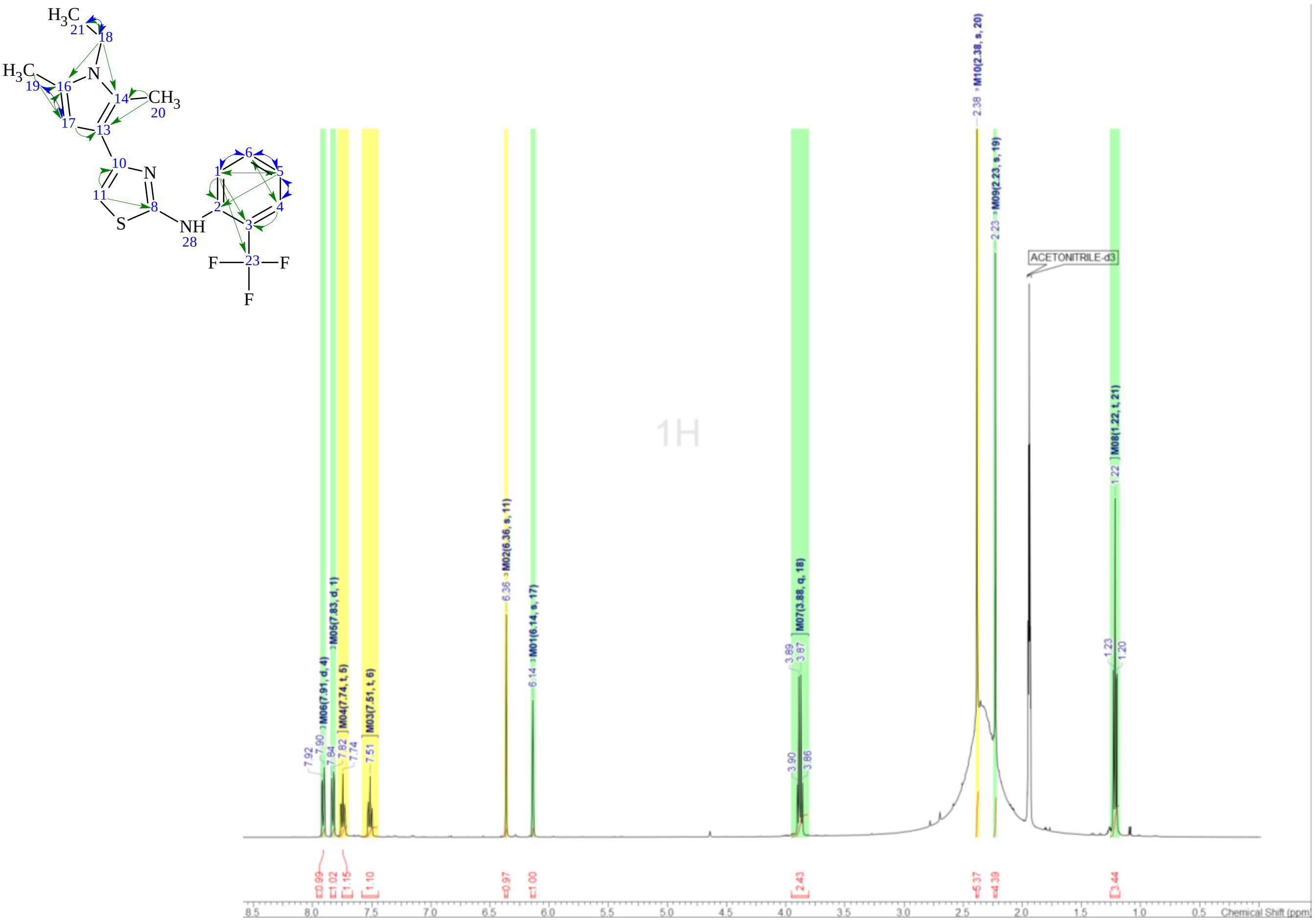

Figure S39. ^{1}H-NMR spectrum of compound **1** in acetonitrile-d_3.

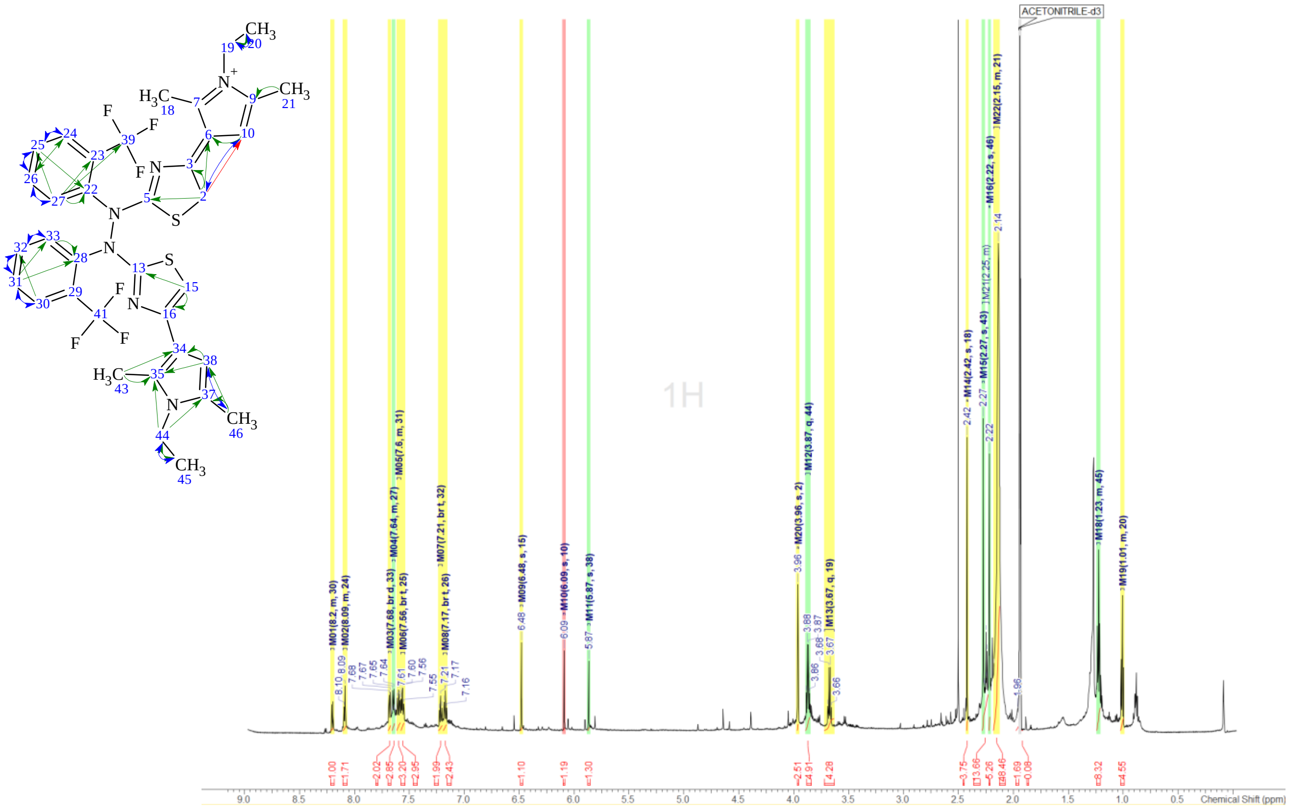

Figure S40. ^{1}H-NMR spectrum of **DP2** in acetonitrile-d_3.

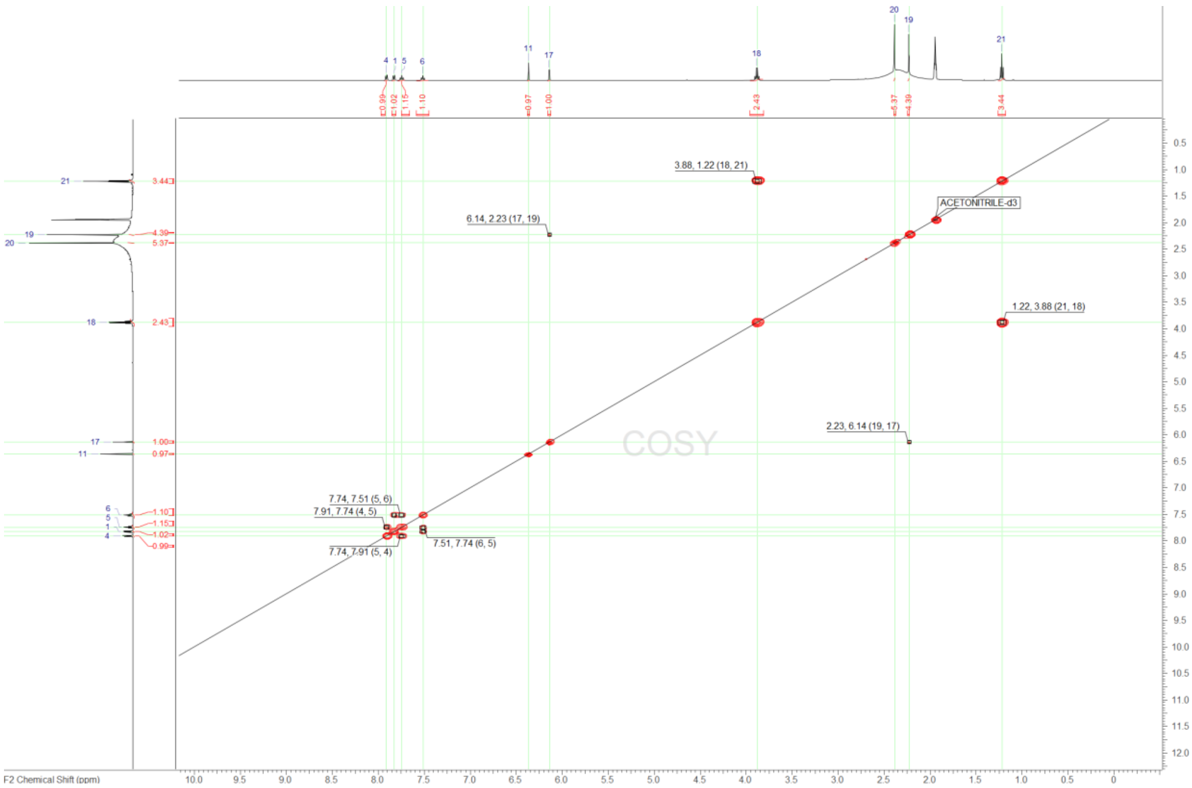

Figure S41. [^{1}H,^{1}H]-COSY NMR spectrum of compound **1** in acetonitrile-d_3.

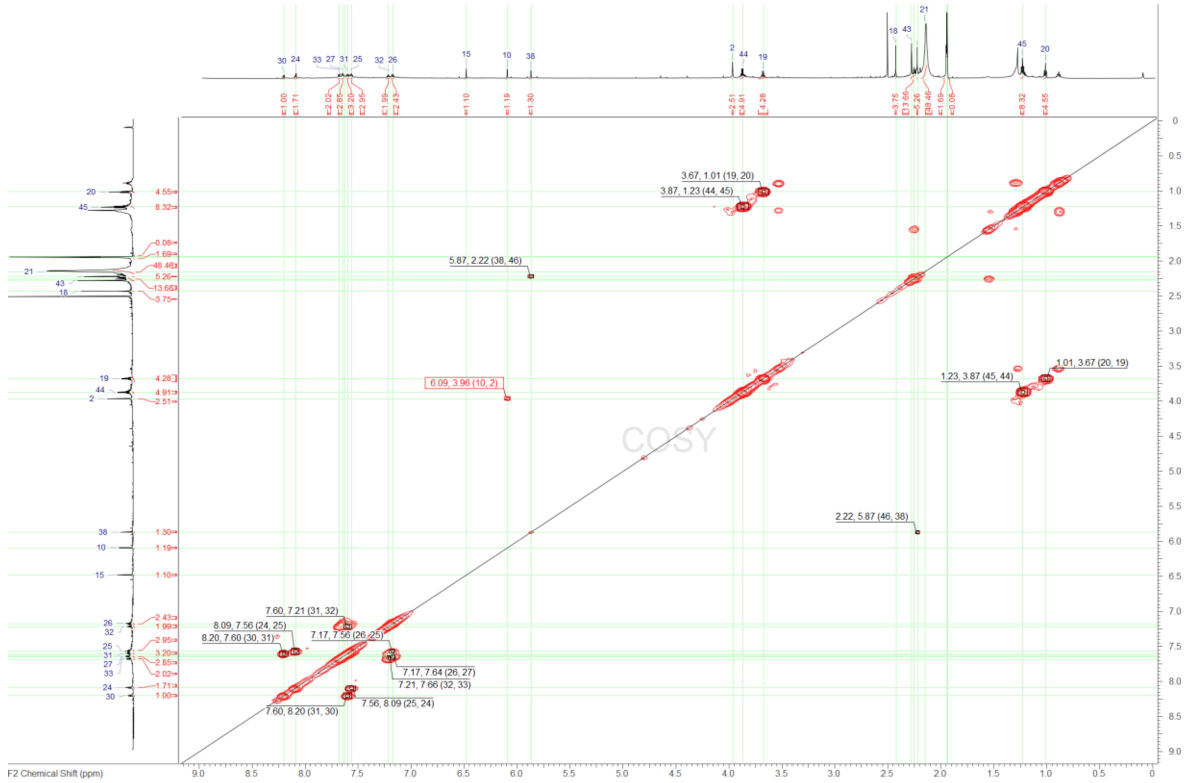

Figure S42. [^{1}H,^{1}H]-COSY NMR spectrum of **DP2** in acetonitrile-d_3.

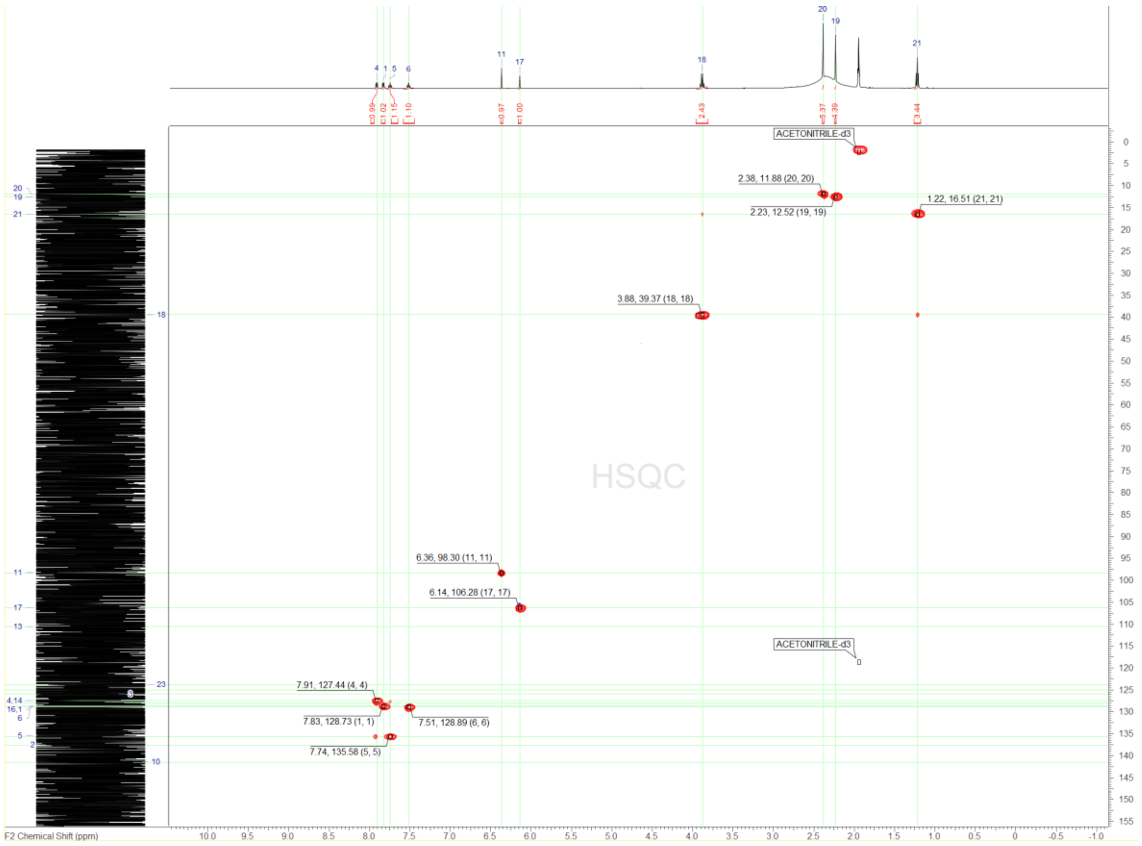

Figure S43. [^{1}H,^{13}C]-HSQC spectrum of compound **1** in acetonitrile-*d*$_3$.

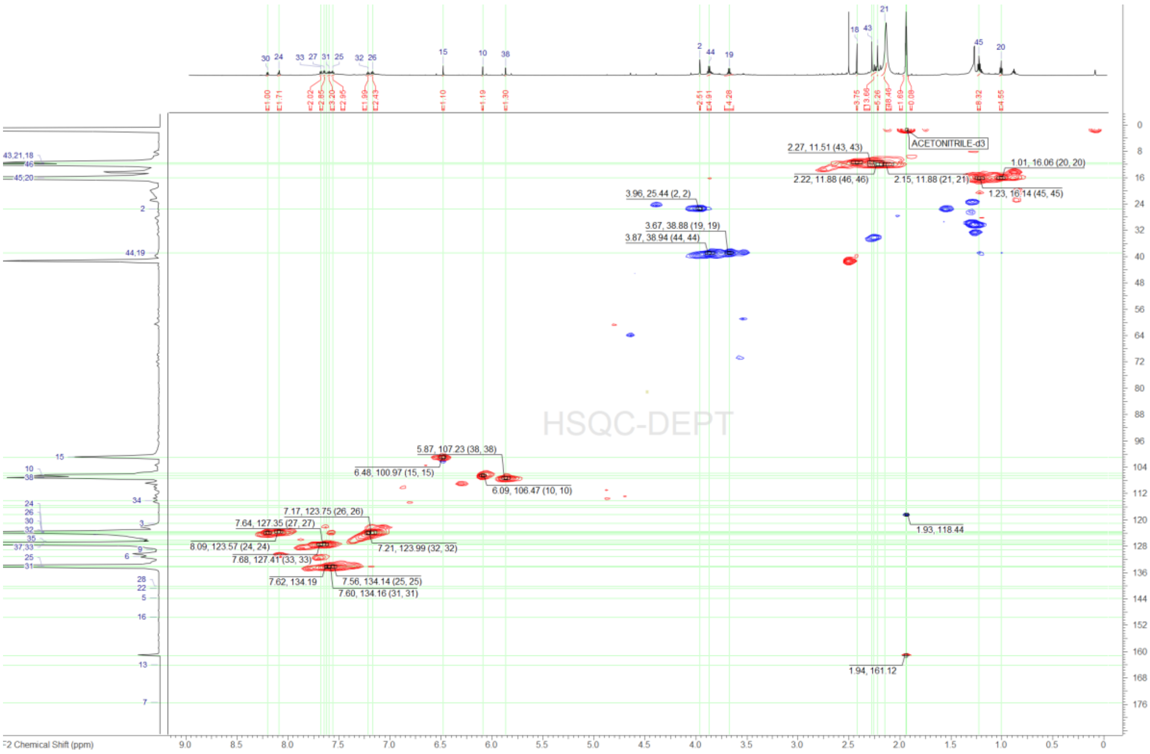

Figure S44. [^{1}H,^{13}C]-HSQC-DEPT spectrum of **DP2** in acetonitrile-*d*$_3$.

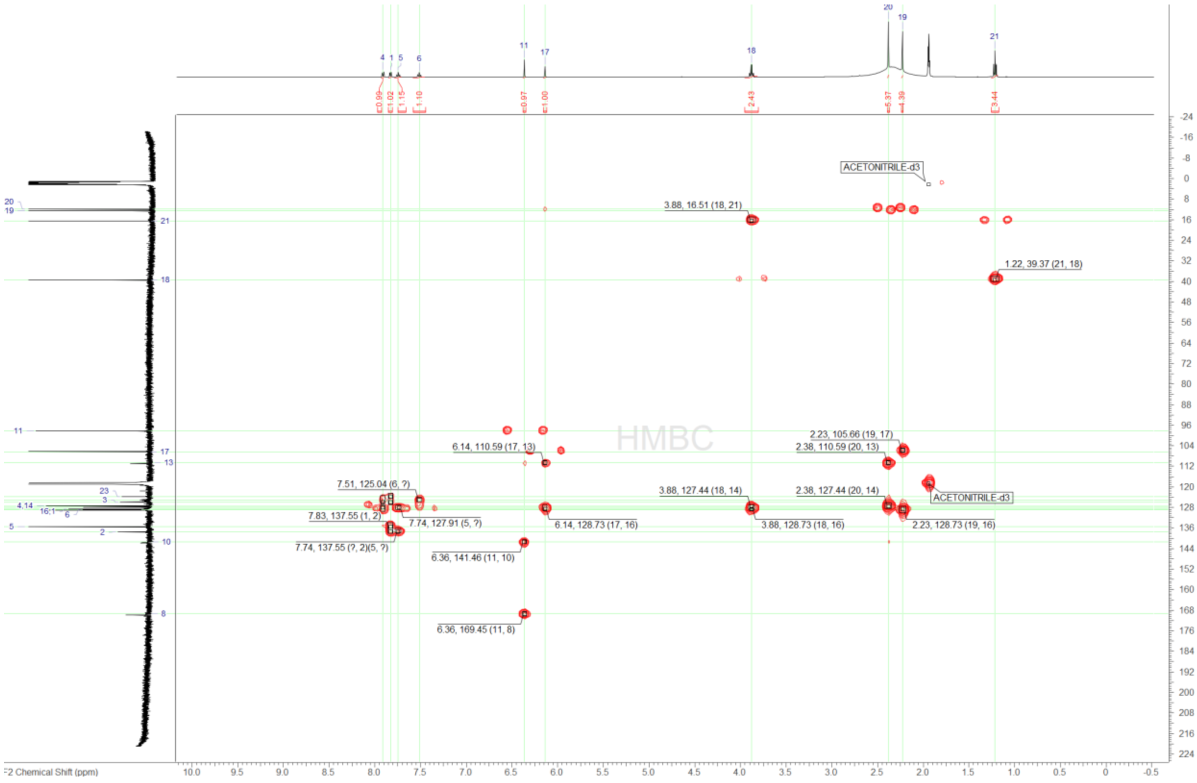

Figure S45. [^{1}H,^{13}C]-HMBC spectrum of compound **1** in acetonitrile-d_3.

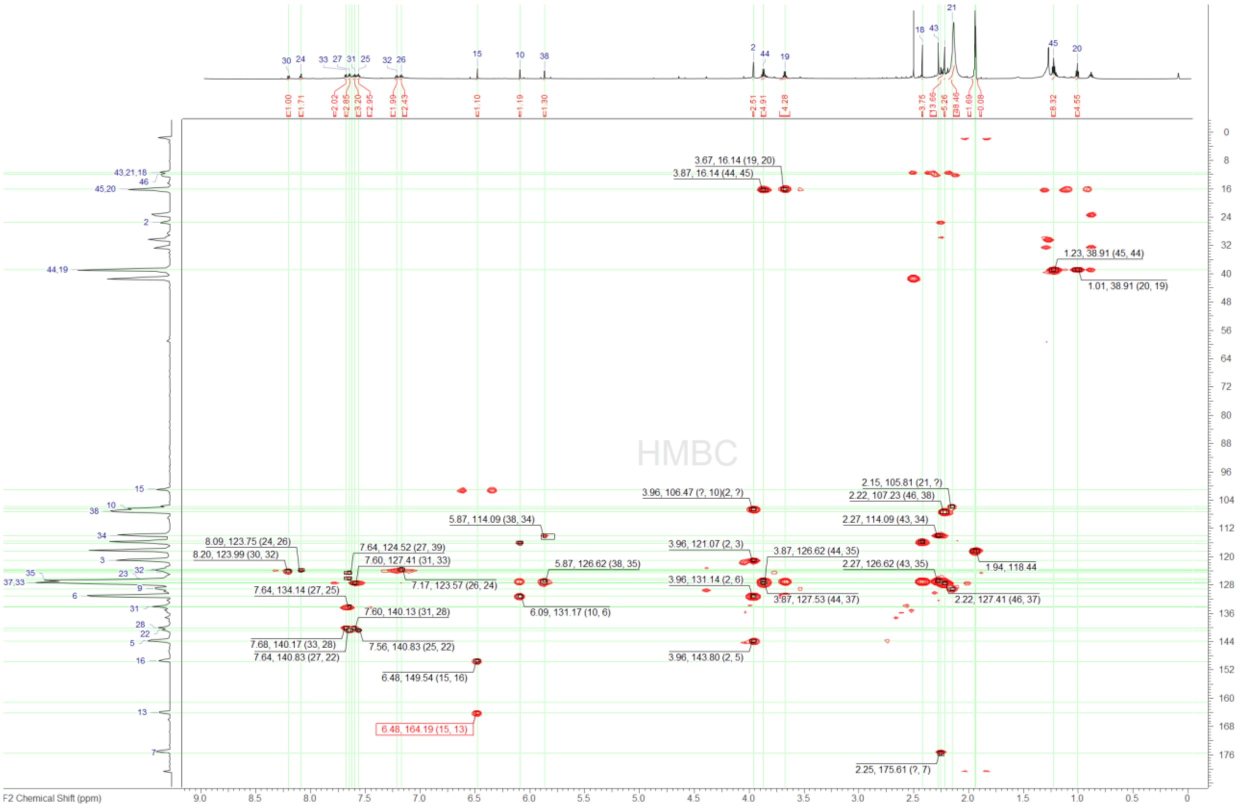

Figure S46. [^{1}H,^{13}C]-HMBC spectrum of **DP2** in acetonitrile-d_3.

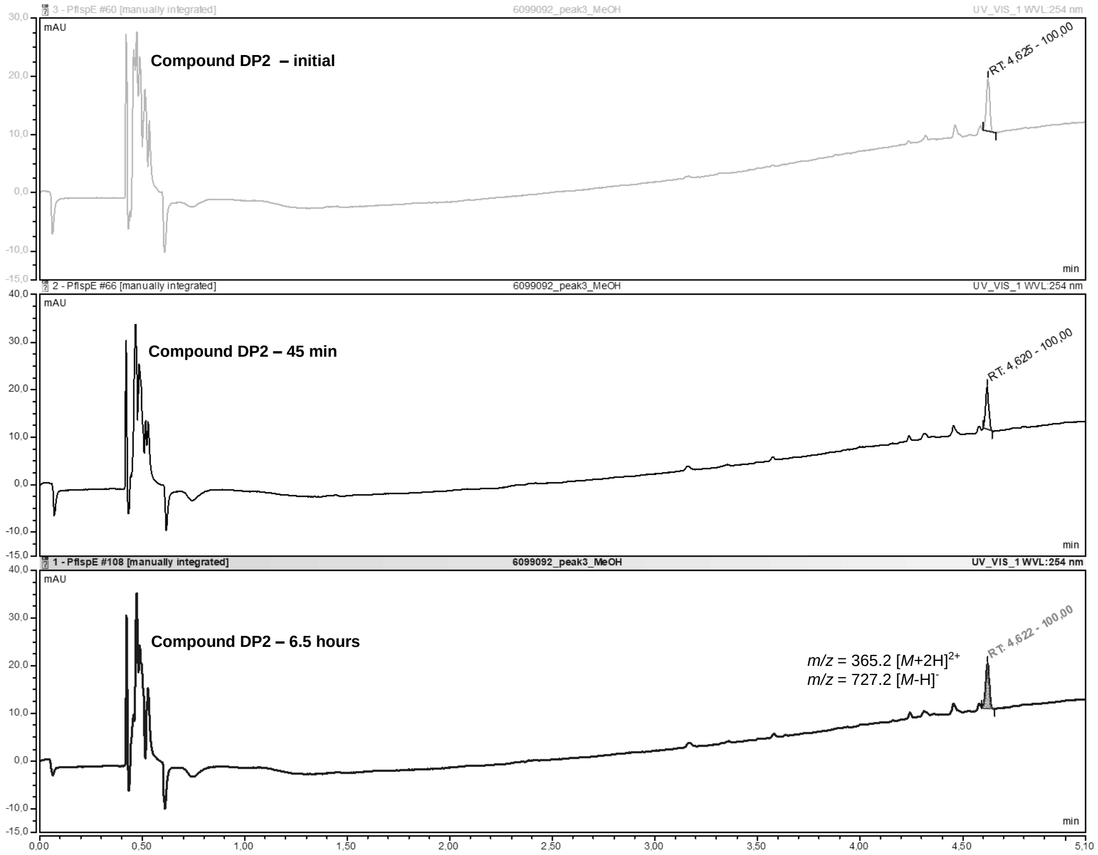

Figure S47. Stability of **DP2** in MeOH and stability check over time.

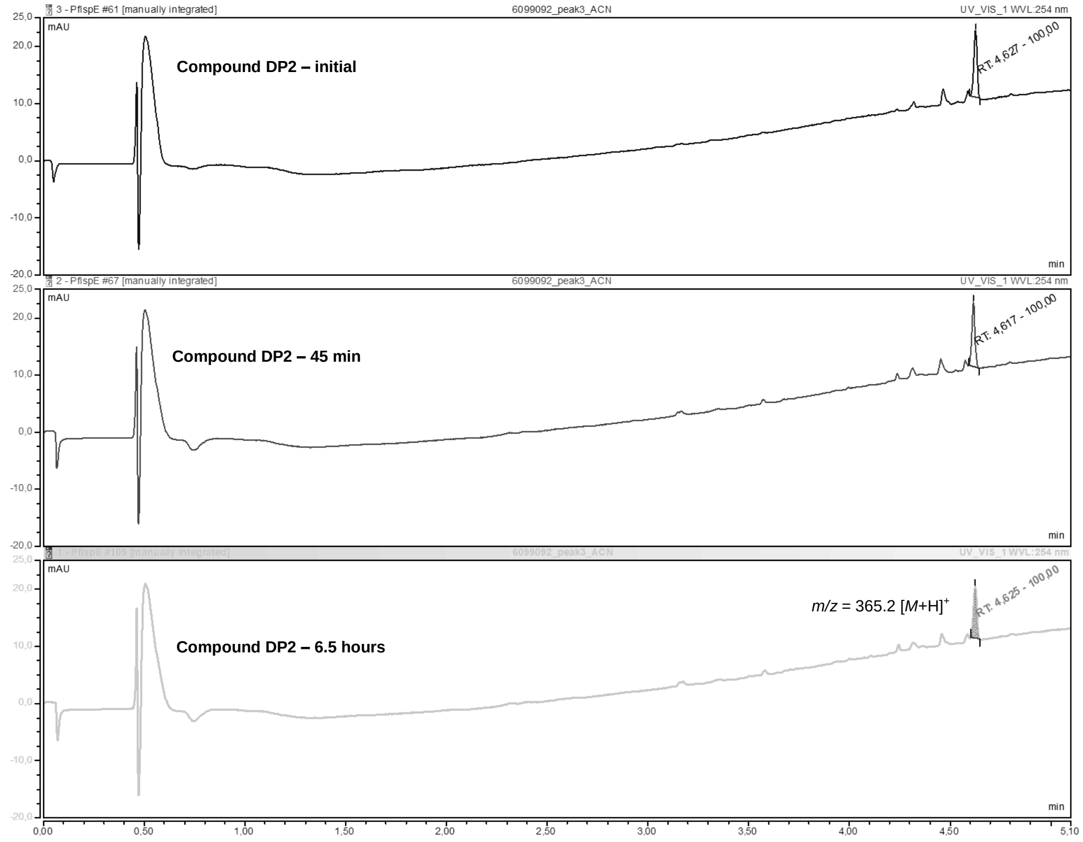

Figure S48. Stability of **DP2** in ACN and stability check over time.

2.9 Screen of the Close Derivatives 2, 3 and 4

4-(1-Ethyl-2,5-dimethyl-1*H*-pyrrol-3-yl)-*N*-(2-methoxyphenyl)thiazol-2-amine (2)

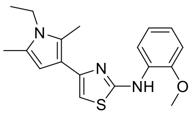

Synthesis as for compound **1**, (0.234 g, 0.715 mmol, 71%).
HRMS (ESI+) calcd. for $C_{18}H_{21}N_3OS$ [*M*+H]$^+$: 328.14331, found: 328.14661.

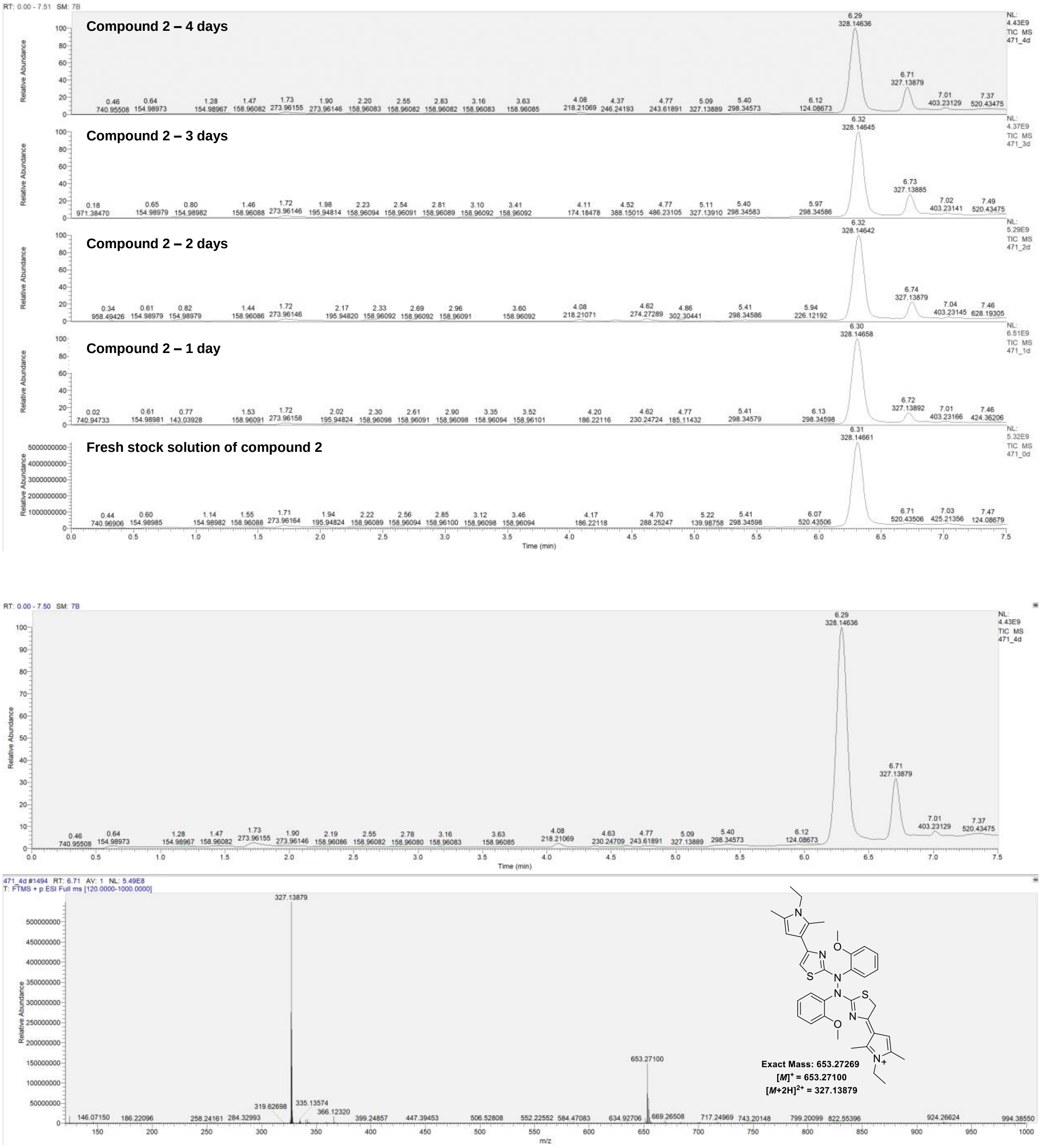

Figure S49. Compound **2** stored in 10 mM DMSO stock solution at room temperature and its HMRS chromatographic profile at the given time points.

4-(1-Allyl-2,5-dimethyl-1*H*-pyrrol-3-yl)-*N*-(2-isopropylphenyl)thiazol-2-amine (3)

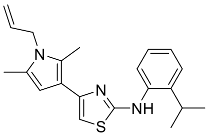

Synthesis as for compound **1**, (0.077 g, 0.219 mmol, 46%).
HRMS (ESI+) calcd. for $C_{21}H_{25}N_3S$ [*M*+H]$^+$: 352.18420, found: 352.18265.

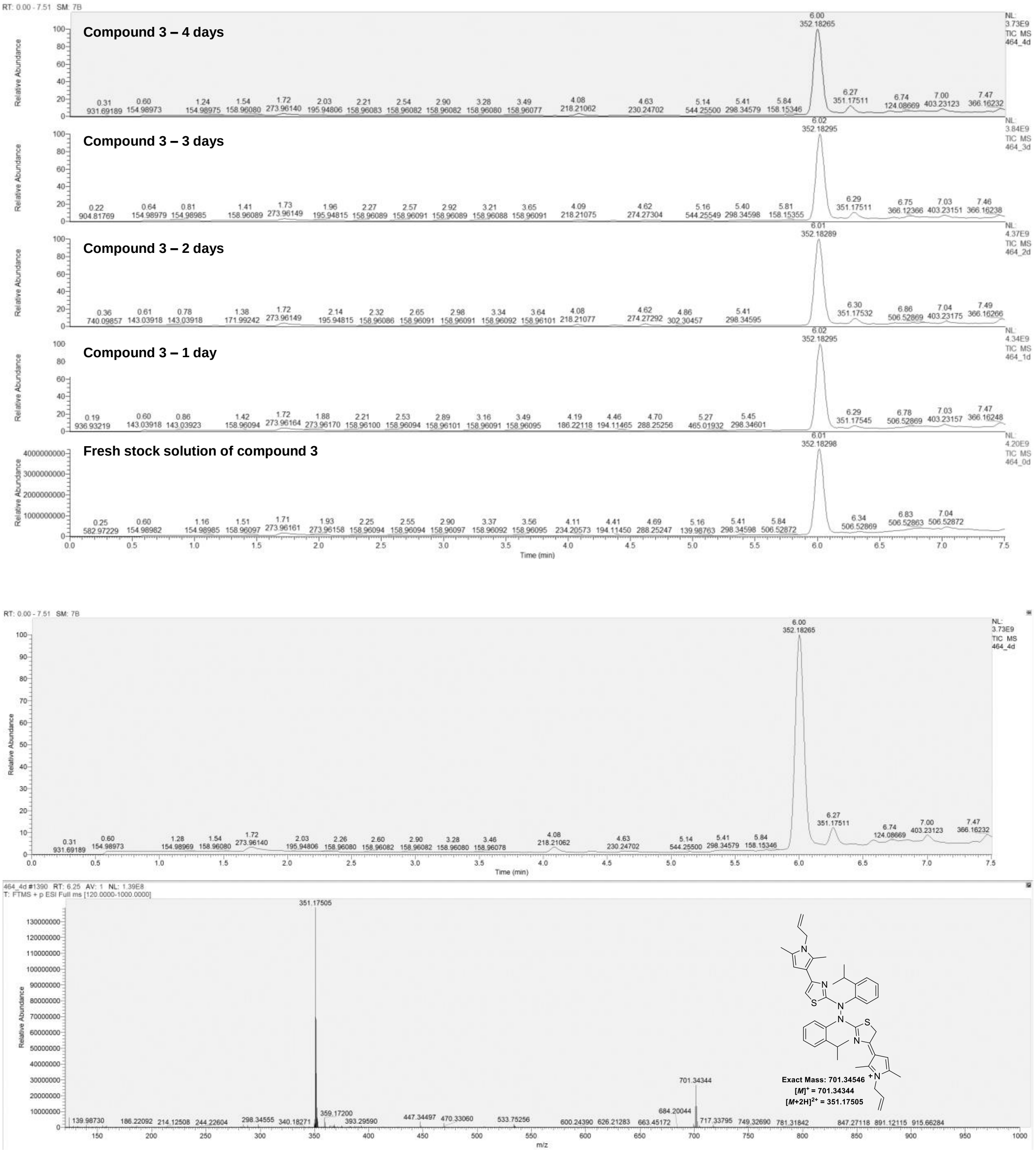

Figure S50. Compound **3** stored in 10 mM DMSO stock solution at room temperature and its HMRS chromatographic profile at the given time points.

4-(2,5-Dimethyl-1-((tetrahydrofuran-2-yl)methyl)-1*H*-pyrrol-3-yl)-*N*-(2-(trifluoromethyl)phenyl)thiazol-2-amine (4)

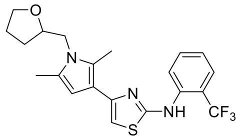

Compoud **4** (CAS 745793-84-4) was purchased and kindly provided by BASF.
HRMS (ESI+) calcd. for $C_{21}H_{22}F_3N_3OS$ [*M*+H]$^+$: 422.15085, found: 422.14951.

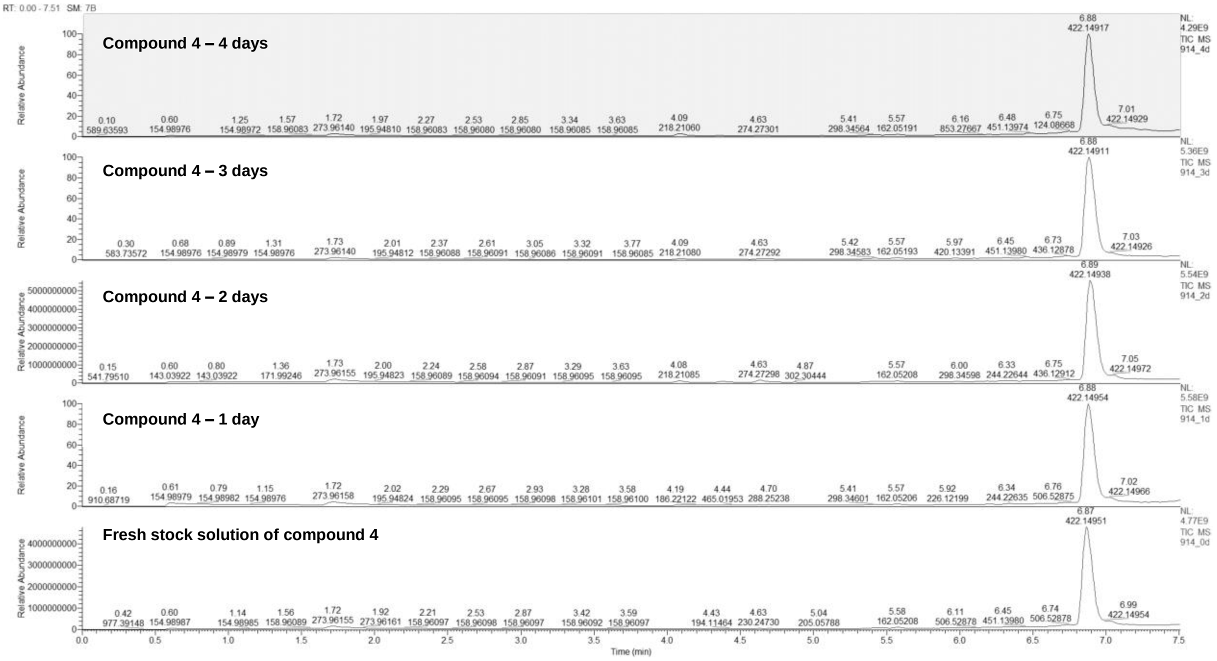

Figure S51. Compound **4** stored in 10 mM DMSO stock solution at room temperature and its HMRS chromatographic profile at the given time points.

3. References

[1] H.-K. Ropponen, E. Diamanti, A. Siemens, B. Illarionov, J. Haupenthal, M. Fischer, M. Rottmann, M. Witschel, A. K. H. Hirsch, *RSC Med. Chem.* **2021**, DOI 10.1039/D0MD00409J.

[2] H. Lüttgen, F. Rohdich, S. Herz, J. Wungsintaweekul, S. Hecht, C. A. Schuhr, M. Fellermeier, S. Sagner, M. H. Zenk, A. Bacher, W. Eisenreich, *Proc. Natl. Acad. Sci. U. S. A.* **2000**, *97*, 1062–1067.

[3] A. K. H. Hirsch, M. S. Alphey, S. Lauw, M. Seet, L. Barandun, W. Eisenreich, F. Rohdich, W. N. Hunter, A. Bacher, F. Diederich, *Org. Biomol. Chem.* **2008**, *6*, 2719–2730.

[4] A. Dorn, R. Stoffel, H. Matile, A. Bubendorf, R. G. Ridley, *Nature* **1995**, *374*, 269–271.

[5] W. Trager, J. B. Jensen, *Science (80-.).* **1976**, *193*, 673–675.

[6] W. Huber, J. C. Koella, *Acta Trop.* **1993**, *55*, 257–261.

[7] J. Haupenthal, C. Baehr, S. Zeuzem, A. Piiper, *Int. J. Cancer* **2007**, *121*, 206–210.

4. Appendix

Appendix I – Calibration of the HRMS Data

Table S6. Calibration curve of the compound **1** with an internal standard at different incubation temperatures.

Component Name	Curve Index	Weighting Index	Origin Index	Equation
Compound 1	Linear	1/X^2	Ignore	Y = -0.375618+9.90624*X R^2 = 0.9890

Filename	Area	ISTD Area	Area Ratio	Amount calculated present (µM)	Amount present (µM)	Specified %Diff	%RSD-AMT	Retention Time	Notes
Cal1	10150520937	108024178	93,965	10,000	9,523	-5%	0,0%	5,74	
Cal2	5529194612	108615825	50,906	5,000	5,177	4%	0,0%	5,73	
Cal3	2950165560	111027345	26,572	2,500	2,720	9%	0,0%	5,73	
Cal4	1490851206	113101576	13,182	1,250	1,369	9%	0,0%	5,74	
Cal5	706935183	116652524	6,060	0,625	0,650	4%	0,0%	5,74	
Cal6	309216919	118967216	2,599	0,313	0,300	-4%	0,0%	5,73	
Cal7	114517300	121534718	0,942	0,156	0,133	-15%	0,0%	5,74	
Cal8	39000722	126381297	0,309	0,078	0,069	-11%	0,0%	5,73	
Cal9	5921925	127751015	0,046	0,039	0,043	9%	0,0%	5,73	
Cal10	*2121888*	*131737338*	*0,016*	*0,020*	*0,040*	*102%*	*NA*	*5,73*	*Excluded*
						Percentage of 7.653			
Initial Compound 1	8415425965	111503864	75,472	NA	7,657	100%	NA	5,72	
Initial Compound 1	8550587377	113394813	75,405	NA	7,650	100%	NA	5,73	
				Average=	7,653				
							**Average of Two Samples**		
1week RT1	3975168172	147432193	26,963	NA	2,760	36%	36%	5,74	
1week RT2	3841533630	144556254	26,575	NA	2,721	36%		5,73	
1week +4 °C1	10527675861	144236464	72,989	NA	7,406	97%	96%	5,74	
1week +4 °C2	10246397426	144141113	71,086	NA	7,214	94%		5,74	
1week -20 °C1	10460128182	144477062	72,400	NA	7,346	96%	96%	5,73	
1week -20 °C2	10664148926	147035848	72,528	NA	7,359	96%		5,73	
1week -20 °C solid1	11336006749	144593718	78,399	NA	7,952	104%	101%	5,73	
1week -20 °C solid2	10450815845	140225592	74,529	NA	7,561	99%		5,73	
1week +4 °C solid1	10077684135	140341550	71,808	NA	7,287	95%	98%	5,73	
1week +4 °C solid2	10705408284	141361442	75,731	NA	7,683	100%		5,73	
1week RT solid1	10929237063	144443787	75,664	NA	7,676	100%	99%	5,73	
1week RT solid2	10341923364	141166150	73,261	NA	7,433	97%		5,72	
1month RT1	921036	1901671	0,484	NA	0,087	1%	1%	5,82	*Lower IS amount, dilution factor x100 used*
1month RT2	1446146	1689226	0,856	NA	0,124	2%		5,71	
1month +4 °C1	7005299232	1745394	4013,593	NA	4,052	53%	48%	5,74	
1month +4 °C2	6110709717	1854816	3294,509	NA	3,326	43%		5,71	
1month -20 °C1	11311078789	1610313	7024,151	NA	7,091	93%	91%	5,72	
1month -20 °C2	11148102857	1643287	6784,027	NA	6,849	89%		5,72	
2months RT1	1918627	1531601	1,253	NA	0,164	0%	0%	5,57	*Column conditions changed, RT shifted*
2months RT2	2022446	1576658	1,283	NA	0,167	0%		5,56	
2months +4C1	30239658	1721642	17,564	NA	1,811	24%	21%	5,80	
2months +4°C2	21706730	1643370	13,209	NA	1,371	18%		5,78	
2months -20°C1	12136948119	1960005	6192,305	NA	6,251	82%	82%	5,79	
2months -20°C2	10261734758	1657331	6191,721	NA	6,251	82%		5,80	
Original Decomposed Sample	28364982	1591587	17,822	NA	1,837	24%	28%	5,80	
Original Decomposed Sample	37865473	1537328	24,631	NA	2,524	33%		5,80	

IS = Diphenhydramine (500 nM)
Retention Time 4.1 min

Appendix II – Extra NMR Spectra of the Other Isolated Fractions

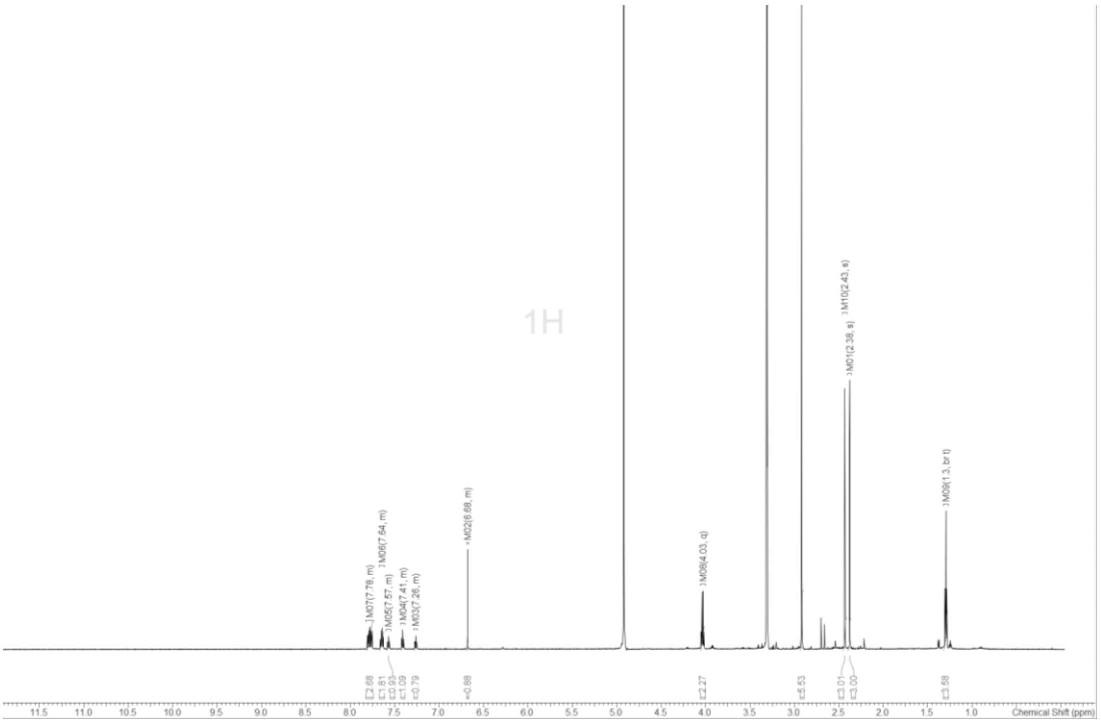

Figure S52. Fraction **4** of the first prep. HPLC separation in methanol-d_4.

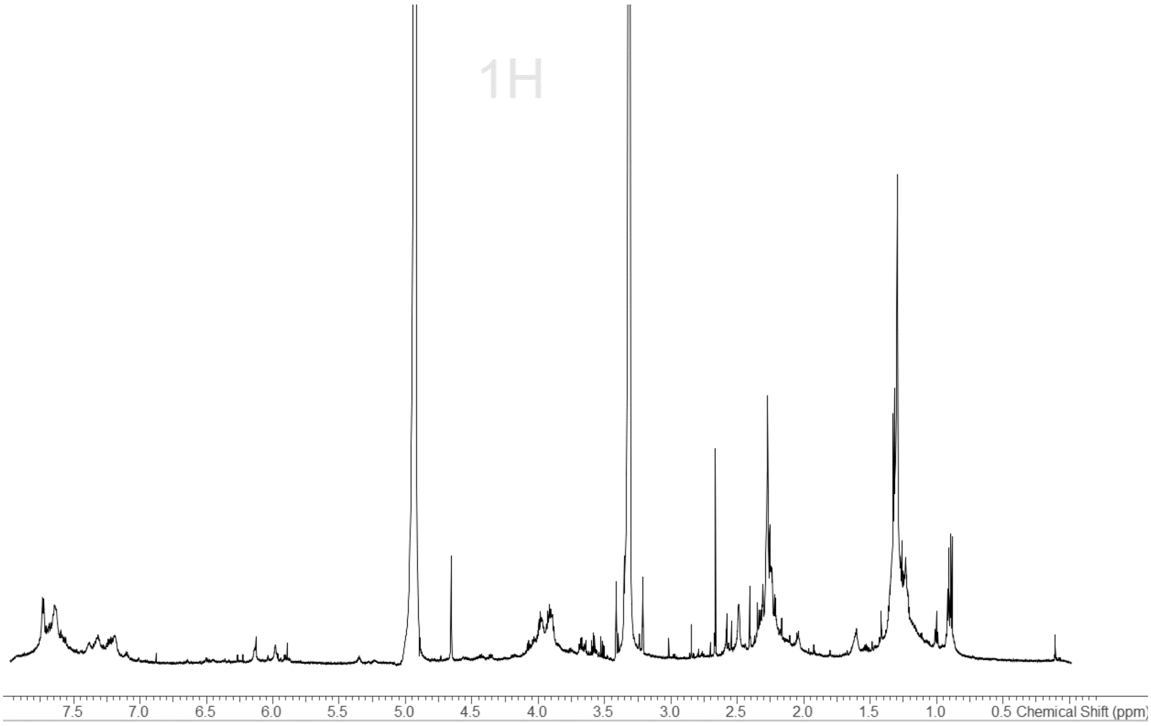

Figure S53. Fraction **31** of the first prep. HPLC separation in methanol-d_4.
